# Acute healthcare resource utilization by age: A cohort study

**DOI:** 10.1371/journal.pone.0251877

**Published:** 2021-05-19

**Authors:** Bourke W. Tillmann, Longdi Fu, Andrea D. Hill, Damon C. Scales, Robert A. Fowler, Brian H. Cuthbertson, Hannah Wunsch

**Affiliations:** 1 Interdepartmental Division of Critical Care Medicine, University of Toronto, Toronto, Ontario, Canada; 2 Department of Critical Care Medicine, Sunnybrook Health Sciences Centre, Toronto, Ontario, Canada; 3 ICES, University of Toronto, Toronto, Ontario, Canada; 4 Sunnybrook Research Institute, Toronto, Ontario, Canada; 5 Department of Medicine, University of Toronto, Toronto, Ontario, Canada; 6 Li Ka Shing Knowledge Institute, St. Michael’s Hospital, Toronto, Ontario, Canada; 7 Department of Anesthesiology and Pain Medicine, University of Toronto, Toronto, Ontario, Canada; University of Notre Dame Australia, AUSTRALIA

## Abstract

**Background:**

Granular data related to the likelihood of individuals of different ages accessing acute and critical care services over time is lacking.

**Methods:**

We used population-based, administrative data from Ontario to identify residents of specific ages (20, 30, 40, etc. to 100) on January 1^st^ every year from 1995–2019. We assessed rates of emergency department (ED) visits (2003–19), hospitalizations, intensive care unit (ICU) admissions (2003–19), and mechanical ventilation.

**Findings:**

Overall the 25-year study period, ED were the most common acute healthcare encounter with 100-year-olds having the lowest rate (138.7/1,000) and 90-year-olds the highest (378.5/1,000). Rates of hospitalization ranged from 24.2/1,000 for those age 20 up to 224.9/1,000 for those age 90. Rates of ICU admission and mechanical ventilation were lowest for those age 20 (1.0 and 0.4/1,000), more than tripled by age 50 (3.3 and 1.7/1,000) and peaked at age 80 (20.3 and 10.1/1,000). Over time rates of ED visits increased (164.3 /1,000 in 2003 vs 199.1 /1,000 in 2019) as did rates of invasive mechanical ventilation (2.0/1,000 in 1995 vs 2.9/1,000 in 2019), whereas rates of ICU admission remained stable (4.8/1,000 in 2003 vs 4.9/1,000 in 2019) and hospitalization declined (66.8/1,000 in 1995 vs 51.5/1,000 in 2019). Age stratified analysis demonstrated that rates of ED presentation increased for those age 70 and younger while hospitalization decreased for all age groups; ICU admission and mechanical ventilation rates changed variably by age, with increasing rates demonstrated primarily among people under the age of 50.

**Interpretation:**

Rates of hospitalizations have decreased over time across all age groups, whereas rates of ED presentation, ICU admissions, and mechanical ventilation have increased, primarily driven by younger adults. These findings suggest that although the delivery of healthcare may be moving away from inpatient medicine, there is a growing population of young adults requiring significant healthcare resources.

## Introduction

Acute healthcare encounters vary by age, with older adults the highest users of most services [1, 2]. Yet, many younger people also require hospital-based services, and small changes in population rates may have large consequences for the healthcare system. An in-depth understanding of the population’s needs for hospital-based services, and particularly high-cost resources such as critical care services, is essential to optimize healthcare planning [3]. Despite the ongoing collection of administrative data, current population-level data on acute healthcare resource utilization are limited [4–6]. Specific organizations, such as the National Health Service (NHS), publish data related to hospital admissions, but considerable gaps exist in relation to temporal trends or utilization of critical care services [7].

Population-level assessments of the impact of aging can be challenging as changes in outcomes at younger ages may mask opposing changes in event rates at older ages [8]. This phenomenon occurs when changes in event rates among the young have a greater impact on estimates of lifetime risk [8, 9]. For example, if infant mortality decreases but mortality related to heart disease increases, estimates of population-level life expectancy improve. Moreover, when populations are analyzed using wide age-bands (e.g. 10 years), changes in event rates within each age-band may simply reflect shifts in population demographics [8, 10]. Failing to account for divergent risks at the population-level and the bias created by changing population demographics can lead to erroneous conclusions regarding temporal trends in care patterns. When applied to the examination of population-trends in resource utilization, these confounding factors make it challenging in estimating the demands that shifts in demographics will place on the healthcare system.

Using comprehensive population-level healthcare data from Ontario, this study aimed to describe the overall patterns of resource utilization for major acute healthcare encounters, including emergency department (ED) presentations and hospitalizations, and critical care encounters, including intensive care unit (ICU) admissions and receipt of mechanical ventilation by age, and assess changes over time.

## Methods

### Study design and population

We used population-based, administrative data from 1995 to 2019 to compare healthcare resource utilization for people at nine distinct ages. We included all residents of Ontario aged 20, 30, 40, 50, 60, 70, 80, 90, or 100 as of January 1^st^ in each year. We restricted the analysis to individual ages to avoid the problem of “aging” within a given age-band over time [10]. Ontario non-residents, individuals without a valid Provincial Health Card, and those missing information on their age or sex were excluded.

### Data source

Data for this study were provided by ICES, a prescribed entity with approval from the Information and Privacy Commissioner of Ontario that holds an inventory of datasets comprising the majority of publicly funded health service records for Ontario [11]. In Ontario, all medically necessary services are publicly funded by a universal single-payer system. As such, the impact of payer status on the decision to seek medical attention is attenuated. Details of the ICES datasets have been described previously and are available in S1 Table [12, 13]. These datasets were linked using unique encoded identifiers and analyzed at ICES.

### Patient characteristics

To provide context for the observed changes in healthcare resource utilization we collected data on baseline population characteristics suspected to be related to healthcare utilization. These characteristics included age, sex, location of primary residence (urban vs. rural), Charlson Comorbidity Score (Deyo modification), history of dementia, history of chronic dialysis, and if they were a resident of a long-term care home [14–17]. We derived the Charlson Comorbidity Score using a 5-year lookback period for hospitalizations, and report the score as well as a separate category for patients with no hospitalizations during the lookback period. The utilization of the discharge abstract database (DAD) for identification of premorbid conditions has been previously validated [13].

### Outcomes

The primary outcomes were presentation to an ED, hospital admission, ICU admission, and receipt of invasive mechanical ventilation. These outcomes were identified using previously validated algorithms [13, 18, 19]. Each outcome of interest was assessed as a binary variable and expressed as an annual rate per 1,000 population. Additionally, to evaluate the overall utilization of healthcare resources, the total number of occurrences per year was also calculated. Other outcomes included annual number of ED visits among people who presented to an ED, annual number of hospital admissions among people who were hospitalized, total days spent in hospital among hospitalized patients, total duration of ICU admission(s) among those admitted to the ICU, and death (identified using the Registered Persons Database, which contains vital statistics on all Ontario residents).

### Statistical analysis

We used descriptive statistics to display baseline population characteristics and outcomes. Continuous variables were reported as medians with interquartile ranges (IQR) and categorical variables as counts and percentages. Standardized differences were used to compare characteristics between the population in 1995 and 2019.

To provide a summary estimate of the temporal trends in resource utilization we calculated the estimated annual percent change (EAPC) for the rate of each outcome of interest using a negative binomial model. The outcome of these models was the annual number of people who experienced at least one event (ED presentation, hospital admission, ICU admission, receipt of mechanical ventilation, death). As the goal of this study was to quantify population-level rates and changes in the pattern of overall healthcare resource utilization, as opposed to the risk associated with specific disease states or sociodemographic conditions, we purposely did not employ multivariable modeling to adjust the estimates. Therefore, the only dependent variable in the model was the year. To account for changes in the population during the study period, the natural log of the population for each year was utilized as the offset in all models estimating rates. Additionally, to quantify shifts in the risk of experiencing an acute care encounter over time, we calculated the relative change in the rates (RR) of hospital admissions and receipt of invasive mechanical ventilation, comparing rates in 2019 to 1995. Standardized data related to ED presentations and ICU admissions were not routinely collected province wide until 2003, therefore the RR of ED visits and ICU admissions were calculated using 2003 as the baseline comparator.

All analyses were performed on the entire cohort as well as stratified by age band. Standardized differences >0.10 and two-sided p-values of <0.05 were considered significant [20]. No adjustments were made for multiple comparisons. All statistical analyses were done using SAS version 9.4 (SAS Institute Inc., Cary NC). The study was approved by the ICES Privacy and Compliance Office.

## Results

### Study population

The total cohort consisted of 32,010,187 people (Table 1), and each year of analysis included more than one million people. The majority of individuals were within the first three age groups (18.1% age 20, 18.8% age 30, and 19.0% age 40), approximately half of the cohort was female (50.7%), and 4.9% of the population had an identified comorbid illness (Table 1). Over time the proportion of patients without a hospitalization in the previous five years increased. A comparison of the individuals assessed in 2019 to those in 1995 was consistent with an increasing, and aging population, with a substantial shift in the proportion of the cohort in the older age categories, and more with dementia.

**Table 1:** Population baseline characteristics.

	Overall (n = 32,010,187)	1995 (n = 1,113,313)	2003 (n = 1,250,737)	2011 (n = 1,366,196)	2019 (n = 1,396,904)	Standardized difference*
Age, n (%)						
20	5,778,956 (18.1)	235,846 (21.2)	244,253 (19.5)	247,436 (18.1)	183,398 (13.1)	0.21
30	6,022,233 (18.8)	276,816 (24.9)	241,924 (19.3)	232,742 (17.0)	239,566 (17.1)	0.19
40	6,084,263 (19.0)	217,901 (19.6)	271,765 (21.7)	248,833 (18.2)	223,020 (16.0)	0.09
50	5,476,221 (17.1)	144,232 (13.0)	199,127 (15.9)	255,580 (18.7)	242,927 (17.4)	0.12
60	3,955,368 (12.4)	102,287 (9.2)	129,599 (10.4)	177,835 (13.0)	229,591 (16.4)	0.22
70	2,640,018 (8.2)	83,919 (7.5)	91,317 (7.3)	106,451 (7.8)	160,102 (11.5)	0.13
80	1,515,984 (4.7)	42,074 (3.8)	57,272 (4.6)	70,269 (5.1)	80,781 (5.8)	0.09
90	488,736 (1.5)	9,766 (0.9)	14,525 (1.2)	24,508 (1.8)	32,979 (2.4)	0.12
100	48,408 (0.2)	472 (<0.1)	955 (0.1)	2,542 (0.2)	4,540 (0.3)	0.07
Female, n (%)	16,215,763 (50.7)	566,110 (50.8)	634,582 (50.7)	691,540 (50.6)	704,429 (50.4)	0.01
Charlson comorbidity score, n (%)						
0	3,818,598 (11.9)	184,954 (16.6)	149,700 (12.0)	146,588 (10.7)	143,768 (10.3)	0.18
1–2	1,146,940 (3.6)	45,103 (4.1)	45,439 (3.6)	45,492 (3.3)	52,092 (3.7)	0.02
≥3	404,381 (1.3)	11,970 (1.1)	14,834 (1.2)	18,367 (1.3)	21,458 (1.5)	0.04
No hospitalizations within previous 5yr	26,640,268 (83.2)	871,286 (78.3)	1,040,764 (83.2)	1,155,749 (84.6)	1,179,586 (84.4)	0.16
Chronic dialysis, n (%)	22,502 (0.1)	420 (<0.1)	775 (0.1)	1,056 (0.1)	1,360 (0.1)	0.02
History of dementia, n (%)	318,149 (1.0)	5,148 (0.5)	9,976 (0.8)	16,531 (1.2)	18,768 (1.3)	0.09
Resident of a long-term care facility, n (%)	200,136 (0.6)	6,317 (0.6)	7,082 (0.6)	9,358 (0.7)	9,336 (0.7)	0.01
Rural residence, n (%)	3,389,452 (10.6)	138,205 (12.4)	138,766 (11.1)	139,380 (10.2)	134,914 (9.7)	0.09

*Comparing 2019 with 1995.

### Acute healthcare and critical care encounter rates by age

**Fig 1:**
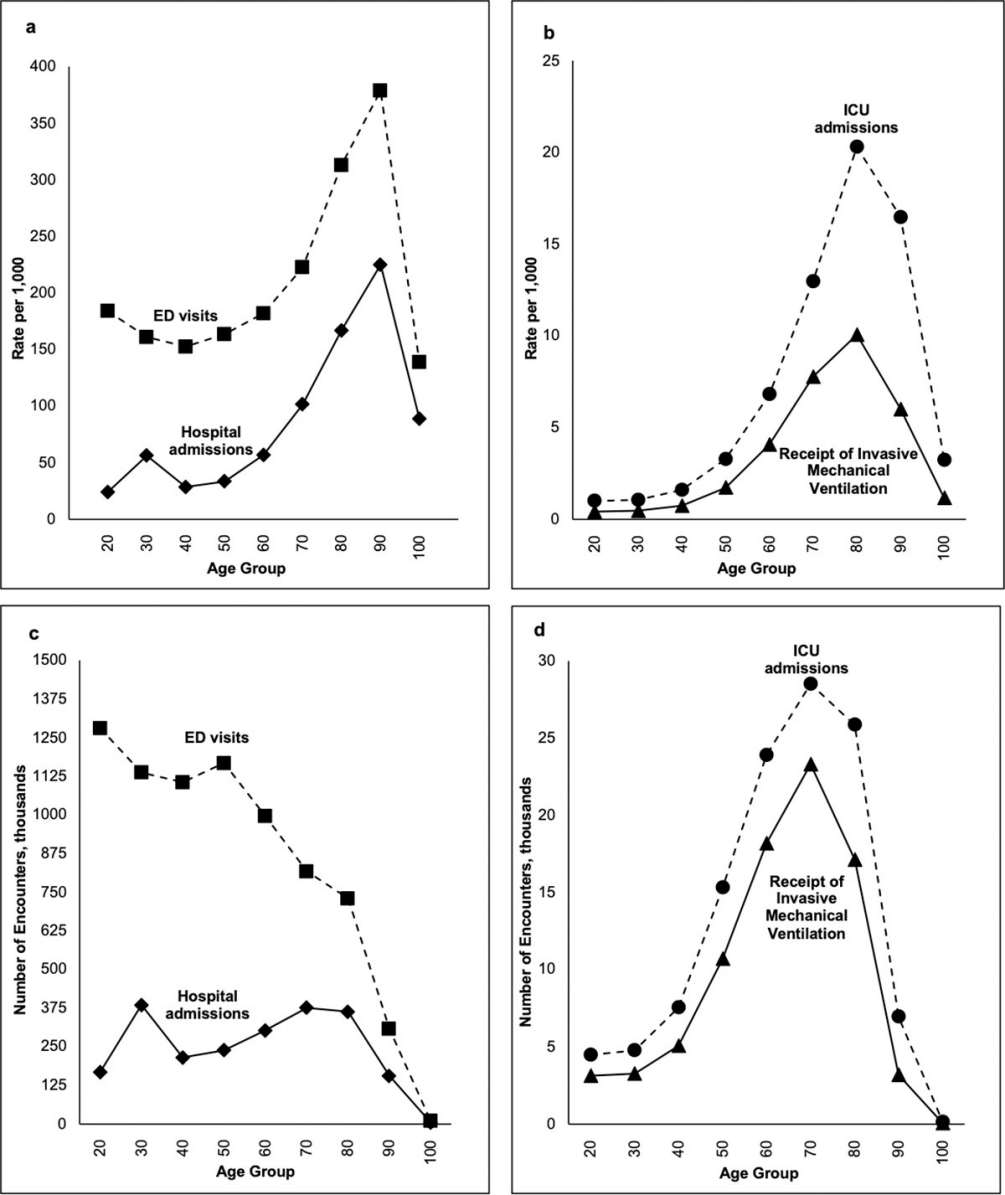
Age stratified trends in overall rates of: a) acute healthcare encounters; and b) critical care encounters, and overall volume of: c) acute healthcare encounters; and d) critical care encounters.

**Fig 2:**
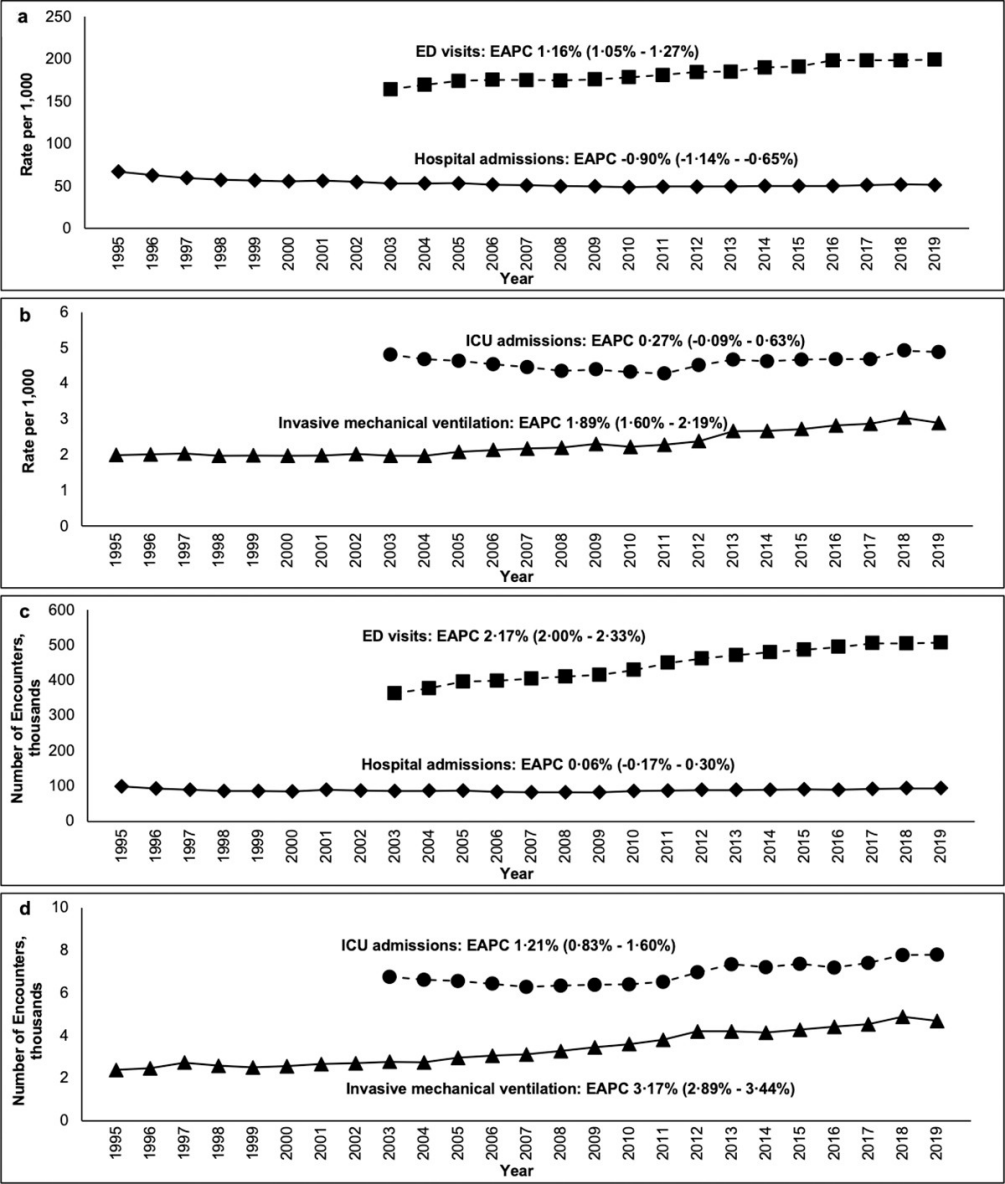
Secular trends in overall rates of: a) acute healthcare encounters; and b) critical care encounters, and overall volume of: c) acute healthcare encounters; and d) critical care encounters.

Across the entire cohort, rates of all events increased with age (Fig 1A and 1B). For ED visits and hospitalizations, the peak was age 90 (378.5 per 1,000 for ED visits and 224.9 per 1,000 for hospital admissions), while for ICU admissions and invasive mechanical ventilation the peak was age 80 (20.3 per 1,000 for ICU and 10.1 per 1,000 for invasive mechanical ventilation). Details related to mortality are available in the S1 Fig. Of note, rates of ED presentations were markedly higher for those age 20 vs those age 30, 40, or 50, and hospital admission rates increased substantially for those age 30 relative to age 20 or 40 (Fig 1A). The increase in hospital admission rates for 30-year-olds appeared driven by admissions among women (S2 Table). As patients could utilize any single healthcare resource multiple times per year, overall annual rates for each outcome of interest were greater than an individual’s rate of resource utilization (S2 Fig, Fig 2A and 2B). However, the peaks of resource utilization occurred at the same ages (ED visits at age 90, 776.3 per 1,000; hospitalization at age 90, 319.1 per 1,000; ICU admissions at age 80, 22.4 per 1,000; and receipt of invasive mechanical ventilation at age 80, 11.3 per 1,000).

### Absolute volume of acute healthcare and critical care encounters by age

Although 80- and 90-year-olds had the highest rates of healthcare resource utilization, over the entire study period 20-year-olds represented the largest volume of people who presented to the ED, and 30-year-olds the largest volume of people admitted to hospital (Fig 1C). Furthermore, 70-year-olds represented the largest volume of people admitted to the ICU as well as people who received invasive mechanical ventilation (Fig 1D). Again, the high volume of 30-year-olds experiencing hospital admissions appeared driven by women (S2 Table).

### Acute healthcare encounter rates over time

Between 2003 to 2019 there was a 21.2% relative increase in the rate at which a person visited the ED at least once pear year (RR 1.21, 95% CI; 1.21–1.22), from 164.3 to 199.1 per 1,000 people (EAPC 1.16%, 95% CI; 1.05–1.27%), (Fig 2A). Conversely, there was a nearly 25% relative decrease in the annual rate of people experiencing at least one hospital admission (RR 0.77, 95% CI; 0.76–0.78) from 66.8 per 1,000 in 1995 to 51.5 per 1,000 in 2019 (EAPC -0.90%, 95% CI; -1.14%–-0.65%). There was a similar decrease in the overall annual rate of people admitted to a hospital during the study period (RR 0.75, 95% CI; 0.75–0.76; EAPC -0.99%, 95% CI; -1.30%–-0.69%), (S2C Fig).

### Critical care rates over time

The rate at which a person was admitted to the ICU at least once per year was relatively stable from 2003 to 2019 (4.8 per 1,000 in 2003 to 4.9 in 2019; RR 1.01, 95% CI; 0.95–1.05; EAPC 0.27%, 95% CI; -0.09–0.63%), (Fig 2B). However, there was a slight increase in the overall annual rate of ICU admissions in 2019 relative to 2003 (5.6 per 1,000 in 2003 to 5.4 per 1,000 in 2019; RR 1.03; 95% CI, 1.01–1.06.), (S2D Fig). Unlike the trends in ICU admissions, rates of invasive mechanical ventilation increased significantly from 2.0 per 1,000 in 1995 to 2.9 per 1,000 in 2019 (RR 1.46, 95% CI; 1.38–1.54; EAPC 1.89%, 95% CI; 1.60–2.19%). Likewise, the overall annual rate of people receiving mechanical ventilation was 56.1% greater in 2019 relative to 1995 (RR 1.56, 95% CI; 1.51–1.61, EAPC 2.02%, 95% CI; 1.70–2.34%).

### Absolute volume of healthcare encounters over time

The absolute number of acute and critical care encounters increased over time for all events except hospitalizations, which, despite an increase in the underlying population, remained static throughout the study period (Fig 2C and 2D).

### Acute healthcare encounters by age over time

**Fig 3:**
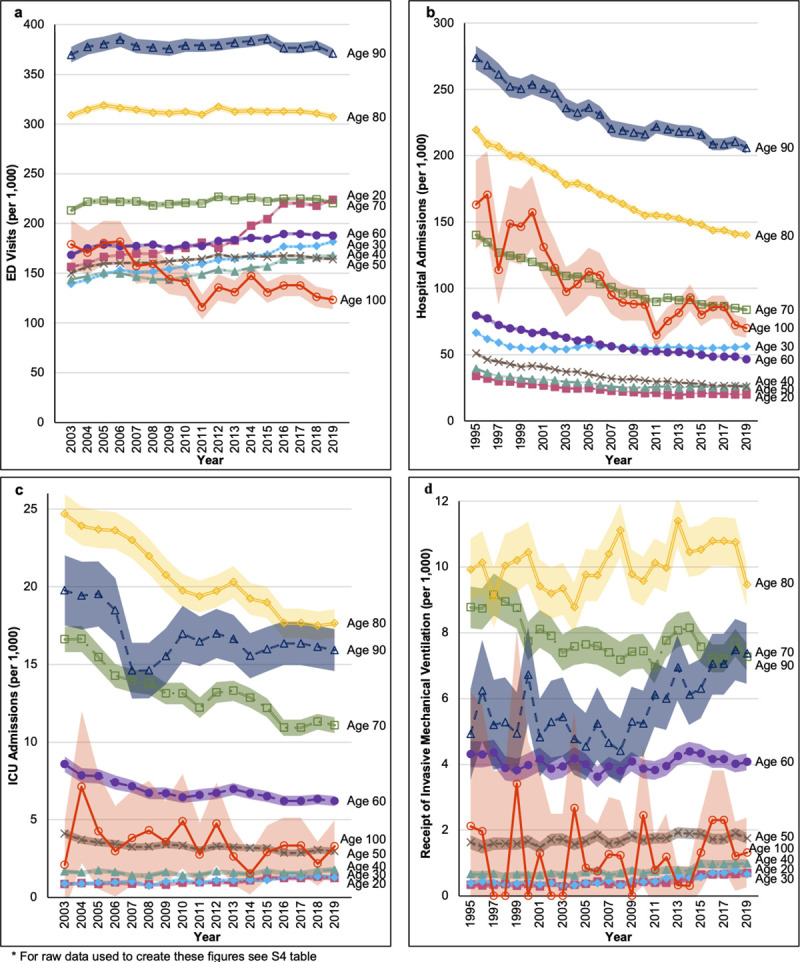
Secular trends in age stratified rates of: a) emergency department visits; b) hospital admissions c) intensive care unit admissions; and d) receipt of invasive mechanical ventilation.

**Fig 4:**
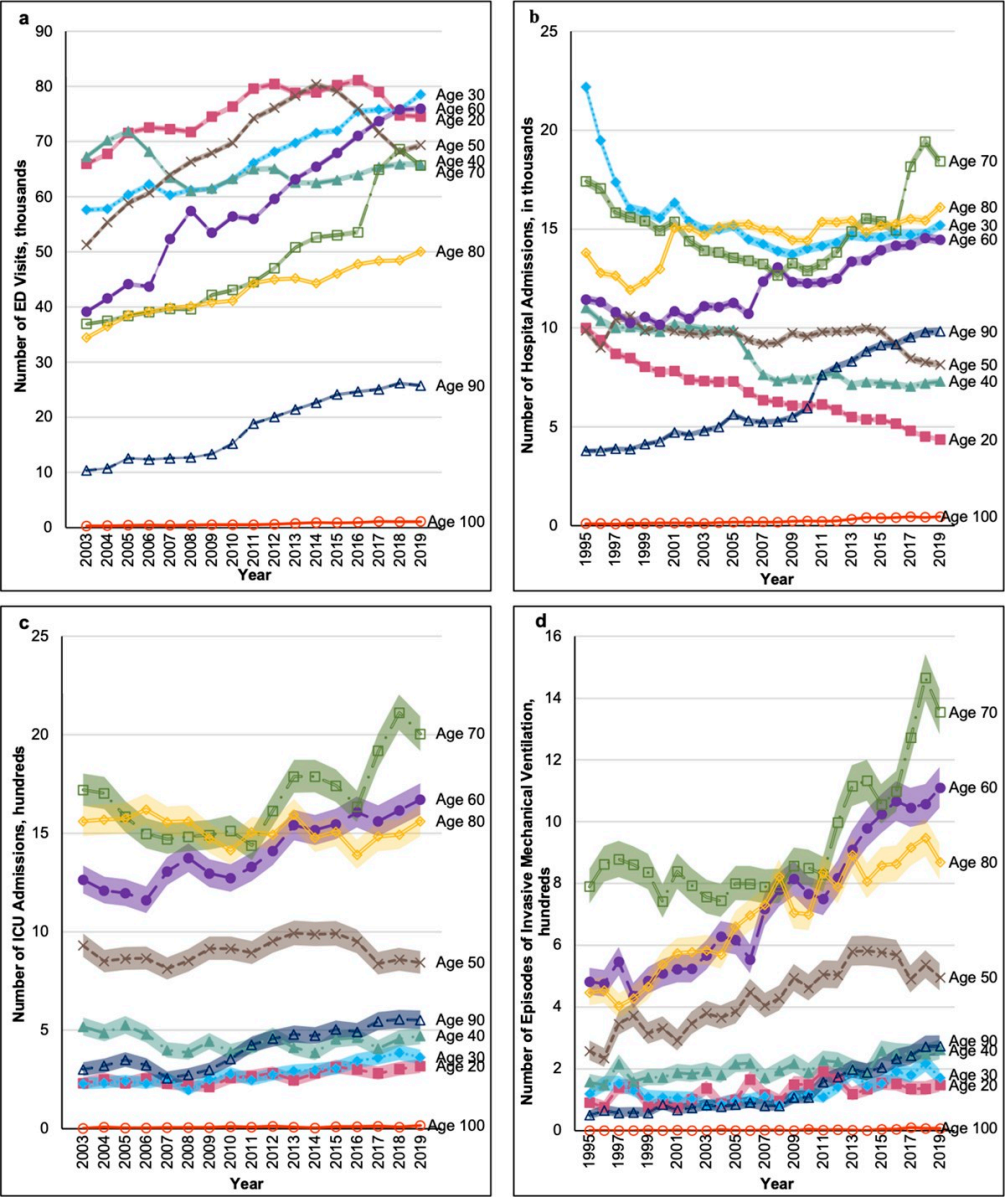
Secular trends in age stratified volumes of: a) emergency department visits; b) hospital admissions c) intensive care unit admissions; and d) receipt of invasive mechanical ventilation.

Although overall rates of ED visits were highest among patients aged 80 or 90, increases in the rates of ED visits were demonstrated primarily among those aged 70 and below (Fig 3A and S5A Table). Conversely, the volume of people presenting to the ED increased over time in all groups, except 40-year-olds (Fig 4A and S5B Table).

Rates of hospital admissions decreased over time in all age groups (Fig 3B). Likewise, among those who were hospitalized the median number of days spent in hospital per year decreased for all ages throughout the study (S6 Table). Hospital length of stay was greatest among 90- and 100-year-olds in 1995 (median 11 days, IQRs 5–23 and 4–18 respectively) decreasing to a median of 8 days (IQR 4–19 among 90-year-olds and 4–17 among 100-year-olds) in 2019. Sex stratified analysis demonstrated that the relatively higher rates of hospital admissions among 30-year-olds were driven by women, whereas rates of hospital admission among men in the 30-year age-band followed a similar pattern to the trends seen in other age-bands (S3 Fig).

Although temporal trends in the rates of hospital admissions were consistent across all age groups, trends in the absolute number of patients admitted to hospital varied across ages (Fig 4B). While patients in the 30-year age-band accounted for the third largest group of admissions, this again was driven primarily by female patients (S4 Fig). Aside from patients in the 30-year age-band, adults between the ages of 60 to 80 comprised the bulk of hospital admissions.

### Critical care admissions by age over time

The rate of ICU admission over time varied by age (Fig 3C), with increases in rates for patients age 20 or 30, and decreasing rates for those age 50 and over. Trends in the volume of ICU admissions were significantly different compared to trends in the rates of ICU admissions (Fig 4C). From 2003 to 2019 the crude volume of ICU admissions increased among the majority of age groups. Similar to the trends in the rates of ICU admissions, across the entire study period adults aged 60 to 80 consistently comprised the largest volume of ICU admissions.

Rates of invasive mechanical ventilation increased over time among the majority of age groups, with the greatest increases seen among those aged 20 to 40 (Fig 3D). Despite these trends, rates of invasive mechanical were highest among people aged 60 to 80 throughout the study period. Examination of the absolute number of people receiving invasive mechanical ventilation demonstrated that volumes were increasing for all groups, with the largest relative increases seen among those age 60, 90, and 100 (Fig 4D).

## Interpretation

In this analysis of 25 years of province-wide, administrative data, we demonstrated divergent trends in acute healthcare and critical care encounters. While rates of hospital admissions decreased over time, rates of ED presentations, and receipt of invasive mechanical ventilation increased. These increasing rates were not consistent across all age groups, with the largest increases in ED visits and mechanical ventilation demonstrated among those age 40 and younger, and static or decreasing rates in these events among most older adults. Additionally, we demonstrated that although an individual patient’s risk of being admitted to a hospital decreased over time, there has been no change in the absolute number of people admitted to the hospital. Finally, while younger adults accounted for the greatest number of people presenting to the ED, adults over age 50 consistently accounted for the greater volume of inpatient resource utilization.

Our findings provide important information on trends in healthcare delivery while accounting for the aging population, and also provide concrete numbers regarding the likelihood of specific health-related events for individuals of different ages. For example, in 2019, slightly more than 20% of 90-year-olds in Ontario were hospitalized at least once. Consequently, as compared to 1995, the probability of hospitalization for a 90-year-old had decreased by 25% in 2019. However, given increases in the population of older adults across the province of Ontario, during this time period there were an additional 6,409 hospitalizations among 90-year-olds. These findings demonstrate that even though the likelihood of a 90-year-old being admitted to hospital decreased, the healthcare system needed to accommodate an increase in the overall volume of patients in this age group. It is plausible that the decreasing duration of hospitalizations demonstrated over time in our study represents one potential response to an increasing demand for acute healthcare resources.

Our finding of an increase in both the volume and the overall rate of patients presenting to the ED highlights concerns regarding the strain faced by the healthcare system [21–24]. Although the lack of an associated increase in hospital admissions may suggest that the increased ED presentations represent subacute conditions, the risks associated with ED overcrowding are related to the number of occupied beds, not patient acuity [25]. Likewise, an increase in utilization of ED resources highlights additional concerns in the healthcare system, including lack of access to primary care, lack of confidence in the care provided in the ambulatory setting, and socioeconomic disparities in access to care [26–29]. The finding that rates of ED visits are increasing across nearly all age groups suggests that the strain in the healthcare system is not related to one specific population. However, our finding that increases in rates of ED presentations were greatest among young adults may suggest that there are specific barriers related to young people accessing primary care.

While hospital overcrowding has been a focus of worldwide concern, we found that over the past 25 years in Ontario per capita hospital admission rates have decreased by 25%. Our finding must be examined within the context of the healthcare system and growing population. Between 1995 to 2014 the number of acute care beds in Ontario decreased by 25% [30, 31]. Likewise, despite our findings of a decrease per capita rate of hospital admissions, during this same time period there was no significant decrease in the absolute number of patient admitted to hospital. Our apparent conflicting findings, of a decreasing rate of hospital admissions without a decrease in the absolute number of patients admitted to hospital are related to the increases in the population of Ontario over the past 25 years; the decreasing rate of hospital admissions has been matched by the increasing population. Moreover, as reductions in rates of hospital admissions appear to be slowing over time, our findings suggest that additional inpatient resources are needed.

Unlike rates of hospital admissions, we found minimal differences in the overall rates of ICU admissions over time. However, in the context of an increasing population this finding masks an increase in the absolute number of patients admitted to an ICU each year. Previous evidence has demonstrated that over the past decade the number of ICU beds in the United States increased by 18%, while critical care occupancy rates remained unchanged [6]. It is possible that the lack of change in ICU occupancy is a result of supply driving demand, in which excess availability of critical care resources leads to increased use of critical care beds for lower risk patients [32]. However, the increasing proportion of patients receiving invasive mechanical ventilation demonstrated in our study suggests the opposite; that illness severity within ICUs is increasing and the steady rates of critical care occupancy indicates the presence of an unmet need for critical care resources. Furthermore, although our age stratified analysis demonstrated that rates of ICU admission are decreasing among older adults, the absolute number of older adults admitted to the ICU is increasing over time. This finding suggests that small changes in individuals’ likelihood of being admitted to an ICU are not translating to decreases in demand for these resources across the healthcare system. Likewise, our analysis of trends in receipt of invasive mechanical ventilation demonstrated that both the rates of invasive mechanical ventilation and the overall number of people receiving invasive mechanical ventilation are increasing in almost all age groups. Together these findings further strengthen the argument that additional critical care resources are likely required to support an increasing and aging population.

This study has several important limitations. First, we only assessed resource utilization rates for patients of specific ages, and therefore do not have information on patients between the analyzed age groups. Given the selected ages spanned the adult lifecycle, and more than 32 million individuals were identified, we would not expect to have missed significant trends. Second, we focused on acute hospital-based resource utilization and are not able to comment on the utilization of primary care over the study period. However, hospital services currently represent the largest area of health expenditure and hospital encounters often represent key, disruptive events in peoples’ lives [33]. Third, we did not obtain information regarding the geographic location of hospitalizations. Consequently, we are unable to describe the changes in resource utilization across urban and rural areas or identify areas of specific concern. However, discrepancies in access to care related to geographic location have been previously described [34]. Fourth, we purposefully chose not to perform any adjustment to account for potential confounders in the relationship between time and our outcomes. This decision was made as the goal of the study was to examine the change in trends of healthcare resource utilization at the population level. Therefore, this study is able to clearly demonstrate a decrease in overall hospital admissions for almost all ages, but is unable to provide data on specific patient cohorts or support assumptions of casual inference. Finally, this analysis was restricted to the population of Ontario. Consequently, the trends seen in resource utilization may not be generalizable to all populations. In Ontario, inpatient healthcare is funded by a universal single payer system potentially limiting the impact of payer status on the decision to seek healthcare. Likewise, Ontario encompasses an area of 415,000m^2^ (about 1.5 times the size of Texas). Accordingly, there are unique geographic challenges which may impact access to healthcare, challenges which may not exist in denser geographic regions.

## Conclusion

Over the past 25 years the rate of people admitted to hospitals in Ontario has decreased, however the absolute number of people admitted to the hospital has remained unchanged. Simultaneously, rates of people presenting to the ED and/or receiving invasive mechanical ventilation have increased, with the largest increases seen among people between the ages of 20–40. These findings suggest that although the delivery of healthcare may be moving away from inpatient medicine, there is a growing population of young adults requiring aggressive life supporting measures. Moreover, as decreases in rates of hospitalization already appear to be slowing, additional inpatient resources are likely needed to support the growing number of people over the age of 60.

## Supporting information

S1 Fig Trends in mortality rates. (DOCX)

S2 Fig Trends in overall rates of acute healthcare and critical care resource utilization. (DOCX)

S3 Fig Trends in rates of hospital admissions by age over time stratified by sex. (DOCX)

S4 Fig Annual number of hospital admissions by age over time stratified by sex. (DOCX)

S1 Table Details of the individual ICES datasets. (DOCX)

S2 Table Proportion of people experiencing an acute healthcare or critical care encounter per year stratified by age and sex. (DOCX)

S3 Table Proportion of the population experiencing at least one major healthcare encounter per year. (DOCX)

S4 Table Annual rates of acute healthcare and critical care resource utilization stratified by age. (DOCX)

S5 Table Estimated annual percent change; a) Rates of acute healthcare and critical care encounters stratified by age; b) Absolute number of acute healthcare and critical care encounters stratified by age.
(DOCX)

S6 Table Annual number of events and total duration of events among patients who experienced at least one major healthcare encounter. (DOCX)

S1 Appendix Dataset creation plan. (DOCX)

S2 Appendix SAS code for cohort creation. (SAS)

S3 Appendix SAS code for analysis. (SAS)
