# Supplementary material for: Acute healthcare resource utilization by age: A cohort study
Source: PLoS One. 2021 May 19;16(5):e0251877. doi: 10.1371/journal.pone.0251877 (PMC8133481; doi:10.1371/journal.pone.0251877)

**S1 Fig.** Trends in mortality

1. Overall mortality rates across the entire cohort stratified by age

1. Overall number of deaths by age

1. Mortality rates over time

1. Overall number of deaths by year

1. Mortality rates by age over time

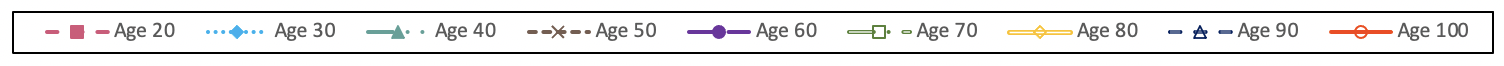


1. Absolute number of deaths by age over time

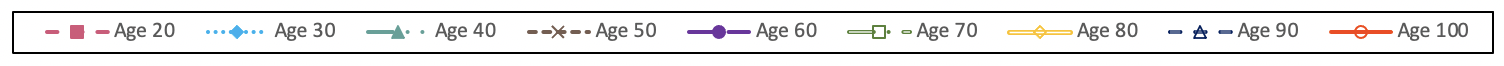

Supplement: S1 Fig — (DOCX) [file pone.0251877.s001.docx]
