# Supplementary material for: Acute healthcare resource utilization by age: A cohort study
Source: PLoS One. 2021 May 19;16(5):e0251877. doi: 10.1371/journal.pone.0251877 (PMC8133481; doi:10.1371/journal.pone.0251877)
Supplement: S2 Fig — (DOCX) [file pone.0251877.s002.docx]

**S2 Fig.** Trends in overall rates of acute healthcare and critical care resource utilization

1. Overall mortality rates of ED visits and hospital admissions across the entire cohort stratified by age

1. Overall rates of ICU admissions and receipt of invasive mechanical ventilation across the entire cohort stratified by age

1. Overall rates of ED visits and hospital admissions across the entire cohort stratified by year

1. Overall rates of ICU admissions and receipt of invasive mechanical ventilation across the entire cohort stratified by year
