# Supplementary material for: Acute healthcare resource utilization by age: A cohort study
Source: PLoS One. 2021 May 19;16(5):e0251877. doi: 10.1371/journal.pone.0251877 (PMC8133481; doi:10.1371/journal.pone.0251877)

**S3 Fig.** Trends in rates of hospital admissions by age over time stratified by sex

1. Trends in rates of hospital admissions for men by age over time

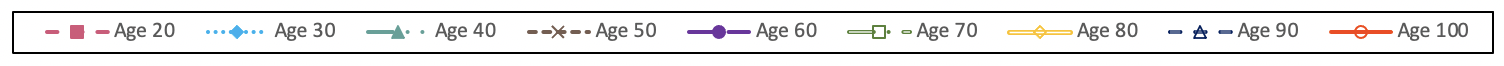


1. Trends in rates of hospital admissions for women by age over time

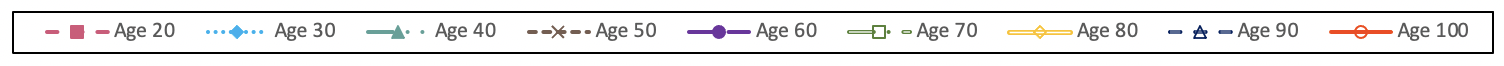

Supplement: S3 Fig — (DOCX) [file pone.0251877.s003.docx]
