# Supplementary material for: Acute healthcare resource utilization by age: A cohort study
Source: PLoS One. 2021 May 19;16(5):e0251877. doi: 10.1371/journal.pone.0251877 (PMC8133481; doi:10.1371/journal.pone.0251877)
Supplement: S1 Table — (DOCX) [file pone.0251877.s005.docx]

**S1 Table.** Details of the individual ICES datasets

| **Dataset*** | **Description** | **Extracted Variables** |
| --- | --- | --- |
| Registered Persons Database (RPDB) | Contains demographic information on all residents of Ontario with a health card | Vital statistics, including age, sex, rural status, socio-economic status, and mortality data |
| Discharge Abstract Database (DAD) | Contains information on all acute care hospitalizations in Ontario | Comorbidity data, hospital admission, and intensive care unit admission |
| National Ambulatory Care Reporting System (NACRS) | Contains information on all emergency department visits in Ontario | Emergency department visits |
| Continuing Care Reporting System (for Chronic Care) (CCRS) | Contains demographic, clinical, functional, and resource utilization data on individuals receiving continuing care services in hospitals or long-term care homes in Ontario | Resident of long-term care facility |
| Client Profile Database (CPRO) | Contains information on individuals placed or waiting to be placed in a long-term care home in Ontario, including demographic information and choice of home | Resident of long-term care facility |
| Ontario Health Insurance Plan (OHIP) Claims database | Captures physician services and encounters through billing claims | History of chronic dialysis and receipt of invasive mechanical ventilation  Codes utilized to identify receipt of invasive mechanical ventilation included:   - G557 – day 1 comprehensive care - G558 – day 2-30 comprehensive care - G559 – day 31 and onward comprehensive care - G405 – day 1 intensive care management of mechanical ventilation - G406 – day 2-30 intensive care management of mechanical ventilation - G407 – day 31 and onward intensive care management of mechanical ventilation |

* Datasets were linked using an encoded unique patient identification number
