# Supplementary material for: Acute healthcare resource utilization by age: A cohort study
Source: PLoS One. 2021 May 19;16(5):e0251877. doi: 10.1371/journal.pone.0251877 (PMC8133481; doi:10.1371/journal.pone.0251877)
Supplement: S2 Table — (DOCX) [file pone.0251877.s006.docx]

**S2 Table.** Proportion of patients admitted experiencing an acute healthcare or critical care encounter per year stratified by age and sex

|  | **1995** | | **1996** | | **1997** | | **1998** | |
| --- | --- | --- | --- | --- | --- | --- | --- | --- |
|  | **Women (n=566,110)** | **Men (n=547203)** | **Women (n=563,218)** | **Men (n=544659)** | **Women (n=575,223)** | **Men (n=556622)** | **Women (n=581,738)** | **Men (n=564670)** |
| **ED Visits, n (%)** | | | | | | | | |
| Overall | N/A | N/A | N/A | N/A | N/A | N/A | N/A | N/A |
| 20 | N/A | N/A | N/A | N/A | N/A | N/A | N/A | N/A |
| 30 | N/A | N/A | N/A | N/A | N/A | N/A | N/A | N/A |
| 40 | N/A | N/A | N/A | N/A | N/A | N/A | N/A | N/A |
| 50 | N/A | N/A | N/A | N/A | N/A | N/A | N/A | N/A |
| 60 | N/A | N/A | N/A | N/A | N/A | N/A | N/A | N/A |
| 70 | N/A | N/A | N/A | N/A | N/A | N/A | N/A | N/A |
| 80 | N/A | N/A | N/A | N/A | N/A | N/A | N/A | N/A |
| 90 | N/A | N/A | N/A | N/A | N/A | N/A | N/A | N/A |
| 100 | N/A | N/A | N/A | N/A | N/A | N/A | N/A | N/A |
| **Hospital admissions, n (%)** | | | | | | | | |
| Overall | 47,521 (8.4%) | 26,898 (4.9%) | 44,176 (7.8%) | 25,415 (4.7%) | 42,715 (7.4%) | 24,590 (4.4%) | 41,390 (7.1%) | 24,339 (4.3%) |
| 20 | 6,089 (5.2%) | 1,957 (1.7%) | 5,753 (4.9%) | 1,758 (1.5%) | 5,344 (4.6%) | 1,613 (1.4%) | 5,207 (4.5%) | 1,645 (1.4%) |
| 30 | 15,474 (11.4%) | 2,994 (2.1%) | 13,791 (10.7%) | 2,548 (1.9%) | 12,670 (10.3%) | 2,122 (1.7%) | 11,874 (9.8%) | 1,828 (1.5%) |
| 40 | 5,346 (4.9%) | 3,273 (3.0%) | 5,056 (4.6%) | 3,017 (2.7%) | 4,936 (4.3%) | 2,872 (2.5%) | 5,003 (4.3%) | 2,968 (2.5%) |
| 50 | 3,888 (5.4%) | 3,491 (4.8%) | 3,446 (4.8%) | 3,228 (4.5%) | 4,241 (4.8%) | 3,548 (4.1%) | 4,246 (4.5%) | 3,817 (4.1%) |
| 60 | 3,869 (7.4%) | 4,288 (8.6%) | 3,822 (7.2%) | 4,262 (8.3%) | 3,600 (6.7%) | 4,081 (7.8%) | 3,530 (6.5%) | 3,966 (7.5%) |
| 70 | 5,839 (12.4%) | 5,930 (16.0%) | 5,618 (11.9%) | 5,901 (15.3%) | 5,260 (11.3%) | 5,632 (14.4%) | 5,097 (11.1%) | 5,630 (14.1%) |
| 80 | 5,155 (20.0%) | 4,074 (24.9%) | 4,748 (18.9%) | 3,847 (24.0%) | 4,716 (18.7%) | 3,841 (23.7%) | 4,468 (18.2%) | 3,589 (22.8%) |
| 90 | 1,799 (25.4%) | 876 (32.7%) | 1,871 (25.4%) | 838 (30.7%) | 1,902 (24.5%) | 864 (30.7%) | 1,894 (23.9%) | 878 (28.8%) |
| 100 | 62 (15.7%) | 15 (19.5%) | 71 (16.7%) | 16 (18.8%) | 46 (9.7%) | 17 (21.8%) | 71 (14.2%) | 18 (18.6%) |
| **ICU admissions, n (%)** | | | | | | | | |
| Overall | N/A | N/A | N/A | N/A | N/A | N/A | N/A | N/A |
| 20 | N/A | N/A | N/A | N/A | N/A | N/A | N/A | N/A |
| 30 | N/A | N/A | N/A | N/A | N/A | N/A | N/A | N/A |
| 40 | N/A | N/A | N/A | N/A | N/A | N/A | N/A | N/A |
| 50 | N/A | N/A | N/A | N/A | N/A | N/A | N/A | N/A |
| 60 | N/A | N/A | N/A | N/A | N/A | N/A | N/A | N/A |
| 70 | N/A | N/A | N/A | N/A | N/A | N/A | N/A | N/A |
| 80 | N/A | N/A | N/A | N/A | N/A | N/A | N/A | N/A |
| 90 | N/A | N/A | N/A | N/A | N/A | N/A | N/A | N/A |
| 100 | N/A | N/A | N/A | N/A | N/A | N/A | N/A | N/A |
| **Receipt of IMV, n (%)** | | | | | | | | |
| Overall | 888 (0.2%) | 1,322 (0.2%) | 895 (0.2%) | 1,330 (0.2%) | 891 (0.2%) | 1,401 (0.3%) | 882 (0.2%) | 1,372 (0.2%) |
| 20 | 27 (<0.1%) | 53 (<0.1%) | 34 (<0.1%) | 40 (<0.1%) | 31 (<0.1%) | 47 (<0.1%) | 21 (<0.1%) | 63 (0.1%) |
| 30 | 52 (<0.1%) | 57 (<0.1%) | 43 (<0.1%) | 73 (0.1%) | 52 (<0.1%) | 49 (<0.1%) | 36 (<0.1%) | 46 (<0.1%) |
| 40 | 58 (0.1%) | 85 (0.1%) | 66 (0.1%) | 78 (0.1%) | 59 (0.1%) | 96 (0.1%) | 67 (0.1%) | 75 (0.1%) |
| 50 | 83 (0.1%) | 152 (0.2%) | 73 (0.1%) | 142 (0.2%) | 100 (0.1%) | 178 (0.2%) | 104 (0.1%) | 195 (0.2%) |
| 60 | 166 (0.3%) | 275 (0.5%) | 156 (0.3%) | 293 (0.6%) | 152 (0.3%) | 311 (0.6%) | 132 (0.2%) | 284 (0.5%) |
| 70 | 287 (0.6%) | 449 (1.2%) | 287 (0.6%) | 459 (1.2%) | 303 (0.6%) | 483 (1.2%) | 302 (0.7%) | 467 (1.2%) |
| 80 | 185 (0.7%) | 232 (1.4%) | 196 (0.8%) | 221 (1.4%) | 163 (0.6%) | 213 (1.3%) | 184 (0.8%) | 220 (1.4%) |
| 90 | 29 (0.4%) | 19 (0.7%) | 39 (0.5%) | 24 (0.9%) | 31 (0.4%) | 24 (0.9%) | 36 (0.5%) | 22 (0.7%) |
| 100 | <=5 (0.3%) | 0 (<0.1%) | <=5 (0.2%) | 0 (<0.1%) | 0 (<0.1%) | 0 (<0.1%) | 0 (<0.1%) | 0 (<0.1%) |
| **Death, n (%)** | | | | | | | | |
| Overall | 3,819 (0.7%) | 4,077 (0.7%) | 3,730 (0.7%) | 4,009 (0.7%) | 3,831 (0.7%) | 3,900 (0.7%) | 3,834 (0.7%) | 3,819 (0.7%) |
| 20 | 14 (<0.1%) | 55 (<0.1%) | 19 (<0.1%) | 60 (0.1%) | 17 (<0.1%) | 50 (<0.1%) | 15 (<0.1%) | 61 (0.1%) |
| 30 | 36 (<0.1%) | 100 (0.1%) | 33 (<0.1%) | 87 (0.1%) | 43 (<0.1%) | 68 (0.1%) | 36 (<0.1%) | 69 (0.1%) |
| 40 | 94 (0.1%) | 181 (0.2%) | 86 (0.1%) | 151 (0.1%) | 81 (0.1%) | 139 (0.1%) | 87 (0.1%) | 143 (0.1%) |
| 50 | 158 (0.2%) | 255 (0.4%) | 147 (0.2%) | 262 (0.4%) | 181 (0.2%) | 233 (0.3%) | 213 (0.2%) | 282 (0.3%) |
| 60 | 337 (0.6%) | 493 (1.0%) | 331 (0.6%) | 511 (1.0%) | 308 (0.6%) | 535 (1.0%) | 317 (0.6%) | 477 (0.9%) |
| 70 | 789 (1.7%) | 1,141 (3.1%) | 760 (1.6%) | 1,201 (3.1%) | 747 (1.6%) | 1,068 (2.7%) | 740 (1.6%) | 1,116 (2.8%) |
| 80 | 1,271 (4.9%) | 1,326 (8.1%) | 1,197 (4.8%) | 1,211 (7.6%) | 1,176 (4.7%) | 1,306 (8.1%) | 1,171 (4.8%) | 1,135 (7.2%) |
| 90 | 1,031 (14.6%) | 503 (18.7%) | 1,046 (14.2%) | 509 (18.7%) | 1,164 (15.0%) | 473 (16.8%) | 1,119 (14.1%) | 514 (16.8%) |
| 100 | 89 (22.5%) | 23 (29.9%) | 111 (26.1%) | 17 (20.0%) | 114 (24.0%) | 28 (35.9%) | 136 (27.1%) | 22 (22.7%) |

|  | **1999** | | **2000** | | **2001** | | **2002** | |
| --- | --- | --- | --- | --- | --- | --- | --- | --- |
|  | **Women (n=586,825)** | **Men (n=569,145)** | **Women (n=597,476)** | **Men (n=577,044)** | **Women (n=618,967)** | **Men (n=598,309)** | **Women (n=617,918)** | **Men (n=597,884)** |
| **ED Visits, n (%)** | | | | | | | | |
| Overall | N/A | N/A | N/A | N/A | N/A | N/A | N/A | N/A |
| 20 | N/A | N/A | N/A | N/A | N/A | N/A | N/A | N/A |
| 30 | N/A | N/A | N/A | N/A | N/A | N/A | N/A | N/A |
| 40 | N/A | N/A | N/A | N/A | N/A | N/A | N/A | N/A |
| 50 | N/A | N/A | N/A | N/A | N/A | N/A | N/A | N/A |
| 60 | N/A | N/A | N/A | N/A | N/A | N/A | N/A | N/A |
| 70 | N/A | N/A | N/A | N/A | N/A | N/A | N/A | N/A |
| 80 | N/A | N/A | N/A | N/A | N/A | N/A | N/A | N/A |
| 90 | N/A | N/A | N/A | N/A | N/A | N/A | N/A | N/A |
| 100 | N/A | N/A | N/A | N/A | N/A | N/A | N/A | N/A |
| **Hospital admissions, n (%)** | | | | | | | | |
| Overall | 41,317 (7.0%) | 23,996 (4.2%) | 41,187 (6.9%) | 24,162 (4.2%) | 43,318 (7.0%) | 25,389 (4.2%) | 41,555 (6.7%) | 24,986 (4.2%) |
| 20 | 4,900 (4.3%) | 1,589 (1.4%) | 4,893 (4.2%) | 1,573 (1.4%) | 4,771 (4.0%) | 1,623 (1.4%) | 4,492 (3.8%) | 1,610 (1.4%) |
| 30 | 11,897 (9.7%) | 1,842 (1.5%) | 11,697 (9.5%) | 1,793 (1.4%) | 12,354 (9.9%) | 1,792 (1.4%) | 11,689 (9.5%) | 1,715 (1.4%) |
| 40 | 5,010 (4.2%) | 2,740 (2.2%) | 5,002 (4.0%) | 2,863 (2.3%) | 5,216 (4.1%) | 2,915 (2.2%) | 5,091 (4.0%) | 2,919 (2.2%) |
| 50 | 3,908 (4.3%) | 3,531 (3.9%) | 4,099 (4.4%) | 3,548 (3.9%) | 4,080 (4.4%) | 3,509 (3.8%) | 3,804 (4.0%) | 3,525 (3.7%) |
| 60 | 3,670 (6.4%) | 4,061 (7.3%) | 3,543 (6.2%) | 3,943 (7.1%) | 3,748 (6.2%) | 4,154 (7.2%) | 3,544 (5.8%) | 4,225 (7.1%) |
| 70 | 5,021 (10.8%) | 5,677 (14.1%) | 4,856 (10.5%) | 5,554 (13.7%) | 5,016 (10.3%) | 5,772 (13.2%) | 4,820 (10.2%) | 5,344 (12.5%) |
| 80 | 4,805 (18.5%) | 3,628 (22.2%) | 4,960 (18.0%) | 3,893 (21.9%) | 5,872 (17.8%) | 4,508 (21.0%) | 5,864 (17.4%) | 4,577 (20.6%) |
| 90 | 2,036 (24.1%) | 912 (27.5%) | 2,059 (24.3%) | 966 (28.1%) | 2,179 (23.7%) | 1,096 (28.1%) | 2,176 (23.7%) | 1,046 (27.3%) |
| 100 | 70 (14.1%) | 16 (17.6%) | 78 (15.0%) | 29 (18.1%) | 82 (12.9%) | 20 (14.3%) | 75 (11.1%) | 25 (13.0%) |
| **ICU admissions, n (%)** | | | | | | | | |
| Overall | N/A | N/A | N/A | N/A | N/A | N/A | N/A | N/A |
| 20 | N/A | N/A | N/A | N/A | N/A | N/A | N/A | N/A |
| 30 | N/A | N/A | N/A | N/A | N/A | N/A | N/A | N/A |
| 40 | N/A | N/A | N/A | N/A | N/A | N/A | N/A | N/A |
| 50 | N/A | N/A | N/A | N/A | N/A | N/A | N/A | N/A |
| 60 | N/A | N/A | N/A | N/A | N/A | N/A | N/A | N/A |
| 70 | N/A | N/A | N/A | N/A | N/A | N/A | N/A | N/A |
| 80 | N/A | N/A | N/A | N/A | N/A | N/A | N/A | N/A |
| 90 | N/A | N/A | N/A | N/A | N/A | N/A | N/A | N/A |
| 100 | N/A | N/A | N/A | N/A | N/A | N/A | N/A | N/A |
| **Receipt of IMV, n (%)** | | | | | | | | |
| Overall | 915 (0.2%) | 1,377 (0.2%) | 917 (0.2%) | 1,402 (0.2%) | 983 (0.2%) | 1,430 (0.2%) | 1,000 (0.2%) | 1,453 (0.2%) |
| 20 | 25 (<0.1%) | 46 (<0.1%) | 31 (<0.1%) | 52 (<0.1%) | 25 (<0.1%) | 44 (<0.1%) | 43 (<0.1%) | 53 (<0.1%) |
| 30 | 49 (<0.1%) | 46 (<0.1%) | 43 (<0.1%) | 55 (<0.1%) | 45 (<0.1%) | 44 (<0.1%) | 49 (<0.1%) | 47 (<0.1%) |
| 40 | 60 (0.1%) | 98 (0.1%) | 71 (0.1%) | 88 (0.1%) | 65 (0.1%) | 100 (0.1%) | 76 (0.1%) | 102 (0.1%) |
| 50 | 114 (0.1%) | 174 (0.2%) | 119 (0.1%) | 187 (0.2%) | 90 (0.1%) | 183 (0.2%) | 115 (0.1%) | 208 (0.2%) |
| 60 | 152 (0.3%) | 277 (0.5%) | 156 (0.3%) | 292 (0.5%) | 185 (0.3%) | 304 (0.5%) | 167 (0.3%) | 297 (0.5%) |
| 70 | 289 (0.6%) | 471 (1.2%) | 237 (0.5%) | 435 (1.1%) | 288 (0.6%) | 464 (1.1%) | 281 (0.6%) | 431 (1.0%) |
| 80 | 188 (0.7%) | 243 (1.5%) | 216 (0.8%) | 257 (1.4%) | 248 (0.8%) | 264 (1.2%) | 225 (0.7%) | 290 (1.3%) |
| 90 | 37 (0.4%) | 21 (0.6%) | 44 (0.5%) | 36 (1.0%) | 37 (0.4%) | 26 (0.7%) | 44 (0.5%) | 25 (0.7%) |
| 100 | <=5 (0.2%) | <=5 (1.1%) | 0 (<0.1%) | 0 (<0.1%) | 0 (<0.1%) | <=5 (0.7%) | 0 (<0.1%) | 0 (<0.1%) |
| **Death, n (%)** | | | | | | | | |
| Overall | 3,854 (0.7%) | 3,959 (0.7%) | 3,826 (0.6%) | 3,829 (0.7%) | 4,080 (0.7%) | 4,129 (0.7%) | 3,861 (0.6%) | 4,019 (0.7%) |
| 20 | 11 (<0.1%) | 53 (<0.1%) | 24 (<0.1%) | 55 (<0.1%) | 25 (<0.1%) | 50 (<0.1%) | 25 (<0.1%) | 60 (0.1%) |
| 30 | 29 (<0.1%) | 70 (0.1%) | 26 (<0.1%) | 64 (0.1%) | 40 (<0.1%) | 65 (0.1%) | 32 (<0.1%) | 58 (<0.1%) |
| 40 | 97 (0.1%) | 158 (0.1%) | 76 (0.1%) | 123 (0.1%) | 73 (0.1%) | 135 (0.1%) | 93 (0.1%) | 138 (0.1%) |
| 50 | 189 (0.2%) | 305 (0.3%) | 201 (0.2%) | 242 (0.3%) | 176 (0.2%) | 307 (0.3%) | 181 (0.2%) | 306 (0.3%) |
| 60 | 326 (0.6%) | 520 (0.9%) | 297 (0.5%) | 452 (0.8%) | 305 (0.5%) | 498 (0.9%) | 298 (0.5%) | 500 (0.8%) |
| 70 | 698 (1.5%) | 1,097 (2.7%) | 663 (1.4%) | 1,033 (2.5%) | 707 (1.4%) | 1,029 (2.3%) | 656 (1.4%) | 1,010 (2.4%) |
| 80 | 1,225 (4.7%) | 1,189 (7.3%) | 1,249 (4.5%) | 1,242 (7.0%) | 1,435 (4.4%) | 1,401 (6.5%) | 1,299 (3.8%) | 1,360 (6.1%) |
| 90 | 1,147 (13.6%) | 546 (16.5%) | 1,173 (13.8%) | 583 (17.0%) | 1,178 (12.8%) | 617 (15.8%) | 1,144 (12.4%) | 567 (14.8%) |
| 100 | 132 (26.6%) | 21 (23.1%) | 117 (22.5%) | 35 (21.9%) | 141 (22.2%) | 27 (19.3%) | 133 (19.8%) | 20 (10.4%) |

|  | **2003** | | **2004** | | **2005** | | **2006** | |
| --- | --- | --- | --- | --- | --- | --- | --- | --- |
|  | **Women (n=634,582)** | **Men (n=616,155)** | **Women (n=639,278)** | **Men (n=621,005)** | **Women (n=647,638)** | **Men (n=628,855)** | **Women (n=643,946)** | **Men (n=625,884)** |
| **ED Visits, n (%)** | | | | | | | | |
| Overall | 105,918 (16.7%) | 99,556 (16.2%) | 111,016 (17.4%) | 102,591 (16.5%) | 116,019 (17.9%) | 106,379 (16.9%) | 116,070 (18.0%) | 106,691 (17.0%) |
| 20 | 18,946 (15.5%) | 19,243 (15.8%) | 19,602 (16.0%) | 19,605 (16.0%) | 20,611 (16.8%) | 20,501 (16.6%) | 20,701 (16.9%) | 20,614 (16.8%) |
| 30 | 17,434 (14.5%) | 16,306 (13.4%) | 17,816 (15.2%) | 15,797 (13.5%) | 18,479 (15.8%) | 16,322 (14.0%) | 19,057 (16.4%) | 16,430 (14.2%) |
| 40 | 18,980 (14.3%) | 20,094 (14.5%) | 19,801 (14.7%) | 20,871 (14.8%) | 20,412 (15.2%) | 21,097 (15.0%) | 19,469 (15.2%) | 19,850 (14.8%) |
| 50 | 15,142 (15.1%) | 14,780 (14.9%) | 16,436 (16.0%) | 15,638 (15.4%) | 17,419 (16.2%) | 16,761 (15.7%) | 18,003 (16.5%) | 17,165 (15.6%) |
| 60 | 11,045 (16.9%) | 10,798 (16.8%) | 11,994 (17.6%) | 11,676 (17.5%) | 12,501 (18.0%) | 12,111 (17.7%) | 12,262 (17.5%) | 12,169 (17.9%) |
| 70 | 10,106 (21.0%) | 9,374 (21.7%) | 10,243 (21.8%) | 9,710 (22.6%) | 10,537 (22.0%) | 9,766 (22.6%) | 10,656 (21.8%) | 10,028 (22.7%) |
| 80 | 10,439 (30.2%) | 7,244 (31.9%) | 10,936 (31.1%) | 7,516 (32.0%) | 11,446 (31.5%) | 7,856 (32.5%) | 11,425 (31.1%) | 8,296 (32.3%) |
| 90 | 3,684 (36.4%) | 1,688 (38.3%) | 4,029 (37.8%) | 1,745 (37.8%) | 4,442 (38.4%) | 1,925 (37.2%) | 4,292 (37.8%) | 2,099 (39.9%) |
| 100 | 142 (18.7%) | 29 (14.7%) | 159 (18.5%) | 33 (12.5%) | 172 (19.6%) | 40 (13.6%) | 205 (21.0%) | 40 (10.8%) |
| **Hospital admissions, n (%)** | | | | | | | | |
| Overall | 41,526 (6.5%) | 24,828 (4.0%) | 42,027 (6.6%) | 24,796 (4.0%) | 42,700 (6.6%) | 25,385 (4.0%) | 41,157 (6.4%) | 24,309 (3.9%) |
| 20 | 4,454 (3.6%) | 1,558 (1.3%) | 4,359 (3.6%) | 1,637 (1.3%) | 4,413 (3.6%) | 1,688 (1.4%) | 4,324 (3.5%) | 1,478 (1.2%) |
| 30 | 11,512 (9.6%) | 1,588 (1.3%) | 11,640 (10.0%) | 1,417 (1.2%) | 11,897 (10.2%) | 1,518 (1.3%) | 11,716 (10.1%) | 1,291 (1.1%) |
| 40 | 5,220 (3.9%) | 2,751 (2.0%) | 5,255 (3.9%) | 2,763 (2.0%) | 5,279 (3.9%) | 2,737 (1.9%) | 4,773 (3.7%) | 2,391 (1.8%) |
| 50 | 3,855 (3.9%) | 3,524 (3.6%) | 4,009 (3.9%) | 3,585 (3.5%) | 3,903 (3.6%) | 3,673 (3.4%) | 3,744 (3.4%) | 3,556 (3.2%) |
| 60 | 3,792 (5.8%) | 4,363 (6.8%) | 3,794 (5.6%) | 4,397 (6.6%) | 3,862 (5.6%) | 4,575 (6.7%) | 3,595 (5.1%) | 4,352 (6.4%) |
| 70 | 4,783 (9.9%) | 5,211 (12.1%) | 4,666 (9.9%) | 5,119 (11.9%) | 4,664 (9.8%) | 5,144 (11.9%) | 4,551 (9.3%) | 5,059 (11.4%) |
| 80 | 5,571 (16.1%) | 4,649 (20.4%) | 5,821 (16.6%) | 4,690 (20.0%) | 5,909 (16.3%) | 4,738 (19.6%) | 5,825 (15.9%) | 4,828 (18.8%) |
| 90 | 2,266 (22.4%) | 1,164 (26.4%) | 2,392 (22.4%) | 1,163 (25.2%) | 2,668 (23.1%) | 1,285 (24.9%) | 2,509 (22.1%) | 1,326 (25.2%) |
| 100 | 73 (9.6%) | 20 (10.2%) | 91 (10.6%) | 25 (9.4%) | 105 (11.9%) | 27 (9.2%) | 120 (12.3%) | 28 (7.6%) |
| **ICU admissions, n (%)** | | | | | | | | |
| Overall | 2,606 (0.4%) | 3,413 (0.6%) | 2,559 (0.4%) | 3,333 (0.5%) | 2,590 (0.4%) | 3,323 (0.5%) | 2,456 (0.4%) | 3,310 (0.5%) |
| 20 | 86 (0.1%) | 121 (0.1%) | 105 (0.1%) | 120 (0.1%) | 92 (0.1%) | 119 (0.1%) | 96 (0.1%) | 136 (0.1%) |
| 30 | 101 (0.1%) | 111 (0.1%) | 98 (0.1%) | 109 (0.1%) | 112 (0.1%) | 109 (0.1%) | 106 (0.1%) | 113 (0.1%) |
| 40 | 199 (0.1%) | 253 (0.2%) | 204 (0.2%) | 237 (0.2%) | 218 (0.2%) | 258 (0.2%) | 191 (0.1%) | 234 (0.2%) |
| 50 | 299 (0.3%) | 518 (0.5%) | 296 (0.3%) | 461 (0.5%) | 303 (0.3%) | 455 (0.4%) | 295 (0.3%) | 463 (0.4%) |
| 60 | 411 (0.6%) | 700 (1.1%) | 382 (0.6%) | 677 (1.0%) | 388 (0.6%) | 685 (1.0%) | 356 (0.5%) | 664 (1.0%) |
| 70 | 666 (1.4%) | 851 (2.0%) | 611 (1.3%) | 885 (2.1%) | 593 (1.2%) | 816 (1.9%) | 526 (1.1%) | 803 (1.8%) |
| 80 | 681 (2.0%) | 733 (3.2%) | 680 (1.9%) | 722 (3.1%) | 687 (1.9%) | 746 (3.1%) | 710 (1.9%) | 762 (3.0%) |
| 90 | 161 (1.6%) | 126 (2.9%) | 176 (1.6%) | 121 (2.6%) | 193 (1.7%) | 134 (2.6%) | 172 (1.5%) | 135 (2.6%) |
| 100 | <=5 (0.3%) | 0 (<0.1%) | 7 (0.8%) | <=5 (0.4%) | <=5 (0.5%) | <=5 (0.3%) | <=5 (0.4%) | 0 (<0.1%) |
| **Receipt of IMV, n (%)** | | | | | | | | |
| Overall | 985 (0.2%) | 1,479 (0.2%) | 985 (0.2%) | 1,501 (0.2%) | 1,072 (0.2%) | 1,579 (0.3%) | 1,084 (0.2%) | 1,622 (0.3%) |
| 20 | 22 (<0.1%) | 50 (<0.1%) | 33 (<0.1%) | 51 (<0.1%) | 39 (<0.1%) | 52 (<0.1%) | 42 (<0.1%) | 65 (0.1%) |
| 30 | 37 (<0.1%) | 39 (<0.1%) | 29 (<0.1%) | 48 (<0.1%) | 48 (<0.1%) | 45 (<0.1%) | 35 (<0.1%) | 56 (<0.1%) |
| 40 | 70 (0.1%) | 106 (0.1%) | 74 (0.1%) | 95 (0.1%) | 87 (0.1%) | 112 (0.1%) | 84 (0.1%) | 120 (0.1%) |
| 50 | 125 (0.1%) | 217 (0.2%) | 121 (0.1%) | 199 (0.2%) | 142 (0.1%) | 213 (0.2%) | 164 (0.1%) | 239 (0.2%) |
| 60 | 176 (0.3%) | 333 (0.5%) | 189 (0.3%) | 375 (0.6%) | 185 (0.3%) | 365 (0.5%) | 162 (0.2%) | 337 (0.5%) |
| 70 | 278 (0.6%) | 397 (0.9%) | 261 (0.6%) | 421 (1.0%) | 258 (0.5%) | 438 (1.0%) | 275 (0.6%) | 432 (1.0%) |
| 80 | 228 (0.7%) | 307 (1.3%) | 226 (0.6%) | 288 (1.2%) | 261 (0.7%) | 329 (1.4%) | 269 (0.7%) | 338 (1.3%) |
| 90 | 49 (0.5%) | 30 (0.7%) | 50 (0.5%) | 23 (0.5%) | 51 (0.4%) | 25 (0.5%) | 52 (0.5%) | 35 (0.7%) |
| 100 | 0 (<0.1%) | 0 (<0.1%) | <=5 (0.2%) | <=5 (0.4%) | <=5 (0.1%) | 0 (<0.1%) | <=5 (0.1%) | 0 (<0.1%) |
| **Death, n (%)** | | | | | | | | |
| Overall | 4,203 (0.7%) | 4,271 (0.7%) | 4,252 (0.7%) | 4,083 (0.7%) | 4,387 (0.7%) | 4,008 (0.6%) | 4,119 (0.6%) | 4,261 (0.7%) |
| 20 | 20 (<0.1%) | 71 (0.1%) | 24 (<0.1%) | 49 (<0.1%) | 18 (<0.1%) | 61 (<0.1%) | 24 (<0.1%) | 58 (<0.1%) |
| 30 | 29 (<0.1%) | 73 (0.1%) | 34 (<0.1%) | 54 (<0.1%) | 32 (<0.1%) | 70 (0.1%) | 24 (<0.1%) | 59 (0.1%) |
| 40 | 88 (0.1%) | 155 (0.1%) | 90 (0.1%) | 141 (0.1%) | 96 (0.1%) | 130 (0.1%) | 82 (0.1%) | 152 (0.1%) |
| 50 | 204 (0.2%) | 296 (0.3%) | 190 (0.2%) | 295 (0.3%) | 199 (0.2%) | 296 (0.3%) | 220 (0.2%) | 332 (0.3%) |
| 60 | 371 (0.6%) | 524 (0.8%) | 372 (0.5%) | 527 (0.8%) | 340 (0.5%) | 473 (0.7%) | 346 (0.5%) | 519 (0.8%) |
| 70 | 705 (1.5%) | 1,017 (2.4%) | 654 (1.4%) | 908 (2.1%) | 659 (1.4%) | 866 (2.0%) | 616 (1.3%) | 886 (2.0%) |
| 80 | 1,406 (4.1%) | 1,435 (6.3%) | 1,372 (3.9%) | 1,442 (6.1%) | 1,460 (4.0%) | 1,378 (5.7%) | 1,340 (3.7%) | 1,446 (5.6%) |
| 90 | 1,248 (12.3%) | 679 (15.4%) | 1,359 (12.7%) | 641 (13.9%) | 1,423 (12.3%) | 704 (13.6%) | 1,301 (11.5%) | 773 (14.7%) |
| 100 | 132 (17.4%) | 21 (10.7%) | 157 (18.3%) | 26 (9.8%) | 160 (18.2%) | 30 (10.2%) | 166 (17.0%) | 36 (9.8%) |

|  | **2007** | | **2008** | | **2009** | | **2010** | |
| --- | --- | --- | --- | --- | --- | --- | --- | --- |
|  | **Women (n=653,857)** | **Men (n=635,870)** | **Women (n=659,311)** | **Men (n=642,643)** | **Women (n=663,245)** | **Men (n=646,611)** | **Women (n=674,816)** | **Men (n=655,778)** |
| **ED Visits, n (%)** | | | | | | | | |
| Overall | 118,093 (18.1%) | 107,677 (16.9%) | 119,598 (18.1%) | 107,698 (16.8%) | 122,100 (18.4%) | 108,296 (16.7%) | 126,347 (18.7%) | 110,910 (16.9%) |
| 20 | 20,904 (17.2%) | 20,480 (16.8%) | 21,011 (17.5%) | 19,859 (16.4%) | 21,825 (18.1%) | 20,299 (16.6%) | 22,129 (18.1%) | 21,142 (17.0%) |
| 30 | 18,555 (16.2%) | 15,874 (14.0%) | 18,523 (16.3%) | 15,779 (14.0%) | 19,005 (16.8%) | 15,666 (14.0%) | 19,661 (17.1%) | 16,086 (14.3%) |
| 40 | 18,253 (14.9%) | 18,201 (14.4%) | 17,659 (14.8%) | 17,191 (14.0%) | 18,056 (14.8%) | 17,214 (13.9%) | 18,771 (15.3%) | 17,326 (14.0%) |
| 50 | 18,588 (16.5%) | 17,773 (15.6%) | 19,088 (16.5%) | 18,414 (15.6%) | 19,668 (16.8%) | 18,869 (15.7%) | 20,805 (17.1%) | 19,270 (15.6%) |
| 60 | 15,009 (17.8%) | 14,624 (17.7%) | 16,517 (18.2%) | 15,622 (17.6%) | 15,545 (17.7%) | 14,886 (17.3%) | 16,301 (18.2%) | 15,118 (17.4%) |
| 70 | 10,978 (22.2%) | 10,114 (22.3%) | 10,999 (21.9%) | 9,989 (21.8%) | 11,706 (22.3%) | 10,413 (21.6%) | 11,788 (22.2%) | 10,652 (22.0%) |
| 80 | 11,289 (30.9%) | 8,452 (32.2%) | 11,189 (30.7%) | 8,694 (31.9%) | 11,400 (30.9%) | 8,758 (31.4%) | 11,530 (31.3%) | 8,858 (31.2%) |
| 90 | 4,316 (37.4%) | 2,113 (38.8%) | 4,412 (37.8%) | 2,091 (37.5%) | 4,666 (38.0%) | 2,137 (36.7%) | 5,129 (38.3%) | 2,402 (37.1%) |
| 100 | 201 (18.0%) | 46 (10.2%) | 200 (17.1%) | 59 (13.1%) | 229 (16.4%) | 54 (9.6%) | 233 (16.4%) | 56 (9.0%) |
| **Hospital admissions, n (%)** | | | | | | | | |
| Overall | 40,782 (6.2%) | 24,669 (3.9%) | 40,529 (6.1%) | 24,263 (3.8%) | 40,617 (6.1%) | 24,344 (3.8%) | 40,622 (6.0%) | 23,973 (3.7%) |
| 20 | 4,181 (3.4%) | 1,368 (1.1%) | 4,101 (3.4%) | 1,273 (1.0%) | 4,021 (3.3%) | 1,248 (1.0%) | 3,864 (3.2%) | 1,318 (1.1%) |
| 30 | 11,536 (10.1%) | 1,271 (1.1%) | 11,357 (10.0%) | 1,179 (1.0%) | 11,318 (10.0%) | 1,148 (1.0%) | 11,412 (9.9%) | 1,174 (1.0%) |
| 40 | 4,272 (3.5%) | 2,173 (1.7%) | 4,205 (3.5%) | 1,918 (1.6%) | 4,313 (3.5%) | 1,940 (1.6%) | 4,396 (3.6%) | 1,773 (1.4%) |
| 50 | 3,725 (3.3%) | 3,513 (3.1%) | 3,754 (3.2%) | 3,541 (3.0%) | 3,831 (3.3%) | 3,692 (3.1%) | 3,855 (3.2%) | 3,607 (2.9%) |
| 60 | 4,300 (5.1%) | 5,115 (6.2%) | 4,599 (5.1%) | 5,244 (5.9%) | 4,319 (4.9%) | 5,064 (5.9%) | 4,340 (4.9%) | 4,908 (5.7%) |
| 70 | 4,506 (9.1%) | 5,071 (11.2%) | 4,387 (8.7%) | 4,866 (10.6%) | 4,638 (8.8%) | 4,991 (10.4%) | 4,493 (8.5%) | 4,856 (10.0%) |
| 80 | 5,697 (15.6%) | 4,826 (18.4%) | 5,563 (15.2%) | 4,883 (17.9%) | 5,430 (14.7%) | 4,896 (17.5%) | 5,299 (14.4%) | 4,827 (17.0%) |
| 90 | 2,448 (21.2%) | 1,300 (23.9%) | 2,448 (21.0%) | 1,329 (23.9%) | 2,609 (21.2%) | 1,330 (22.8%) | 2,826 (21.1%) | 1,468 (22.7%) |
| 100 | 117 (10.5%) | 32 (7.1%) | 115 (9.8%) | 30 (6.7%) | 138 (9.9%) | 35 (6.2%) | 137 (9.6%) | 42 (6.8%) |
| **ICU admissions, n (%)** | | | | | | | | |
| Overall | 2,452 (0.4%) | 3,295 (0.5%) | 2,411 (0.4%) | 3,247 (0.5%) | 2,486 (0.4%) | 3,263 (0.5%) | 2,549 (0.4%) | 3,203 (0.5%) |
| 20 | 85 (0.1%) | 127 (0.1%) | 84 (0.1%) | 114 (0.1%) | 80 (0.1%) | 114 (0.1%) | 94 (0.1%) | 135 (0.1%) |
| 30 | 124 (0.1%) | 111 (0.1%) | 80 (0.1%) | 88 (0.1%) | 117 (0.1%) | 109 (0.1%) | 134 (0.1%) | 121 (0.1%) |
| 40 | 147 (0.1%) | 204 (0.2%) | 141 (0.1%) | 198 (0.2%) | 187 (0.2%) | 213 (0.2%) | 171 (0.1%) | 176 (0.1%) |
| 50 | 274 (0.2%) | 462 (0.4%) | 319 (0.3%) | 444 (0.4%) | 323 (0.3%) | 486 (0.4%) | 326 (0.3%) | 490 (0.4%) |
| 60 | 450 (0.5%) | 742 (0.9%) | 460 (0.5%) | 745 (0.8%) | 460 (0.5%) | 707 (0.8%) | 439 (0.5%) | 697 (0.8%) |
| 70 | 528 (1.1%) | 796 (1.8%) | 523 (1.0%) | 802 (1.8%) | 519 (1.0%) | 802 (1.7%) | 573 (1.1%) | 761 (1.6%) |
| 80 | 695 (1.9%) | 748 (2.8%) | 647 (1.8%) | 754 (2.8%) | 623 (1.7%) | 721 (2.6%) | 604 (1.6%) | 684 (2.4%) |
| 90 | 145 (1.3%) | 103 (1.9%) | 152 (1.3%) | 100 (1.8%) | 171 (1.4%) | 110 (1.9%) | 202 (1.5%) | 135 (2.1%) |
| 100 | <=5 (0.4%) | <=5 (0.4%) | <=5 (0.4%) | <=5 (0.4%) | 6 (0.4%) | <=5 (0.2%) | 6 (0.4%) | <=5 (0.6%) |
| **Receipt of IMV, n (%)** | | | | | | | | |
| Overall | 1,076 (0.2%) | 1,722 (0.3%) | 1,140 (0.2%) | 1,725 (0.3%) | 1,192 (0.2%) | 1,815 (0.3%) | 1,162 (0.2%) | 1,796 (0.3%) |
| 20 | 28 (<0.1%) | 59 (<0.1%) | 33 (<0.1%) | 52 (<0.1%) | 40 (<0.1%) | 53 (<0.1%) | 28 (<0.1%) | 79 (0.1%) |
| 30 | 43 (<0.1%) | 61 (0.1%) | 29 (<0.1%) | 43 (<0.1%) | 52 (<0.1%) | 52 (<0.1%) | 42 (<0.1%) | 57 (0.1%) |
| 40 | 57 (<0.1%) | 101 (0.1%) | 65 (0.1%) | 101 (0.1%) | 90 (0.1%) | 99 (0.1%) | 77 (0.1%) | 92 (0.1%) |
| 50 | 126 (0.1%) | 233 (0.2%) | 140 (0.1%) | 243 (0.2%) | 150 (0.1%) | 285 (0.2%) | 166 (0.1%) | 248 (0.2%) |
| 60 | 217 (0.3%) | 438 (0.5%) | 247 (0.3%) | 435 (0.5%) | 231 (0.3%) | 479 (0.6%) | 228 (0.3%) | 452 (0.5%) |
| 70 | 253 (0.5%) | 449 (1.0%) | 264 (0.5%) | 426 (0.9%) | 282 (0.5%) | 465 (1.0%) | 296 (0.6%) | 460 (0.9%) |
| 80 | 311 (0.9%) | 341 (1.3%) | 316 (0.9%) | 393 (1.4%) | 284 (0.8%) | 349 (1.3%) | 277 (0.8%) | 347 (1.2%) |
| 90 | 39 (0.3%) | 40 (0.7%) | 45 (0.4%) | 31 (0.6%) | 63 (0.5%) | 33 (0.6%) | 46 (0.3%) | 58 (0.9%) |
| 100 | <=5 (0.2%) | 0 (<0.1%) | <=5 (0.1%) | <=5 (0.2%) | 0 (<0.1%) | 0 (<0.1%) | <=5 (0.1%) | <=5 (0.5%) |
| **Death, n (%)** | | | | | | | | |
| Overall | 4,219 (0.6%) | 4,304 (0.7%) | 4,290 (0.7%) | 4,315 (0.7%) | 4,162 (0.6%) | 4,375 (0.7%) | 4,288 (0.6%) | 4,216 (0.6%) |
| 20 | 13 (<0.1%) | 63 (0.1%) | 29 (<0.1%) | 44 (<0.1%) | 14 (<0.1%) | 53 (<0.1%) | 21 (<0.1%) | 41 (<0.1%) |
| 30 | 20 (<0.1%) | 66 (0.1%) | 32 (<0.1%) | 53 (<0.1%) | 22 (<0.1%) | 51 (<0.1%) | 29 (<0.1%) | 68 (0.1%) |
| 40 | 86 (0.1%) | 141 (0.1%) | 76 (0.1%) | 120 (0.1%) | 84 (0.1%) | 129 (0.1%) | 91 (0.1%) | 112 (0.1%) |
| 50 | 200 (0.2%) | 337 (0.3%) | 231 (0.2%) | 372 (0.3%) | 224 (0.2%) | 335 (0.3%) | 234 (0.2%) | 321 (0.3%) |
| 60 | 398 (0.5%) | 608 (0.7%) | 431 (0.5%) | 626 (0.7%) | 364 (0.4%) | 607 (0.7%) | 386 (0.4%) | 596 (0.7%) |
| 70 | 637 (1.3%) | 883 (1.9%) | 593 (1.2%) | 815 (1.8%) | 619 (1.2%) | 871 (1.8%) | 616 (1.2%) | 819 (1.7%) |
| 80 | 1,331 (3.6%) | 1,414 (5.4%) | 1,327 (3.6%) | 1,467 (5.4%) | 1,314 (3.6%) | 1,485 (5.3%) | 1,218 (3.3%) | 1,405 (4.9%) |
| 90 | 1,335 (11.6%) | 746 (13.7%) | 1,388 (11.9%) | 779 (14.0%) | 1,332 (10.8%) | 795 (13.7%) | 1,458 (10.9%) | 811 (12.5%) |
| 100 | 199 (17.8%) | 46 (10.2%) | 183 (15.6%) | 39 (8.6%) | 189 (13.5%) | 49 (8.7%) | 235 (16.5%) | 43 (6.9%) |

|  | **2011** | | **2012** | | **2013** | | **2014** | |
| --- | --- | --- | --- | --- | --- | --- | --- | --- |
|  | **Women (n=691,540)** | **Men (n=674,656)** | **Women (n=693,164)** | **Men (n=677,190)** | **Women (n=701,479)** | **Men (n=686,952)** | **Women (n=697,365)** | **Men (n=682,774)** |
| **ED Visits, n (%)** | | | | | | | | |
| Overall | 131,719 (19.0%) | 115,554 (17.1%) | 134,940 (19.5%) | 117,799 (17.4%) | 137,629 (19.6%) | 119,241 (17.4%) | 140,703 (20.2%) | 121,277 (17.8%) |
| 20 | 23,118 (18.9%) | 21,622 (17.2%) | 23,189 (18.6%) | 21,422 (16.6%) | 22,889 (19.4%) | 21,022 (17.2%) | 23,155 (21.3%) | 20,547 (18.3%) |
| 30 | 20,379 (17.4%) | 16,730 (14.5%) | 20,913 (17.9%) | 17,103 (14.8%) | 21,621 (18.1%) | 17,506 (14.7%) | 22,028 (18.4%) | 17,919 (15.0%) |
| 40 | 19,280 (15.6%) | 17,766 (14.2%) | 19,608 (16.2%) | 17,779 (14.6%) | 18,947 (16.0%) | 17,036 (14.3%) | 19,142 (16.6%) | 16,704 (14.5%) |
| 50 | 21,614 (17.1%) | 20,422 (15.8%) | 22,294 (17.7%) | 20,851 (16.1%) | 22,798 (17.5%) | 21,309 (15.8%) | 23,430 (17.6%) | 21,964 (15.9%) |
| 60 | 16,267 (18.1%) | 15,303 (17.4%) | 17,102 (18.7%) | 15,948 (17.8%) | 18,118 (18.8%) | 16,829 (17.9%) | 18,905 (19.0%) | 17,457 (18.1%) |
| 70 | 12,455 (22.4%) | 10,991 (21.6%) | 12,963 (23.1%) | 11,616 (22.3%) | 13,728 (22.7%) | 12,498 (22.0%) | 14,406 (22.8%) | 13,273 (22.4%) |
| 80 | 12,170 (31.1%) | 9,577 (30.7%) | 12,172 (31.9%) | 9,673 (31.6%) | 12,369 (31.7%) | 9,616 (30.7%) | 12,164 (31.8%) | 9,691 (30.8%) |
| 90 | 6,199 (38.1%) | 3,085 (37.4%) | 6,431 (38.0%) | 3,331 (37.8%) | 6,853 (38.7%) | 3,331 (37.1%) | 7,090 (38.8%) | 3,626 (37.5%) |
| 100 | 237 (13.8%) | 58 (7.1%) | 268 (15.7%) | 76 (9.1%) | 306 (15.0%) | 94 (9.3%) | 383 (17.6%) | 96 (9.0%) |
| **Hospital admissions, n (%)** | | | | | | | | |
| Overall | 41,938 (6.1%) | 25,441 (3.8%) | 42,229 (6.1%) | 25,533 (3.8%) | 42,707 (6.1%) | 26,145 (3.8%) | 42,977 (6.2%) | 26,332 (3.9%) |
| 20 | 3,914 (3.2%) | 1,334 (1.1%) | 3,686 (2.9%) | 1,300 (1.0%) | 3,444 (2.9%) | 1,235 (1.0%) | 3,394 (3.1%) | 1,109 (1.0%) |
| 30 | 11,597 (9.9%) | 1,140 (1.0%) | 11,691 (10.0%) | 1,228 (1.1%) | 11,976 (10.0%) | 1,234 (1.0%) | 11,939 (10.0%) | 1,246 (1.0%) |
| 40 | 4,515 (3.7%) | 1,859 (1.5%) | 4,465 (3.7%) | 1,891 (1.6%) | 4,180 (3.5%) | 1,723 (1.4%) | 4,213 (3.7%) | 1,710 (1.5%) |
| 50 | 3,823 (3.0%) | 3,749 (2.9%) | 3,969 (3.2%) | 3,667 (2.8%) | 3,878 (3.0%) | 3,790 (2.8%) | 3,963 (3.0%) | 3,740 (2.7%) |
| 60 | 4,286 (4.8%) | 5,067 (5.8%) | 4,367 (4.8%) | 4,994 (5.6%) | 4,578 (4.7%) | 5,323 (5.7%) | 4,618 (4.6%) | 5,328 (5.5%) |
| 70 | 4,537 (8.1%) | 5,048 (9.9%) | 4,796 (8.5%) | 5,262 (10.1%) | 5,097 (8.4%) | 5,598 (9.9%) | 5,311 (8.4%) | 5,888 (9.9%) |
| 80 | 5,618 (14.4%) | 5,287 (17.0%) | 5,525 (14.5%) | 5,075 (16.6%) | 5,599 (14.3%) | 5,126 (16.4%) | 5,421 (14.2%) | 5,033 (16.0%) |
| 90 | 3,522 (21.7%) | 1,918 (23.3%) | 3,586 (21.2%) | 2,069 (23.5%) | 3,771 (21.3%) | 2,051 (22.9%) | 3,881 (21.2%) | 2,213 (22.9%) |
| 100 | 126 (7.3%) | 39 (4.7%) | 144 (8.5%) | 47 (5.6%) | 184 (9.0%) | 65 (6.4%) | 237 (10.9%) | 65 (6.1%) |
| **ICU admissions, n (%)** | | | | | | | | |
| Overall | 2,531 (0.4%) | 3,315 (0.5%) | 2,692 (0.4%) | 3,494 (0.5%) | 2,882 (0.4%) | 3,597 (0.5%) | 2,845 (0.4%) | 3,535 (0.5%) |
| 20 | 97 (0.1%) | 137 (0.1%) | 101 (0.1%) | 142 (0.1%) | 96 (0.1%) | 128 (0.1%) | 105 (0.1%) | 135 (0.1%) |
| 30 | 121 (0.1%) | 100 (0.1%) | 118 (0.1%) | 133 (0.1%) | 123 (0.1%) | 135 (0.1%) | 129 (0.1%) | 136 (0.1%) |
| 40 | 172 (0.1%) | 190 (0.2%) | 192 (0.2%) | 211 (0.2%) | 174 (0.1%) | 199 (0.2%) | 174 (0.2%) | 170 (0.1%) |
| 50 | 303 (0.2%) | 480 (0.4%) | 330 (0.3%) | 512 (0.4%) | 334 (0.3%) | 524 (0.4%) | 365 (0.3%) | 499 (0.4%) |
| 60 | 451 (0.5%) | 721 (0.8%) | 487 (0.5%) | 727 (0.8%) | 531 (0.6%) | 797 (0.8%) | 513 (0.5%) | 800 (0.8%) |
| 70 | 515 (0.9%) | 786 (1.5%) | 609 (1.1%) | 819 (1.6%) | 666 (1.1%) | 893 (1.6%) | 666 (1.1%) | 907 (1.5%) |
| 80 | 617 (1.6%) | 745 (2.4%) | 598 (1.6%) | 758 (2.5%) | 681 (1.7%) | 746 (2.4%) | 647 (1.7%) | 694 (2.2%) |
| 90 | 251 (1.5%) | 153 (1.9%) | 246 (1.5%) | 191 (2.2%) | 272 (1.5%) | 172 (1.9%) | 242 (1.3%) | 193 (2.0%) |
| 100 | <=5 (0.2%) | <=5 (0.4%) | 11 (0.6%) | <=5 (0.1%) | <=5 (0.2%) | <=5 (0.3%) | <=5 (0.2%) | <=5 (0.1%) |
| **Receipt of IMV, n (%)** | | | | | | | | |
| Overall | 1,237 (0.2%) | 1,881 (0.3%) | 1,305 (0.2%) | 1,953 (0.3%) | 1,448 (0.2%) | 2,241 (0.3%) | 1,500 (0.2%) | 2,191 (0.3%) |
| 20 | 37 (<0.1%) | 62 (<0.1%) | 38 (<0.1%) | 63 (<0.1%) | 35 (<0.1%) | 73 (0.1%) | 39 (<0.1%) | 75 (0.1%) |
| 30 | 47 (<0.1%) | 56 (<0.1%) | 51 (<0.1%) | 72 (0.1%) | 65 (0.1%) | 77 (0.1%) | 60 (0.1%) | 72 (0.1%) |
| 40 | 86 (0.1%) | 98 (0.1%) | 82 (0.1%) | 110 (0.1%) | 77 (0.1%) | 110 (0.1%) | 79 (0.1%) | 96 (0.1%) |
| 50 | 167 (0.1%) | 282 (0.2%) | 170 (0.1%) | 277 (0.2%) | 162 (0.1%) | 348 (0.3%) | 196 (0.1%) | 317 (0.2%) |
| 60 | 243 (0.3%) | 436 (0.5%) | 256 (0.3%) | 457 (0.5%) | 300 (0.3%) | 509 (0.5%) | 309 (0.3%) | 550 (0.6%) |
| 70 | 266 (0.5%) | 475 (0.9%) | 334 (0.6%) | 506 (1.0%) | 360 (0.6%) | 586 (1.0%) | 376 (0.6%) | 621 (1.0%) |
| 80 | 296 (0.8%) | 415 (1.3%) | 292 (0.8%) | 393 (1.3%) | 340 (0.9%) | 461 (1.5%) | 337 (0.9%) | 392 (1.2%) |
| 90 | 94 (0.6%) | 56 (0.7%) | 79 (0.5%) | 75 (0.9%) | 108 (0.6%) | 77 (0.9%) | 104 (0.6%) | 67 (0.7%) |
| 100 | <=5 (0.1%) | <=5 (0.1%) | <=5 (0.2%) | 0 (<0.1%) | <=5 (<0.1%) | 0 (<0.1%) | 0 (<0.1%) | <=5 (0.1%) |
| **Death, n (%)** | | | | | | | | |
| Overall | 4,519 (0.7%) | 4,465 (0.7%) | 4,538 (0.7%) | 4,603 (0.7%) | 4,647 (0.7%) | 4,598 (0.7%) | 4,827 (0.7%) | 4,536 (0.7%) |
| 20 | 17 (<0.1%) | 52 (<0.1%) | 22 (<0.1%) | 66 (0.1%) | 13 (<0.1%) | 44 (<0.1%) | 17 (<0.1%) | 42 (<0.1%) |
| 30 | 30 (<0.1%) | 43 (<0.1%) | 34 (<0.1%) | 63 (0.1%) | 30 (<0.1%) | 71 (0.1%) | 37 (<0.1%) | 48 (<0.1%) |
| 40 | 59 (<0.1%) | 100 (0.1%) | 72 (0.1%) | 115 (0.1%) | 78 (0.1%) | 88 (0.1%) | 64 (0.1%) | 97 (0.1%) |
| 50 | 217 (0.2%) | 310 (0.2%) | 211 (0.2%) | 339 (0.3%) | 206 (0.2%) | 326 (0.2%) | 229 (0.2%) | 272 (0.2%) |
| 60 | 356 (0.4%) | 611 (0.7%) | 407 (0.4%) | 619 (0.7%) | 441 (0.5%) | 666 (0.7%) | 439 (0.4%) | 571 (0.6%) |
| 70 | 641 (1.2%) | 887 (1.7%) | 612 (1.1%) | 908 (1.7%) | 640 (1.1%) | 940 (1.7%) | 731 (1.2%) | 901 (1.5%) |
| 80 | 1,294 (3.3%) | 1,422 (4.6%) | 1,224 (3.2%) | 1,409 (4.6%) | 1,267 (3.2%) | 1,368 (4.4%) | 1,202 (3.1%) | 1,415 (4.5%) |
| 90 | 1,718 (10.6%) | 982 (11.9%) | 1,737 (10.3%) | 1,044 (11.8%) | 1,746 (9.9%) | 1,026 (11.4%) | 1,838 (10.1%) | 1,132 (11.7%) |
| 100 | 187 (10.9%) | 58 (7.1%) | 219 (12.9%) | 40 (4.8%) | 226 (11.1%) | 69 (6.8%) | 270 (12.4%) | 58 (5.5%) |

|  | **2015** | | **2016** | | **2017** | | **2018** | |
| --- | --- | --- | --- | --- | --- | --- | --- | --- |
|  | **Women (n=702,288)** | **Men (n=690,108)** | **Women (n=696,915)** | **Men (n=683,228)** | **Women (n=701,191)** | **Men (n=687,983)** | **Women (n=703,244)** | **Men (n=690,721)** |
| **ED Visits, n (%)** | | | | | | | | |
| Overall | 142,940 (20.4%) | 123,066 (17.8%) | 144,516 (20.7%) | 125,806 (18.4%) | 147,414 (21.0%) | 128,154 (18.6%) | 148,308 (21.1%) | 128,249 (18.6%) |
| 20 | 23,212 (22.1%) | 20,925 (19.0%) | 23,484 (23.1%) | 21,185 (20.0%) | 22,869 (23.8%) | 20,450 (20.4%) | 21,877 (23.9%) | 19,128 (19.8%) |
| 30 | 22,350 (18.6%) | 18,365 (15.2%) | 22,696 (18.9%) | 19,232 (16.0%) | 23,089 (19.3%) | 19,279 (16.0%) | 23,072 (19.5%) | 19,242 (16.1%) |
| 40 | 19,156 (16.6%) | 16,923 (14.7%) | 19,601 (17.1%) | 17,414 (15.3%) | 19,768 (17.4%) | 17,228 (15.4%) | 19,921 (17.7%) | 17,471 (15.7%) |
| 50 | 23,605 (17.8%) | 21,401 (15.6%) | 22,694 (17.9%) | 20,688 (15.8%) | 21,529 (17.8%) | 19,551 (15.7%) | 20,755 (17.5%) | 18,766 (15.5%) |
| 60 | 19,771 (19.0%) | 18,184 (17.9%) | 20,503 (19.3%) | 19,098 (18.2%) | 21,264 (19.5%) | 20,131 (18.5%) | 21,826 (19.4%) | 20,676 (18.2%) |
| 70 | 14,600 (22.7%) | 13,325 (21.8%) | 14,828 (22.8%) | 13,630 (22.3%) | 18,015 (22.9%) | 16,362 (22.0%) | 19,360 (22.8%) | 17,623 (22.1%) |
| 80 | 12,400 (31.6%) | 9,977 (30.9%) | 12,842 (31.8%) | 10,362 (31.1%) | 12,894 (31.5%) | 10,740 (31.0%) | 13,269 (31.6%) | 10,696 (30.5%) |
| 90 | 7,453 (38.9%) | 3,864 (38.0%) | 7,488 (38.0%) | 4,096 (36.7%) | 7,571 (38.0%) | 4,290 (37.0%) | 7,825 (38.5%) | 4,526 (36.8%) |
| 100 | 393 (16.0%) | 102 (7.7%) | 380 (15.1%) | 101 (7.7%) | 415 (16.6%) | 123 (8.7%) | 403 (15.3%) | 121 (8.0%) |
| **Hospital admissions, n (%)** | | | | | | | | |
| Overall | 43,051 (6.1%) | 26,657 (3.9%) | 42,484 (6.1%) | 26,762 (3.9%) | 43,139 (6.2%) | 27,978 (4.1%) | 43,723 (6.2%) | 28,473 (4.1%) |
| 20 | 3,242 (3.1%) | 1,290 (1.2%) | 3,190 (3.1%) | 1,159 (1.1%) | 2,872 (3.0%) | 1,162 (1.2%) | 2,663 (2.9%) | 1,090 (1.1%) |
| 30 | 11,919 (9.9%) | 1,234 (1.0%) | 11,992 (10.0%) | 1,298 (1.1%) | 11,912 (10.0%) | 1,295 (1.1%) | 11,889 (10.0%) | 1,343 (1.1%) |
| 40 | 4,242 (3.7%) | 1,738 (1.5%) | 4,104 (3.6%) | 1,751 (1.5%) | 4,109 (3.6%) | 1,646 (1.5%) | 4,171 (3.7%) | 1,720 (1.5%) |
| 50 | 3,834 (2.9%) | 3,695 (2.7%) | 3,652 (2.9%) | 3,484 (2.7%) | 3,270 (2.7%) | 3,226 (2.6%) | 3,132 (2.6%) | 3,178 (2.6%) |
| 60 | 4,759 (4.6%) | 5,474 (5.4%) | 4,840 (4.6%) | 5,659 (5.4%) | 4,805 (4.4%) | 5,792 (5.3%) | 5,028 (4.5%) | 5,920 (5.2%) |
| 70 | 5,253 (8.2%) | 5,790 (9.5%) | 5,070 (7.8%) | 5,734 (9.4%) | 6,302 (8.0%) | 6,959 (9.4%) | 6,732 (7.9%) | 7,289 (9.1%) |
| 80 | 5,532 (14.1%) | 5,066 (15.7%) | 5,440 (13.5%) | 5,187 (15.6%) | 5,535 (13.5%) | 5,323 (15.4%) | 5,688 (13.5%) | 5,192 (14.8%) |
| 90 | 4,038 (21.1%) | 2,299 (22.6%) | 3,971 (20.1%) | 2,423 (21.7%) | 4,087 (20.5%) | 2,487 (21.4%) | 4,186 (20.6%) | 2,675 (21.7%) |
| 100 | 232 (9.4%) | 71 (5.4%) | 225 (8.9%) | 67 (5.1%) | 247 (9.9%) | 88 (6.2%) | 234 (8.9%) | 66 (4.4%) |
| **ICU admissions, n (%)** | | | | | | | | |
| Overall | 2,863 (0.4%) | 3,636 (0.5%) | 2,738 (0.4%) | 3,579 (0.5%) | 2,851 (0.4%) | 3,646 (0.5%) | 3,003 (0.4%) | 3,853 (0.6%) |
| 20 | 112 (0.1%) | 161 (0.1%) | 114 (0.1%) | 145 (0.1%) | 115 (0.1%) | 128 (0.1%) | 109 (0.1%) | 140 (0.1%) |
| 30 | 136 (0.1%) | 132 (0.1%) | 146 (0.1%) | 154 (0.1%) | 151 (0.1%) | 164 (0.1%) | 141 (0.1%) | 179 (0.1%) |
| 40 | 174 (0.2%) | 222 (0.2%) | 174 (0.2%) | 224 (0.2%) | 158 (0.1%) | 193 (0.2%) | 184 (0.2%) | 202 (0.2%) |
| 50 | 360 (0.3%) | 495 (0.4%) | 347 (0.3%) | 470 (0.4%) | 269 (0.2%) | 433 (0.3%) | 303 (0.3%) | 427 (0.4%) |
| 60 | 524 (0.5%) | 812 (0.8%) | 543 (0.5%) | 844 (0.8%) | 518 (0.5%) | 837 (0.8%) | 580 (0.5%) | 845 (0.7%) |
| 70 | 644 (1.0%) | 888 (1.5%) | 587 (0.9%) | 845 (1.4%) | 721 (0.9%) | 948 (1.3%) | 757 (0.9%) | 1,106 (1.4%) |
| 80 | 631 (1.6%) | 728 (2.3%) | 571 (1.4%) | 690 (2.1%) | 620 (1.5%) | 714 (2.1%) | 621 (1.5%) | 727 (2.1%) |
| 90 | 276 (1.4%) | 193 (1.9%) | 250 (1.3%) | 202 (1.8%) | 290 (1.5%) | 225 (1.9%) | 302 (1.5%) | 224 (1.8%) |
| 100 | 6 (0.2%) | <=5 (0.4%) | 6 (0.2%) | <=5 (0.4%) | 9 (0.4%) | <=5 (0.3%) | 6 (0.2%) | <=5 (0.2%) |
| **Receipt of IMV, n (%)** | | | | | | | | |
| Overall | 1,520 (0.2%) | 2,274 (0.3%) | 1,507 (0.2%) | 2,381 (0.3%) | 1,540 (0.2%) | 2,449 (0.4%) | 1,690 (0.2%) | 2,542 (0.4%) |
| 20 | 46 (<0.1%) | 95 (0.1%) | 57 (0.1%) | 81 (0.1%) | 44 (<0.1%) | 84 (0.1%) | 49 (0.1%) | 75 (0.1%) |
| 30 | 61 (0.1%) | 76 (0.1%) | 71 (0.1%) | 100 (0.1%) | 80 (0.1%) | 87 (0.1%) | 72 (0.1%) | 112 (0.1%) |
| 40 | 90 (0.1%) | 135 (0.1%) | 81 (0.1%) | 139 (0.1%) | 79 (0.1%) | 137 (0.1%) | 87 (0.1%) | 132 (0.1%) |
| 50 | 194 (0.1%) | 314 (0.2%) | 178 (0.1%) | 322 (0.2%) | 154 (0.1%) | 268 (0.2%) | 154 (0.1%) | 300 (0.2%) |
| 60 | 321 (0.3%) | 569 (0.6%) | 323 (0.3%) | 615 (0.6%) | 307 (0.3%) | 601 (0.6%) | 326 (0.3%) | 579 (0.5%) |
| 70 | 364 (0.6%) | 586 (1.0%) | 355 (0.5%) | 590 (1.0%) | 414 (0.5%) | 689 (0.9%) | 478 (0.6%) | 791 (1.0%) |
| 80 | 339 (0.9%) | 414 (1.3%) | 324 (0.8%) | 426 (1.3%) | 347 (0.8%) | 467 (1.3%) | 380 (0.9%) | 448 (1.3%) |
| 90 | 101 (0.5%) | 84 (0.8%) | 115 (0.6%) | 106 (1.0%) | 107 (0.5%) | 115 (1.0%) | 141 (0.7%) | 103 (0.8%) |
| 100 | <=5 (0.2%) | <=5 (0.1%) | <=5 (0.1%) | <=5 (0.2%) | 8 (0.3%) | <=5 (0.1%) | <=5 (0.1%) | <=5 (0.1%) |
| **Death, n (%)** | | | | | | | | |
| Overall | 5,053 (0.7%) | 4,867 (0.7%) | 4,849 (0.7%) | 4,978 (0.7%) | 5,088 (0.7%) | 5,267 (0.8%) | 5,315 (0.8%) | 5,400 (0.8%) |
| 20 | 15 (<0.1%) | 47 (<0.1%) | 23 (<0.1%) | 59 (0.1%) | 26 (<0.1%) | 42 (<0.1%) | 14 (<0.1%) | 63 (0.1%) |
| 30 | 52 (<0.1%) | 47 (<0.1%) | 46 (<0.1%) | 84 (0.1%) | 38 (<0.1%) | 103 (0.1%) | 55 (<0.1%) | 94 (0.1%) |
| 40 | 72 (0.1%) | 100 (0.1%) | 68 (0.1%) | 128 (0.1%) | 70 (0.1%) | 115 (0.1%) | 64 (0.1%) | 135 (0.1%) |
| 50 | 236 (0.2%) | 332 (0.2%) | 191 (0.2%) | 303 (0.2%) | 152 (0.1%) | 277 (0.2%) | 178 (0.2%) | 269 (0.2%) |
| 60 | 450 (0.4%) | 709 (0.7%) | 469 (0.4%) | 719 (0.7%) | 455 (0.4%) | 686 (0.6%) | 469 (0.4%) | 715 (0.6%) |
| 70 | 701 (1.1%) | 943 (1.5%) | 693 (1.1%) | 947 (1.6%) | 852 (1.1%) | 1,174 (1.6%) | 918 (1.1%) | 1,174 (1.5%) |
| 80 | 1,261 (3.2%) | 1,412 (4.4%) | 1,237 (3.1%) | 1,374 (4.1%) | 1,222 (3.0%) | 1,418 (4.1%) | 1,307 (3.1%) | 1,448 (4.1%) |
| 90 | 1,981 (10.3%) | 1,203 (11.8%) | 1,857 (9.4%) | 1,294 (11.6%) | 1,972 (9.9%) | 1,366 (11.8%) | 2,012 (9.9%) | 1,435 (11.7%) |
| 100 | 285 (11.6%) | 74 (5.6%) | 265 (10.5%) | 70 (5.3%) | 301 (12.1%) | 86 (6.1%) | 298 (11.3%) | 67 (4.4%) |

|  | **2019** | |
| --- | --- | --- |
|  | **Women (n=704,429)** | **Men (n=692,475)** |
| **ED Visits, n (%)** | | |
| Overall | 149,291 (21.2%) | 128,825 (18.6%) |
| 20 | 21,791 (24.4%) | 19,314 (20.5%) |
| 30 | 23,790 (20.0%) | 19,761 (16.4%) |
| 40 | 20,018 (17.8%) | 17,410 (15.7%) |
| 50 | 21,177 (17.6%) | 18,706 (15.3%) |
| 60 | 22,218 (19.5%) | 20,950 (18.1%) |
| 70 | 18,611 (22.6%) | 16,736 (21.5%) |
| 80 | 13,581 (31.1%) | 11,250 (30.4%) |
| 90 | 7,672 (37.6%) | 4,570 (36.4%) |
| 100 | 433 (15.0%) | 128 (7.7%) |
| **Hospital admissions, n (%)** | | |
| Overall | 43,471 (6.2%) | 28,408 (4.1%) |
| 20 | 2,592 (2.9%) | 1,069 (1.1%) |
| 30 | 12,221 (10.3%) | 1,273 (1.1%) |
| 40 | 4,218 (3.8%) | 1,697 (1.5%) |
| 50 | 3,210 (2.7%) | 3,037 (2.5%) |
| 60 | 4,769 (4.2%) | 5,936 (5.1%) |
| 70 | 6,389 (7.8%) | 7,025 (9.0%) |
| 80 | 5,740 (13.1%) | 5,588 (15.1%) |
| 90 | 4,093 (20.1%) | 2,704 (21.5%) |
| 100 | 239 (8.3%) | 79 (4.8%) |
| **ICU admissions, n (%)** | | |
| Overall | 3,021 (0.4%) | 3,798 (0.5%) |
| 20 | 105 (0.1%) | 121 (0.1%) |
| 30 | 162 (0.1%) | 144 (0.1%) |
| 40 | 186 (0.2%) | 212 (0.2%) |
| 50 | 306 (0.3%) | 418 (0.3%) |
| 60 | 551 (0.5%) | 875 (0.8%) |
| 70 | 755 (0.9%) | 1,019 (1.3%) |
| 80 | 657 (1.5%) | 768 (2.1%) |
| 90 | 290 (1.4%) | 235 (1.9%) |
| 100 | 9 (0.3%) | 6 (0.4%) |
| **Receipt of IMV, n (%)** | | |
| Overall | 1,536 (0.2%) | 2,506 (0.4%) |
| 20 | 47 (0.1%) | 81 (0.1%) |
| 30 | 67 (0.1%) | 91 (0.1%) |
| 40 | 83 (0.1%) | 136 (0.1%) |
| 50 | 151 (0.1%) | 275 (0.2%) |
| 60 | 291 (0.3%) | 643 (0.6%) |
| 70 | 441 (0.5%) | 723 (0.9%) |
| 80 | 328 (0.8%) | 436 (1.2%) |
| 90 | 124 (0.6%) | 119 (0.9%) |
| 100 | <=5 (0.1%) | <=5 (0.1%) |
| **Death, n (%)** | | |
| Overall | 5,174 (0.7%) | 5,498 (0.8%) |
| 20 | 28 (<0.1%) | 70 (0.1%) |
| 30 | 42 (<0.1%) | 82 (0.1%) |
| 40 | 73 (0.1%) | 149 (0.1%) |
| 50 | 185 (0.2%) | 291 (0.2%) |
| 60 | 451 (0.4%) | 709 (0.6%) |
| 70 | 830 (1.0%) | 1,131 (1.5%) |
| 80 | 1,307 (3.0%) | 1,528 (4.1%) |
| 90 | 1,939 (9.5%) | 1,455 (11.6%) |
| 100 | 319 (11.0%) | 83 (5.0%) |
