# Supplementary material for: Acute healthcare resource utilization by age: A cohort study
Source: PLoS One. 2021 May 19;16(5):e0251877. doi: 10.1371/journal.pone.0251877 (PMC8133481; doi:10.1371/journal.pone.0251877)
Supplement: S3 Table — (DOCX) [file pone.0251877.s007.docx]

**S3 Table.** Proportion of the population experiencing at least one major healthcare encounters per year

|  | **1995**  **(n=1,113,313)** | **1996**  **(n=1,107,877)** | **1997**  **(n=1,131,845)** | **1998**  **(n=1,146,408)** | **1999**  **(n=1,155,970)** | **2000**  **(n=1,174,520)** | **2001**  **(n=1,217,276)** | **2002**  **(n=1,215,802)** |
| --- | --- | --- | --- | --- | --- | --- | --- | --- |
| **ED visits, n (%)** | NA | NA | NA | NA | NA | NA | NA | NA |
| **Hospital admissions, n (%)** | 74,419 (6.7) | 69,591 (6.3) | 67,305 (5.9) | 65,729 (5.7) | 65,313 (5.7) | 65,349 (5.6) | 68,707 (5.6) | 66,541 (5.5) |
| **ICU admissions, n (%)** | NA | NA | NA | NA | NA | NA | NA | NA |
| **Receipt of IMV, n (%)** | 2,210 (0.2) | 2,225 (0.2) | 2,292 (0.2) | 2,254 (0.2) | 2,292 (0.2) | 2,319 (0.2) | 2,413 (0.2) | 2,453 (0.2) |
| **Death, n (%)** | 7,896 (0.7) | 7,739 (0.7) | 7,731 (0.7) | 7,653 (0.7) | 7,813 (0.7) | 7,655 (0.7) | 8,209 (0.7) | 7,880 (0.6) |

|  | **2003**  **(n=1,250,737)** | **2004**  **(n=1,260,283)** | **2005**  **(n=1,276,493)** | **2006**  **(n=1,269,830)** | **2007**  **(n=1,289,727)** | **2008**  **(n=1,301,954)** | **2009  (n=1,309,856)** | **2010**  **(n=1,330,594)** |
| --- | --- | --- | --- | --- | --- | --- | --- | --- |
| **ED visits, n (%)** | 205,474 (16.4) | 213,607 (16.9) | 222,398 (17.4) | 222,761 (17.5) | 225,770 (17.5) | 227,296 (17.5) | 230,396 (17.6) | 237,257 (17.8) |
| **Hospital admissions, n (%)** | 66,354 (5.3) | 66,823 (5.3) | 68,085 (5.3) | 65,466 (5.2) | 65,451 (5.1) | 64,792 (5.0) | 64,961 (5.0) | 64,595 (4.9) |
| **ICU admissions, n (%)** | 6,019 (0.5) | 5,892 (0.5) | 5,913 (0.5) | 5,766 (0.5) | 5,747 (0.4) | 5,658 (0.4) | 5,749 (0.4) | 5,752 (0.4) |
| **Receipt of IMV, n (%)** | 2,464 (0.2) | 2,486 (0.2) | 2,651 (0.2) | 2,706 (0.2) | 2,798 (0.2) | 2,865 (0.2) | 3,007 (0.2) | 2,958 (0.2) |
| **Death, n (%)** | 8,474 (0.7) | 8,335 (0.7) | 8,395 (0.7) | 8,380 (0.7) | 8,523 (0.7) | 8,605 (0.7) | 8,537 (0.7) | 8,504 (0.6) |

|  | **2011 (n=1,366,196)** | **2012 (n=1,370,354)** | **2013 (n=1,388,431)** | **2014 (n=1,380,139)** | **2015 (n=1,392,396)** | **2016 (n=1,380,143)** | **2017 (n=1,389,174)** | **2018 (n=1,393,965)** |
| --- | --- | --- | --- | --- | --- | --- | --- | --- |
| **ED visits, n (%)** | 247,273 (18.1) | 252,739 (18.4) | 256,870 (18.5) | 261,980 (19.0) | 266,006 (19.1) | 270,322 (19.6) | 275,568 (19.8) | 276,557 (19.8) |
| **Hospital admissions, n (%)** | 67,379 (4.9) | 67,762 (4.9) | 68,852 (5.0) | 69,309 (5.0) | 69,708 (5.0) | 69,246 (5.0) | 71,117 (5.1) | 72,196 (5.2) |
| **ICU admissions, n (%)** | 5,846 (0.4) | 6,186 (0.5) | 6,479 (0.5) | 6,380 (0.5) | 6,499 (0.5) | 6,317 (0.5) | 6,497 (0.5) | 6,856 (0.5) |
| **Receipt of IMV, n (%)** | 3,118 (0.2) | 3,258 (0.2) | 3,689 (0.3) | 3,691 (0.3) | 3,794 (0.3) | 3,888 (0.3) | 3,989 (0.3) | 4,232 (0.3) |
| **Death, n (%)** | 8,984 (0.7) | 9,141 (0.7) | 9,245 (0.7) | 9,363 (0.7) | 9,920 (0.7) | 9,827 (0.7) | 10,355 (0.7) | 10,715 (0.8) |

|  | **2019 (n=1,396,904)** |
| --- | --- |
| **ED visits, n (%)** | 278,116 (19.9) |
| **Hospital admissions, n (%)** | 71,879 (5.1) |
| **ICU admissions, n (%)** | 6,819 (0.5) |
| **Receipt of IMV, n (%)** | 4,042 (0.3) |
| **Death, n (%)** | 10,672 (0.8) |
