# Supplementary material for: Acute healthcare resource utilization by age: A cohort study
Source: PLoS One. 2021 May 19;16(5):e0251877. doi: 10.1371/journal.pone.0251877 (PMC8133481; doi:10.1371/journal.pone.0251877)
Supplement: S4 Table — (DOCX) [file pone.0251877.s008.docx]

**S4 Table.** Annual rates of acute healthcare and critical care resource utilization stratified by age

|  | **1995** | **1996** | **1997** | **1998** | **1999** | **2000** | **2001** | **2002** |
| --- | --- | --- | --- | --- | --- | --- | --- | --- |
| **ED visits, rate per 1,000 people (95% CI)** | | | | | | | | |
| Overall | NA | NA | NA | NA | NA | NA | NA | NA |
| 20 | NA | NA | NA | NA | NA | NA | NA | NA |
| 30 | NA | NA | NA | NA | NA | NA | NA | NA |
| 40 | NA | NA | NA | NA | NA | NA | NA | NA |
| 50 | NA | NA | NA | NA | NA | NA | NA | NA |
| 60 | NA | NA | NA | NA | NA | NA | NA | NA |
| 70 | NA | NA | NA | NA | NA | NA | NA | NA |
| 80 | NA | NA | NA | NA | NA | NA | NA | NA |
| 90 | NA | NA | NA | NA | NA | NA | NA | NA |
| 100 | NA | NA | NA | NA | NA | NA | NA | NA |
| **Hospital admissions, rate per 1,000 people (95% CI)** | | | | | | | | |
| Overall | 66.8 (66.4 – 67.3) | 62.8 (62.4 – 63.3) | 59.5 (59.0 – 59.9) | 57.3 (56.9 – 57.8) | 56.5 (56.1 – 56.9) | 55.6 (55.2 – 56.1) | 56.4 (56.0 – 56.9) | 54.7 (54.3 – 55.1) |
| 20 | 34.1 (33.4 – 34.9) | 32.0 (31.3 – 32.7) | 30.1 (29.4 – 30.8) | 29.8 (29.1 – 30.5) | 28.3 (27.6 – 29.0) | 27.7 (27.1 – 28.4) | 26.8 (26.2 – 27.5) | 25.6 (25.0 – 26.3) |
| 30 | 66.7 (65.8 – 67.7) | 61.9 (61.0 – 62.9) | 59.0 (58.1 – 59.9) | 56.0 (55.1 – 56.9) | 55.3 (54.4 – 56.2) | 54.1 (53.2 – 55.0) | 56.0 (55.1 – 56.9) | 54.2 (53.3 – 55.1) |
| 40 | 39.6 (38.7 – 40.4) | 36.2 (35.4 – 36.9) | 33.9 (33.2 – 34.6) | 33.4 (32.7 – 34.2) | 32.0 (31.3 – 32.7) | 31.4 (30.7 – 32.1) | 31.2 (30.5 – 31.8) | 30.7 (30.0 – 31.4) |
| 50 | 51.2 (50.0 – 52.3) | 46.2 (45.1 – 47.3) | 44.6 (43.6 – 45.6) | 42.9 (42.0 – 43.8) | 40.9 (40.0 – 41.8) | 41.5 (40.6 – 42.4) | 40.7 (39.8 – 41.6) | 38.7 (37.8 – 39.6) |
| 60 | 79.8 (78.1 – 81.4) | 77.3 (75.7 – 79.0) | 72.3 (70.7 – 73.8) | 69.9 (68.4 – 71.4) | 68.8 (67.4 – 70.3) | 66.3 (64.9 – 67.8) | 67.0 (65.6 – 68.4) | 64.7 (63.3 – 66.1) |
| 70 | 140.2  (137.9 – 142.6) | 134.8  (132.5 – 137.1) | 126.9  (124.7 – 129.2) | 124.7  (122.5 – 126.9) | 123.1  (120.9 – 125.3) | 120  (117.8 – 122.1) | 116.4  (114.3 – 118.4) | 112.8  (110.8 – 114.9) |
| 80 | 219.4  (215.4 – 223.3) | 208.8  (204.9 – 212.7) | 206.7  (202.8 – 210.6) | 200.2  (196.3 – 204.1) | 199.6  (195.8 – 203.4) | 195.4  (191.7 – 199.0) | 190.8  (187.5 – 194.1) | 186.4  (183.2 – 189.6) |
| 90 | 273.9  (265.1 – 282.8) | 268.3  (259.7 – 277) | 261.3  (253.0 – 269.7) | 252.3  (244.2 – 260.5) | 250.5  (242.6 – 258.3) | 253.9  (246.1 – 261.8) | 250.5  (243 – 257.9) | 247.2  (239.8 – 254.6) |
| 100 | 163.1  (129.8 – 196.5) | 170.6  (137.9 – 203.3) | 113.9  (87.4 – 140.4) | 148.8  (120.3 – 177.4) | 146.5  (117.9 – 175.1) | 157.4  (130.0 – 184.7) | 131.4  (107.7 – 155.2) | 115.6  (94.3 – 136.9) |
| **ICU admissions, rate per 1,000 people (95% CI)** | | | | | | | | |
| Overall | NA | NA | NA | NA | NA | NA | NA | NA |
| 20 | NA | NA | NA | NA | NA | NA | NA | NA |
| 30 | NA | NA | NA | NA | NA | NA | NA | NA |
| 40 | NA | NA | NA | NA | NA | NA | NA | NA |
| 50 | NA | NA | NA | NA | NA | NA | NA | NA |
| 60 | NA | NA | NA | NA | NA | NA | NA | NA |
| 70 | NA | NA | NA | NA | NA | NA | NA | NA |
| 80 | NA | NA | NA | NA | NA | NA | NA | NA |
| 90 | NA | NA | NA | NA | NA | NA | NA | NA |
| 100 | NA | NA | NA | NA | NA | NA | NA | NA |
| **Receipt of invasive mechanical ventilation, rate per 1,000 people (95% CI)** | | | | | | | | |
| Overall | 2.0 (1.9 – 2.1) | 2.0 (1.9 – 2.1) | 2.0 (1.9 – 2.1) | 2.0 (1.9 – 2.1) | 2.0 (1.9 – 2.1) | 2.0 (1.9 – 2.1) | 2.0 (1.9 – 2.1) | 2.0 (1.9 – 2.1) |
| 20 | 0.3 (0.3 – 0.4) | 0.3 (0.2 – 0.4) | 0.3 (0.3 – 0.4) | 0.4 (0.3 – 0.4) | 0.3 (0.2 – 0.4) | 0.4 (0.3 – 0.4) | 0.3 (0.2 – 0.4) | 0.4 (0.3 – 0.5) |
| 30 | 0.4 (0.3 – 0.5) | 0.4 (0.4 – 0.5) | 0.4 (0.3 – 0.5) | 0.3 (0.3 – 0.4) | 0.4 (0.3 – 0.5) | 0.4 (0.3 – 0.5) | 0.4 (0.3 – 0.4) | 0.4 (0.3 – 0.5) |
| 40 | 0.7 (0.6 – 0.8) | 0.7 (0.5 – 0.8) | 0.7 (0.6 – 0.8) | 0.6 (0.5 – 0.7) | 0.7 (0.6 – 0.8) | 0.6 (0.5 – 0.7) | 0.6 (0.5 – 0.7) | 0.7 (0.6 – 0.8) |
| 50 | 1.6 (1.4 – 1.8) | 1.5 (1.3 – 1.7) | 1.6 (1.4 – 1.8) | 1.6 (1.4 – 1.8) | 1.6 (1.4 – 1.8) | 1.7 (1.5 – 1.9) | 1.5 (1.3 – 1.6) | 1.7 (1.5 – 1.9) |
| 60 | 4.3 (3.9 – 4.7) | 4.3 (3.9 – 4.7) | 4.4 (4.0 – 4.8) | 3.9 (3.5 – 4.3) | 3.8 (3.5 – 4.2) | 4.0 (3.6 – 4.3) | 4.2 (3.8 – 4.5) | 3.9 (3.5 – 4.2) |
| 70 | 8.8 (8.1 – 9.4) | 8.7 (8.1 – 9.4) | 9.2 (8.5 – 9.8) | 8.9 (8.3 – 9.6) | 8.8 (8.1 – 9.4) | 7.7 (7.2 – 8.3) | 8.1 (7.5 – 8.7) | 7.9 (7.3 – 8.5) |
| 80 | 9.9 (9.0 – 10.9) | 10.1 (9.2 – 11.1) | 9.1 (8.2 – 10) | 10.0 (9.1 – 11) | 10.2 (9.2 – 11.2) | 10.4 (9.5 – 11.4) | 9.4 (8.6 – 10.2) | 9.2 (8.4 – 10) |
| 90 | 4.9 (3.5 – 6.3) | 6.2 (4.7 – 7.8) | 5.2 (3.8 – 6.6) | 5.3 (3.9 – 6.6) | 4.9 (3.7 – 6.2) | 6.7 (5.3 – 8.2) | 4.8 (3.6 – 6) | 5.3 (4.1 – 6.5) |
| 100 | 2.1 (0 – 6.2) | 2 (0 – 5.8) | 0 (0 – 0) | 0 (0 – 0) | 3.4 (0 – 8.1) | 0 (0 – 0) | 1.3 (0 – 3.8) | 0 (0 – 0) |
| **Death, rate per 1,000 people (95% CI)** | | | | | | | | |
| Overall | 7.1 (6.9 – 7.3) | 7.0 (6.8 – 7.1) | 6.8 (6.7 – 7.0) | 6.7 (6.5 – 6.8) | 6.8 (6.6 – 6.9) | 6.5 (6.4 – 6.7) | 6.7 (6.6 – 6.9) | 6.5 (6.3 – 6.6) |
| 20 | 0.3 (0.2 – 0.4) | 0.3 (0.3 – 0.4) | 0.3 (0.2 – 0.4) | 0.3 (0.3 – 0.4) | 0.3 (0.2 – 0.4) | 0.3 (0.3 – 0.4) | 0.3 (0.2 – 0.4) | 0.4 (0.3 – 0.4) |
| 30 | 0.5 (0.4 – 0.6) | 0.5 (0.4 – 0.5) | 0.4 (0.4 – 0.5) | 0.4 (0.4 – 0.5) | 0.4 (0.3 – 0.5) | 0.4 (0.3 – 0.4) | 0.4 (0.3 – 0.5) | 0.4 (0.3 – 0.4) |
| 40 | 1.3 (1.1 – 1.4) | 1.1 (0.9 – 1.2) | 1.0 (0.8 – 1.1) | 1.0 (0.8 – 1.1) | 1.1 (0.9 – 1.2) | 0.8 (0.7 – 0.9) | 0.8 (0.7 – 0.9) | 0.9 (0.8 – 1.0) |
| 50 | 2.9 (2.6 – 3.1) | 2.8 (2.6 – 3.1) | 2.4 (2.1 – 2.6) | 2.6 (2.4 – 2.9) | 2.7 (2.5 – 3.0) | 2.4 (2.2 – 2.6) | 2.6 (2.4 – 2.8) | 2.6 (2.3 – 2.8) |
| 60 | 8.1 (7.6 – 8.7) | 8.1 (7.5 – 8.6) | 7.9 (7.4 – 8.5) | 7.4 (6.9 – 7.9) | 7.5 (7.0 – 8.0) | 6.6 (6.2 – 7.1) | 6.8 (6.3 – 7.3) | 6.7 (6.2 – 7.1) |
| 70 | 23.0 (22.0 – 24.0) | 22.9 (21.9 – 24.0) | 21.2 (20.2 – 22.1) | 21.6 (20.6 – 22.6) | 20.7 (19.7 – 21.6) | 19.5 (18.6 – 20.5) | 18.7 (17.9 – 19.6) | 18.5 (17.6 – 19.4) |
| 80 | 61.7 (59.4 – 64.0) | 58.5 (56.2 – 60.8) | 60.0 (57.7 – 62.3) | 57.3 (55.0 – 59.6) | 57.2 (54.9 – 59.4) | 55 (52.9 – 57.1) | 52.1 (50.3 – 54.0) | 47.5 (45.7 – 49.2) |
| 90 | 157.1  (149.9 – 164.3) | 154.0  (147.0 – 161.1) | 154.7  (147.8 – 161.6) | 148.7  (142.0 – 155.3) | 143.8  (137.5 – 150.2) | 147.4  (141.0 – 153.8) | 137.3  (131.4 – 143.2) | 131.3  (125.5 – 137.1) |
| 100 | 237.3  (198.9 – 275.7) | 251.0  (213.3 – 288.6) | 256.8  (220.3 – 293.2) | 264.2  (228.8 – 299.6) | 260.6  (225.1 – 296.2) | 223.5  (192.2 – 254.9) | 216.5  (187.5 – 245.5) | 176.9  (151.4 – 202.3) |

|  | **2003** | **2004** | **2005** | **2006** | **2007** | **2008** | **2009** | **2010** |
| --- | --- | --- | --- | --- | --- | --- | --- | --- |
| **ED visits, rate per 1,000 people (95% CI)** | | | | | | | | |
| Overall | 164.3  (163.6 – 164.9) | 169.5  (168.8 – 170.1) | 174.2  (173.6 – 174.9) | 175.4  (174.8 – 176.1) | 175.1  (174.4 – 175.7) | 174.6  (173.9 – 175.2) | 175.9  (175.2 – 176.5) | 178.3  (177.7 – 179.0) |
| 20 | 156.4  (154.9 – 157.8) | 159.9  (158.4 – 161.3) | 166.6  (165.1 – 168.1) | 168.6  (167.1 – 170.1) | 170.0  (168.5 – 171.5) | 169.4  (167.9 – 170.9) | 173.7  (172.2 – 175.2) | 175.7  (174.2 – 177.2) |
| 30 | 139.5  (138.1 – 140.8) | 143.6  (142.1 – 145) | 149.0  (147.6 – 150.5) | 153.2  (151.7 – 154.7) | 151.0  (149.5 – 152.5) | 151.7  (150.2 – 153.2) | 154.5  (153.0 – 156.0) | 156.9  (155.4 – 158.4) |
| 40 | 143.8  (142.5 – 145.1) | 147.1  (145.8 – 148.4) | 150.9  (149.6 – 152.2) | 149.9  (148.6 – 151.3) | 146.5  (145.1 – 147.9) | 143.9  (142.5 – 145.3) | 143.6  (142.2 – 144.9) | 146.6  (145.2 – 148.0) |
| 50 | 150.3  (148.7 – 151.8) | 156.8  (155.3 – 158.4) | 159.7  (158.1 – 161.2) | 160.2  (158.7 – 161.7) | 160.6  (159.1 – 162.1) | 160.3  (158.8 – 161.8) | 162.2  (160.7 – 163.7) | 163.4  (162.0 – 164.9) |
| 60 | 168.5  (166.5 – 170.6) | 175.4  (173.4 – 177.5) | 178.9  (176.8 – 180.9) | 177.1  (175.1 – 179.1) | 177.6  (175.8 – 179.5) | 179.0  (177.2 – 180.7) | 174.8  (173.0 – 176.6) | 178.2  (176.4 – 180.0) |
| 70 | 213.3  (210.7 – 216) | 221.9  (219.2 – 224.6) | 222.9  (220.2 – 225.6) | 222.1  (219.4 – 224.8) | 222.2  (219.6 – 224.9) | 218.4  (215.8 – 221) | 219.8  (217.3 – 222.4) | 220.9  (218.3 – 223.4) |
| 80 | 308.8  (305 – 312.5) | 314.7  (310.9 – 318.4) | 319.1  (315.4 – 322.8) | 316.4  (312.8 – 320.1) | 314.5  (310.9 – 318.2) | 311.7  (308.1 – 315.3) | 311.1  (307.5 – 314.6) | 312.5  (309.0 – 316.1) |
| 90 | 369.8  (362.0 – 377.7) | 377.7  (370.0 – 385.4) | 380.5  (373.1 – 387.9) | 384.9  (377.5 – 392.3) | 378.4  (371.1 – 385.7) | 377.1  (369.8 – 384.3) | 375.7  (368.7 – 382.8) | 379.2  (372.5 – 386.0) |
| 100 | 179.1  (154.7 – 203.4) | 170.8  (148.8 – 192.8) | 180.7  (158.7 – 202.8) | 181.9  (161.3 – 202.5) | 157.3  (139.3 – 175.3) | 159.6  (141.8 – 177.4) | 144.4  (128.8 – 160.0) | 141.6  (126.5 – 156.7) |
| **Hospital admissions, rate per 1,000 people (95% CI)** | | | | | | | | |
| Overall | 53.1 (52.7 – 53.4) | 53.0 (52.6 – 53.4) | 53.3 (53.0 – 53.7) | 51.6 (51.2 – 51.9) | 50.8 (50.4 – 51.1) | 49.8 (49.4 – 50.1) | 49.6 (49.2 – 50.0) | 48.6 (48.2 – 48.9) |
| 20 | 24.6 (24.0 – 25.2) | 24.5 (23.8 – 25.1) | 24.7 (24.1 – 25.3) | 23.7 (23.1 – 24.3) | 22.8 (22.2 – 23.4) | 22.3 (21.7 – 22.9) | 21.7 (21.2 – 22.3) | 21.0 (20.5 – 21.6) |
| 30 | 54.2 (53.3 – 55.1) | 55.8 (54.8 – 56.7) | 57.5 (56.5 – 58.4) | 56.2 (55.2 – 57.1) | 56.2 (55.2 – 57.1) | 55.5 (54.5 – 56.4) | 55.5 (54.6 – 56.5) | 55.2 (54.3 – 56.2) |
| 40 | 29.3 (28.7 – 30.0) | 29.0 (28.4 – 29.6) | 29.1 (28.5 – 29.8) | 27.3 (26.7 – 27.9) | 25.9 (25.3 – 26.5) | 25.3 (24.7 – 25.9) | 25.5 (24.8 – 26.1) | 25.1 (24.4 – 25.7) |
| 50 | 37.1 (36.2 – 37.9) | 37.1 (36.3 – 38.0) | 35.4 (34.6 – 36.2) | 33.3 (32.5 – 34.0) | 32.0 (31.3 – 32.7) | 31.2 (30.5 – 31.9) | 31.7 (31.0 – 32.4) | 30.4 (29.8 – 31.1) |
| 60 | 62.9 (61.6 – 64.3) | 60.7 (59.4 – 62.0) | 61.3 (60.1 – 62.6) | 57.6 (56.4 – 58.8) | 56.4 (55.3 – 57.5) | 54.8 (53.8 – 55.9) | 53.9 (52.8 – 55.0) | 52.5 (51.4 – 53.5) |
| 70 | 109.4  (107.4 – 111.5) | 108.8  (106.8 – 110.9) | 107.7  (105.7 – 109.7) | 103.2  (101.2 – 105.1) | 100.9  (99.0 – 102.8) | 96.3 (94.4 – 98.2) | 95.7 (93.9 – 97.5) | 92.0 (90.3 – 93.8) |
| 80 | 178.4  (175.3 – 181.6) | 179.2  (176.1 – 182.4) | 176.0  (173.0 – 179.1) | 170.9  (168.0 – 173.9) | 167.7  (164.7 – 170.6) | 163.8  (160.9 – 166.6) | 159.3  (156.5 – 162.2) | 155.2  (152.5 – 158) |
| 90 | 236.1  (229.2 – 243.1) | 232.5  (225.8 – 239.2) | 236.2  (229.8 – 242.7) | 231.0  (224.6 – 237.4) | 220.6  (214.4 – 226.8) | 219.0  (212.8 – 225.2) | 217.5  (211.5 – 223.5) | 216.2  (210.5 – 222) |
| 100 | 97.4  (78.6 – 116.2) | 103.2  (85.4 – 121.0) | 112.5  (94.4 – 130.6) | 109.9  (93.2 – 126.6) | 94.9  (80.4 – 109.4) | 89.3  (75.5 – 103.2) | 88.3  (75.7 – 100.8) | 87.7  (75.4 – 100.0) |
| **ICU admissions, rate per 1,000 people (95% CI)** | | | | | | | | |
| Overall | 4.8 (4.7 – 4.9) | 4.7 (4.6 – 4.8) | 4.6 (4.5 – 4.8) | 4.5 (4.4 – 4.7) | 4.5 (4.3 – 4.6) | 4.4 (4.2 – 4.5) | 4.4 (4.3 – 4.5) | 4.3 (4.2 – 4.4) |
| 20 | 0.9 (0.7 – 1.0) | 0.9 (0.8 – 1.0) | 0.9 (0.7 – 1.0) | 1.0 (0.8 – 1.1) | 0.9 (0.8 – 1) | 0.8 (0.7 – 0.9) | 0.8 (0.7 – 0.9) | 0.9 (0.8 – 1.1) |
| 30 | 0.9 (0.8 – 1.0) | 0.9 (0.8 – 1.0) | 1.0 (0.8 – 1.1) | 1.0 (0.8 – 1.1) | 1.0 (0.9 – 1.2) | 0.7 (0.6 – 0.9) | 1.0 (0.9 – 1.1) | 1.1 (1.0 – 1.3) |
| 40 | 1.7 (1.5 – 1.8) | 1.6 (1.5 – 1.7) | 1.7 (1.6 – 1.9) | 1.6 (1.5 – 1.8) | 1.4 (1.3 – 1.6) | 1.4 (1.3 – 1.6) | 1.6 (1.5 – 1.8) | 1.4 (1.3 – 1.6) |
| 50 | 4.1 (3.8 – 4.4) | 3.7 (3.4 – 4.0) | 3.5 (3.3 – 3.8) | 3.5 (3.2 – 3.7) | 3.3 (3.0 – 3.5) | 3.3 (3.0 – 3.5) | 3.4 (3.2 – 3.6) | 3.3 (3.1 – 3.6) |
| 60 | 8.6 (8.1 – 9.1) | 7.9 (7.4 – 8.3) | 7.8 (7.3 – 8.3) | 7.4 (6.9 – 7.9) | 7.1 (6.7 – 7.6) | 6.7 (6.3 – 7.1) | 6.7 (6.3 – 7.1) | 6.4 (6.1 – 6.8) |
| 70 | 16.6 (15.8 – 17.4) | 16.6 (15.8 – 17.5) | 15.5 (14.7 – 16.3) | 14.3 (13.5 – 15.0) | 14.0 (13.2 – 14.7) | 13.8 (13.1 – 14.5) | 13.1 (12.4 – 13.8) | 13.1 (12.4 – 13.8) |
| 80 | 24.7 (23.4 – 26.0) | 23.9 (22.7 – 25.2) | 23.7 (22.5 – 24.9) | 23.6 (22.4 – 24.8) | 23.0 (21.8 – 24.2) | 22.0 (20.8 – 23.1) | 20.7 (19.6 – 21.8) | 19.7 (18.7 – 20.8) |
| 90 | 19.8 (17.5 – 22.0) | 19.4 (17.2 – 21.6) | 19.5 (17.4 – 21.6) | 18.5 (16.4 – 20.5) | 14.6 (12.8 – 16.4) | 14.6 (12.8 – 16.4) | 15.5 (13.7 – 17.3) | 17.0 (15.2 – 18.8) |
| 100 | 2.1 (0 – 5.0) | 7.1 (2.2 – 12.0) | 4.3 (0.5 – 8.0) | 3.0 (0.1 – 5.9) | 3.8 (0.8 – 6.9) | 4.3 (1.1 – 7.5) | 3.6 (0.9 – 6.2) | 4.9 (1.9 – 7.9) |
| **Receipt of invasive mechanical ventilation, rate per 1,000 people (95% CI)** | | | | | | | | |
| Overall | 2.0 (1.9 – 2.1) | 2.0 (1.9 – 2.1) | 2.1 (2.0 – 2.2) | 2.1 (2.1 – 2.2) | 2.2 (2.1 – 2.3) | 2.2 (2.1 – 2.3) | 2.3 (2.2 – 2.4) | 2.2 (2.1 – 2.3) |
| 20 | 0.3 (0.2 – 0.4) | 0.3 (0.3 – 0.4) | 0.4 (0.3 – 0.4) | 0.4 (0.4 – 0.5) | 0.4 (0.3 – 0.4) | 0.4 (0.3 – 0.4) | 0.4 (0.3 – 0.5) | 0.4 (0.4 – 0.5) |
| 30 | 0.3 (0.2 – 0.4) | 0.3 (0.3 – 0.4) | 0.4 (0.3 – 0.5) | 0.4 (0.3 – 0.5) | 0.5 (0.4 – 0.5) | 0.3 (0.2 – 0.4) | 0.5 (0.4 – 0.6) | 0.4 (0.4 – 0.5) |
| 40 | 0.7 (0.6 – 0.7) | 0.6 (0.5 – 0.7) | 0.7 (0.6 – 0.8) | 0.8 (0.7 – 0.9) | 0.6 (0.5 – 0.7) | 0.7 (0.6 – 0.8) | 0.8 (0.7 – 0.9) | 0.7 (0.6 – 0.8) |
| 50 | 1.7 (1.5 – 1.9) | 1.6 (1.4 – 1.7) | 1.7 (1.5 – 1.8) | 1.8 (1.7 – 2.0) | 1.6 (1.4 – 1.8) | 1.6 (1.5 – 1.8) | 1.8 (1.7 – 2.0) | 1.7 (1.5 – 1.9) |
| 60 | 3.9 (3.6 – 4.3) | 4.2 (3.8 – 4.5) | 4.0 (3.7 – 4.3) | 3.6 (3.3 – 3.9) | 3.9 (3.6 – 4.2) | 3.8 (3.5 – 4.1) | 4.1 (3.8 – 4.4) | 3.9 (3.6 – 4.2) |
| 70 | 7.4 (6.8 – 8.0) | 7.6 (7.0 – 8.2) | 7.6 (7.1 – 8.2) | 7.6 (7.0 – 8.2) | 7.4 (6.9 – 7.9) | 7.2 (6.7 – 7.7) | 7.4 (6.9 – 8.0) | 7.4 (6.9 – 8.0) |
| 80 | 9.3 (8.6 – 10.1) | 8.8 (8.0 – 9.5) | 9.8 (9.0 – 10.5) | 9.7 (9.0 – 10.5) | 10.4 (9.6 – 11.2) | 11.1 (10.3 – 11.9) | 9.8 (9.0 – 10.5) | 9.6 (8.8 – 10.3) |
| 90 | 5.4 (4.2 – 6.6) | 4.8 (3.7 – 5.9) | 4.5 (3.5 – 5.6) | 5.2 (4.1 – 6.3) | 4.7 (3.6 – 5.7) | 4.4 (3.4 – 5.4) | 5.3 (4.2 – 6.4) | 5.2 (4.2 – 6.2) |
| 100 | 0 (0 – 0) | 2.7 (0 – 5.6) | 0.9 (0 – 2.5) | 0.7 (0 – 2.2) | 1.3 (0 – 3.0) | 1.2 (0 – 2.9) | 0 (0 – 0) | 2.5 (0.3 – 4.6) |
| **Death, rate per 1,000 people (95% CI)** | | | | | | | | |
| Overall | 6.8 (6.6 – 6.9) | 6.6 (6.5 – 6.8) | 6.6 (6.4 – 6.7) | 6.6 (6.5 – 6.7) | 6.6 (6.5 – 6.8) | 6.6 (6.5 – 6.8) | 6.5 (6.4 – 6.7) | 6.4 (6.3 – 6.5) |
| 20 | 0.4 (0.3 – 0.5) | 0.3 (0.2 – 0.4) | 0.3 (0.3 – 0.4) | 0.3 (0.3 – 0.4) | 0.3 (0.2 – 0.4) | 0.3 (0.2 – 0.4) | 0.3 (0.2 – 0.3) | 0.3 (0.2 – 0.3) |
| 30 | 0.4 (0.3 – 0.5) | 0.4 (0.3 – 0.5) | 0.4 (0.4 – 0.5) | 0.4 (0.3 – 0.4) | 0.4 (0.3 – 0.5) | 0.4 (0.3 – 0.5) | 0.3 (0.3 – 0.4) | 0.4 (0.3 – 0.5) |
| 40 | 0.9 (0.8 – 1.0) | 0.8 (0.7 – 0.9) | 0.8 (0.7 – 0.9) | 0.9 (0.8 – 1.0) | 0.9 (0.8 – 1.0) | 0.8 (0.7 – 0.9) | 0.9 (0.8 – 1.0) | 0.8 (0.7 – 0.9) |
| 50 | 2.5 (2.3 – 2.7) | 2.4 (2.2 – 2.6) | 2.3 (2.1 – 2.5) | 2.5 (2.3 – 2.7) | 2.4 (2.2 – 2.6) | 2.6 (2.4 – 2.8) | 2.4 (2.2 – 2.6) | 2.3 (2.1 – 2.5) |
| 60 | 6.9 (6.5 – 7.4) | 6.7 (6.2 – 7.1) | 5.9 (5.5 – 6.3) | 6.3 (5.9 – 6.7) | 6.0 (5.7 – 6.4) | 5.9 (5.5 – 6.2) | 5.6 (5.2 – 5.9) | 5.6 (5.2 – 5.9) |
| 70 | 18.9 (18.0 – 19.7) | 17.4 (16.5 – 18.2) | 16.7 (15.9 – 17.6) | 16.1 (15.3 – 16.9) | 16.0 (15.2 – 16.8) | 14.7 (13.9 – 15.4) | 14.8 (14.1 – 15.6) | 14.1 (13.4 – 14.9) |
| 80 | 49.6 (47.8 – 51.4) | 48.0 (46.3 – 49.7) | 46.9 (45.2 – 48.6) | 44.7 (43.1 – 46.3) | 43.7 (42.1 – 45.3) | 43.8 (42.2 – 45.4) | 43.2 (41.6 – 44.8) | 40.2 (38.7 – 41.7) |
| 90 | 132.7  (127.2 – 138.2) | 130.8  (125.5 – 136.2) | 127.1  (122.1 – 132.2) | 124.9  (119.9 – 129.9) | 122.5  (117.6 – 127.4) | 125.7  (120.7 – 130.6) | 117.5  (112.8 – 122.2) | 114.3  (109.8 – 118.7) |
| 100 | 160.2  (136.9 – 183.5) | 162.8  (141.2 – 184.4) | 162.0  (140.9 – 183.1) | 150.0  (130.9 – 169.0) | 156.1  (138.1 – 174.0) | 136.8  (120.1 – 153.5) | 121.4  (107.0 – 135.9) | 136.2  (121.3 – 151.1) |

|  | **2011** | **2012** | **2013** | **2014** | **2015** | **2016** | **2017** | **2018** |
| --- | --- | --- | --- | --- | --- | --- | --- | --- |
| **ED visits, rate per 1,000 people (95% CI)** | | | | | | | | |
| Overall | 181.0  (180.3 – 181.6) | 184.4  (183.8 – 185.1) | 185.0  (184.4 – 185.7) | 189.8  (189.2 – 190.5) | 191.0  (190.4 – 191.7) | 195.9  (195.2 – 196.5) | 198.4  (197.7 – 199.0) | 198.4  (197.7 – 199.1) |
| 20 | 180.8  (179.3 – 182.3) | 175.9  (174.4 – 177.4) | 182.9  (181.4 – 184.5) | 197.9  (196.2 – 199.6) | 204.7  (203.0 – 206.4) | 215.2  (213.4 – 217.0) | 220.4  (218.6 – 222.3) | 218.0  (216.1 – 219.8) |
| 30 | 159.4  (158.0 – 160.9) | 163.7  (162.2 – 165.2) | 164.4  (163.0 – 165.9) | 167.1  (165.6 – 168.6) | 168.9  (167.4 – 170.4) | 174.5  (173.0 – 176.0) | 176.8  (175.3 – 178.4) | 177.7  (176.1 – 179.2) |
| 40 | 148.9  (147.5 – 150.3) | 153.9  (152.4 – 155.3) | 151.7  (150.3 – 153.1) | 155.8  (154.3 – 157.3) | 156.8  (155.3 – 158.3) | 161.8  (160.3 – 163.3) | 163.8  (162.2 – 165.3) | 166.7  (165.1 – 168.2) |
| 50 | 164.5  (163.0 – 165.9) | 169.0  (167.6 – 170.5) | 166.0  (164.6 – 167.5) | 167.5  (166.1 – 168.9) | 166.7  (165.3 – 168.1) | 168.2  (166.8 – 169.7) | 167.6  (166.1 – 169.1) | 165.2  (163.8 – 166.7) |
| 60 | 177.5  (175.7 – 179.3) | 182.6  (180.8 – 184.4) | 183.6  (181.8 – 185.3) | 185.6  (183.9 – 187.4) | 184.7  (183.0 – 186.4) | 187.7  (186.0 – 189.3) | 189.7  (188.1 – 191.4) | 188.4  (186.8 – 190.0) |
| 70 | 220.3  (217.8 – 222.7) | 227.1  (224.6 – 229.6) | 223.8  (221.5 – 226.2) | 226.2  (223.8 – 228.5) | 222.5  (220.2 – 224.8) | 225.5  (223.2 – 227.8) | 224.9  (222.8 – 226.9) | 224.5  (222.5 – 226.5) |
| 80 | 309.5  (306.1 – 312.9) | 317.7  (314.2 – 321.2) | 312.6  (309.2 – 316.1) | 313.3  (309.8 – 316.7) | 312.6  (309.2 – 316) | 314.7  (311.4 – 318.1) | 312.9  (309.6 – 316.2) | 310.9  (307.6 – 314.2) |
| 90 | 378.8  (372.7 – 384.9) | 379.6  (373.7 – 385.6) | 381.8  (376 – 387.6) | 383.4  (377.7 – 389.1) | 385.5  (380.0 – 391.1) | 375.2  (369.8 – 380.6) | 376.6  (371.2 – 381.9) | 378.7  (373.4 – 384) |
| 100 | 116.1  (103.6 – 128.5) | 135.8  (122.4 – 149.1) | 131.0  (119.0 – 142.9) | 147.6  (135.4 – 159.8) | 130.8  (120.1 – 141.6) | 125.7  (115.2 – 136.2) | 137.8  (127.0 – 148.7) | 126.4  (116.3 – 136.5) |
| **Hospital admissions, rate per 1,000 people (95% CI)** | | | | | | | | |
| Overall | 49.3 (49.0 – 49.7) | 49.5 (49.1 – 49.8) | 49.6 (49.2 – 50.0) | 50.2 (49.9 – 50.6) | 50.1 (49.7 – 50.4) | 50.2 (49.8 – 50.5) | 51.2 (50.8 – 51.6) | 51.8 (51.4 – 52.2) |
| 20 | 21.2 (20.6 – 21.8) | 19.7 (19.1 – 20.2) | 19.5 (18.9 – 20.1) | 20.4 (19.8 – 21.0) | 21.0 (20.4 – 21.6) | 21.0 (20.3 – 21.6) | 20.5 (19.9 – 21.2) | 20.0 (19.3 – 20.6) |
| 30 | 54.7 (53.8 – 55.7) | 55.6 (54.7 – 56.6) | 55.5 (54.6 – 56.4) | 55.2 (54.2 – 56.1) | 54.6 (53.7 – 55.5) | 55.3 (54.4 – 56.2) | 55.1 (54.2 – 56.0) | 55.6 (54.6 – 56.5) |
| 40 | 25.6 (25.0 – 26.2) | 26.2 (25.5 – 26.8) | 24.9 (24.3 – 25.5) | 25.7 (25.1 – 26.4) | 26.0 (25.3 – 26.6) | 25.6 (25.0 – 26.2) | 25.5 (24.8 – 26.1) | 26.3 (25.6 – 26.9) |
| 50 | 29.6 (29.0 – 30.3) | 29.9 (29.3 – 30.6) | 28.9 (28.2 – 29.5) | 28.4 (27.8 – 29.1) | 27.9 (27.3 – 28.5) | 27.7 (27.0 – 28.3) | 26.5 (25.9 – 27.1) | 26.4 (25.7 – 27.0) |
| 60 | 52.6 (51.6 – 53.6) | 51.7 (50.7 – 52.7) | 52.0 (51.0 – 53.0) | 50.8 (49.8 – 51.8) | 49.8 (48.9 – 50.7) | 49.8 (48.8 – 50.7) | 48.6 (47.7 – 49.5) | 48.5 (47.7 – 49.4) |
| 70 | 90.0 (88.3 – 91.8) | 92.9 (91.2 – 94.7) | 91.3 (89.6 – 92.9) | 91.5 (89.9 – 93.1) | 88.0 (86.4 – 89.5) | 85.6 (84.1 – 87.1) | 86.7 (85.3 – 88.2) | 85.1 (83.8 – 86.5) |
| 80 | 155.2  (152.5 – 157.9) | 154.1  (151.4 – 156.8) | 152.5  (149.9 – 155.2) | 149.8  (147.2 – 152.5) | 148.0  (145.4 – 150.7) | 144.1  (141.6 – 146.7) | 143.7  (141.2 – 146.2) | 141.1  (138.7 – 143.6) |
| 90 | 222.0  (216.8 – 227.2) | 219.9  (214.9 – 225) | 218.3  (213.3 – 223.2) | 218  (213.2 – 222.9) | 215.9  (211.2 – 220.6) | 207.1  (202.6 – 211.6) | 208.7  (204.2 – 213.2) | 210.4  (205.9 – 214.8) |
| 100 | 64.9 (55.3 – 74.5) | 75.4 (65.1 – 85.7) | 81.5 (71.8 – 91.2) | 93.1  (83.1 – 103.1) | 80.1 (71.4 – 88.8) | 76.3 (67.9 – 84.7) | 85.8 (77.0 – 94.6) | 72.4 (64.5 – 80.3) |
| **ICU admissions, rate per 1,000 people (95% CI)** | | | | | | | | |
| Overall | 4.3 (4.2 – 4.4) | 4.5 (4.4 – 4.6) | 4.7 (4.6 – 4.8) | 4.6 (4.5 – 4.7) | 4.7 (4.6 – 4.8) | 4.6 (4.5 – 4.7) | 4.7 (4.6 – 4.8) | 4.9 (4.8 – 5.0) |
| 20 | 1.0 (0.8 – 1.1) | 1.0 (0.8 – 1.1) | 0.9 (0.8 – 1.1) | 1.1 (1.0 – 1.2) | 1.3 (1.1 – 1.4) | 1.3 (1.1 – 1.4) | 1.2 (1.1 – 1.4) | 1.3 (1.2 – 1.5) |
| 30 | 1.0 (0.8 – 1.1) | 1.1 (1.0 – 1.2) | 1.1 (1.0 – 1.2) | 1.1 (1.0 – 1.2) | 1.1 (1.0 – 1.3) | 1.3 (1.1 – 1.4) | 1.3 (1.2 – 1.5) | 1.3 (1.2 – 1.5) |
| 40 | 1.5 (1.3 – 1.6) | 1.7 (1.5 – 1.8) | 1.6 (1.4 – 1.7) | 1.5 (1.3 – 1.7) | 1.7 (1.6 – 1.9) | 1.7 (1.6 – 1.9) | 1.6 (1.4 – 1.7) | 1.7 (1.6 – 1.9) |
| 50 | 3.1 (2.9 – 3.3) | 3.3 (3.1 – 3.5) | 3.2 (3.0 – 3.5) | 3.2 (3.0 – 3.4) | 3.2 (3.0 – 3.4) | 3.2 (3.0 – 3.4) | 2.9 (2.7 – 3.1) | 3.1 (2.8 – 3.3) |
| 60 | 6.6 (6.2 – 7.0) | 6.7 (6.3 – 7.1) | 7.0 (6.6 – 7.4) | 6.7 (6.3 – 7.1) | 6.5 (6.2 – 6.9) | 6.6 (6.2 – 6.9) | 6.2 (5.9 – 6.5) | 6.3 (6.0 – 6.6) |
| 70 | 12.2 (11.6 – 12.9) | 13.2 (12.5 – 13.9) | 13.3 (12.7 – 14.0) | 12.9 (12.2 – 13.5) | 12.2 (11.6 – 12.8) | 11.4 (10.8 – 11.9) | 10.9 (10.4 – 11.4) | 11.3 (10.8 – 11.8) |
| 80 | 19.4 (18.4 – 20.4) | 19.7 (18.7 – 20.8) | 20.3 (19.3 – 21.3) | 19.2 (18.2 – 20.2) | 19.0 (18.0 – 20.0) | 17.1 (16.2 – 18.0) | 17.7 (16.7 – 18.6) | 17.5 (16.6 – 18.4) |
| 90 | 16.5 (14.9 – 18.1) | 17.0 (15.4 – 18.6) | 16.7 (15.1 – 18.2) | 15.6 (14.1 – 17.0) | 16.0 (14.5 – 17.4) | 14.6 (13.3 – 16.0) | 16.4 (15.0 – 17.8) | 16.1 (14.8 – 17.5) |
| 100 | 2.8 (0.7 – 4.8) | 4.7 (2.1 – 7.4) | 2.6 (0.8 – 4.4) | 1.5 (0.2 – 2.9) | 2.9 (1.2 – 4.6) | 2.9 (1.2 – 4.6) | 3.3 (1.5 – 5.1) | 2.2 (0.8 – 3.6) |
| **Receipt of invasive mechanical ventilation, rate per 1,000 people (95% CI)** | | | | | | | | |
| Overall | 2.3 (2.2 – 2.4) | 2.4 (2.3 – 2.5) | 2.7 (2.6 – 2.7) | 2.7 (2.6 – 2.8) | 2.7 (2.6 – 2.8) | 2.8 (2.7 – 2.9) | 2.9 (2.8 – 3.0) | 3.0 (2.9 – 3.1) |
| 20 | 0.4 (0.3 – 0.5) | 0.4 (0.3 – 0.5) | 0.5 (0.4 – 0.5) | 0.5 (0.4 – 0.6) | 0.7 (0.6 – 0.8) | 0.7 (0.6 – 0.8) | 0.7 (0.5 – 0.8) | 0.7 (0.5 – 0.8) |
| 30 | 0.4 (0.4 – 0.5) | 0.5 (0.4 – 0.6) | 0.6 (0.5 – 0.7) | 0.6 (0.5 – 0.7) | 0.6 (0.5 – 0.7) | 0.7 (0.6 – 0.8) | 0.7 (0.6 – 0.8) | 0.8 (0.7 – 0.9) |
| 40 | 0.7 (0.6 – 0.9) | 0.8 (0.7 – 0.9) | 0.8 (0.7 – 0.9) | 0.8 (0.7 – 0.9) | 1.0 (0.9 – 1.1) | 1.0 (0.8 – 1.1) | 1.0 (0.8 – 1.1) | 1.0 (0.9 – 1.1) |
| 50 | 1.8 (1.6 – 1.9) | 1.8 (1.6 – 1.9) | 1.9 (1.8 – 2.1) | 1.9 (1.7 – 2.1) | 1.9 (1.7 – 2.1) | 1.9 (1.8 – 2.1) | 1.7 (1.6 – 1.9) | 1.9 (1.7 – 2.1) |
| 60 | 3.8 (3.5 – 4.1) | 3.9 (3.7 – 4.2) | 4.3 (4.0 – 4.5) | 4.4 (4.1 – 4.7) | 4.3 (4.1 – 4.6) | 4.5 (4.2 – 4.7) | 4.2 (3.9 – 4.4) | 4.0 (3.8 – 4.3) |
| 70 | 7 .0 (6.5 – 7.5) | 7.8 (7.2 – 8.3) | 8.1 (7.6 – 8.6) | 8.2 (7.6 – 8.7) | 7.6 (7.1 – 8.1) | 7.5 (7.0 – 8.0) | 7.2 (6.8 – 7.6) | 7.7 (7.3 – 8.1) |
| 80 | 10.1 (9.4 – 10.9) | 10.0 (9.2 – 10.7) | 11.4 (10.6 – 12.2) | 10.5 (9.7 – 11.2) | 10.5 (9.8 – 11.3) | 10.2 (9.5 – 10.9) | 10.8 (10 – 11.5) | 10.7 (10.0 – 11.5) |
| 90 | 6.1 (5.1 – 7.1) | 6.0 (5.1 – 6.9) | 6.9 (5.9 – 7.9) | 6.1 (5.2 – 7.0) | 6.3 (5.4 – 7.2) | 7.2 (6.2 – 8.1) | 7.1 (6.1 – 8.0) | 7.5 (6.6 – 8.4) |
| 100 | 0.8 (0 – 1.8) | 1.2 (0 – 2.5) | 0.3 (0 – 0.9) | 0.3 (0 – 0.9) | 1.3 (0.2 – 2.5) | 1.3 (0.2 – 2.5) | 2.3 (0.8 – 3.8) | 1.2 (0.2 – 2.3) |
| **Death, rate per 1,000 people (95% CI)** | | | | | | | | |
| Overall | 6.6 (6.4 – 6.7) | 6.7 (6.5 – 6.8) | 6.7 (6.5 – 6.8) | 6.8 (6.7 – 6.9) | 7.1 (7 – 7.3) | 7.1 (7.0 – 7.3) | 7.5 (7.3 – 7.6) | 7.7 (7.5 – 7.8) |
| 20 | 0.3 (0.2 – 0.3) | 0.4 (0.3 – 0.4) | 0.2 (0.2 – 0.3) | 0.3 (0.2 – 0.3) | 0.3 (0.2 – 0.4) | 0.4 (0.3 – 0.5) | 0.4 (0.3 – 0.4) | 0.4 (0.3 – 0.5) |
| 30 | 0.3 (0.2 – 0.4) | 0.4 (0.3 – 0.5) | 0.4 (0.3 – 0.5) | 0.4 (0.3 – 0.4) | 0.4 (0.3 – 0.5) | 0.5 (0.5 – 0.6) | 0.6 (0.5 – 0.7) | 0.6 (0.5 – 0.7) |
| 40 | 0.6 (0.5 – 0.7) | 0.8 (0.7 – 0.9) | 0.7 (0.6 – 0.8) | 0.7 (0.6 – 0.8) | 0.8 (0.6 – 0.9) | 0.9 (0.7 – 1.0) | 0.8 (0.7 – 0.9) | 0.9 (0.8 – 1.0) |
| 50 | 2.1 (1.9 – 2.2) | 2.2 (2.0 – 2.3) | 2.0 (1.8 – 2.2) | 1.9 (1.7 – 2.0) | 2.1 (1.9 – 2.3) | 1.9 (1.8 – 2.1) | 1.8 (1.6 – 1.9) | 1.9 (1.7 – 2.0) |
| 60 | 5.4 (5.1 – 5.8) | 5.7 (5.3 – 6.0) | 5.8 (5.5 – 6.2) | 5.2 (4.8 – 5.5) | 5.6 (5.3 – 6.0) | 5.6 (5.3 – 6.0) | 5.2 (4.9 – 5.5) | 5.3 (5.0 – 5.6) |
| 70 | 14.4 (13.6 – 15.1) | 14.0 (13.3 – 14.7) | 13.5 (12.8 – 14.2) | 13.3 (12.7 – 14.0) | 13.1 (12.5 – 13.7) | 13.0 (12.4 – 13.6) | 13.3 (12.7 – 13.8) | 12.7 (12.2 – 13.2) |
| 80 | 38.7 (37.2 – 40.1) | 38.3 (36.9 – 39.7) | 37.5 (36.1 – 38.9) | 37.5 (36.1 – 38.9) | 37.3 (36.0 – 38.7) | 35.4 (34.1 – 36.8) | 35.0 (33.6 – 36.3) | 35.7 (34.4 – 37.1) |
| 90 | 110.2  (106.2 – 114.1) | 108.2  (104.4 – 111.9) | 103.9  (100.3 – 107.6) | 106.3  (102.6 – 109.9) | 108.5  (104.9 – 112.0) | 102.1  (98.7 – 105.4) | 106.0  (102.6 – 109.4) | 105.7  (102.4 – 109) |
| 100 | 96.4  (84.9 – 107.9) | 102.2  (90.4 – 114.0) | 96.6  (86.1 – 107.1) | 101.1  (90.7 – 111.5) | 94.9  (85.6 – 104.2) | 87.5 (78.6 – 96.5) | 99.2  (89.8 – 108.5) | 88.0 (79.4 – 96.7) |

|  | **2019** |
| --- | --- |
| **ED visits, rate per 1,000 people (95% CI)** | |
| Overall | 199.1 (198.4 – 199.8) |
| 20 | 224.1 (222.2 – 226.0) |
| 30 | 181.8 (180.2 – 183.3) |
| 40 | 167.8 (166.3 – 169.4) |
| 50 | 164.2 (162.7 – 165.6) |
| 60 | 188.0 (186.4 – 189.6) |
| 70 | 220.8 (218.7 – 222.8) |
| 80 | 307.4 (304.2 – 310.6) |
| 90 | 371.2 (366.0 – 376.4) |
| 100 | 123.6 (114.0 – 133.1) |
| **Hospital admissions, rate per 1,000 people (95% CI)** | |
| Overall | 51.5 (51.1 – 51.8) |
| 20 | 20.0 (19.3 – 20.6) |
| 30 | 56.3 (55.4 – 57.3) |
| 40 | 26.5 (25.9 – 27.2) |
| 50 | 25.7 (25.1 – 26.3) |
| 60 | 46.6 (45.8 – 47.5) |
| 70 | 83.8 (82.4 – 85.1) |
| 80 | 140.2 (137.8 – 142.6) |
| 90 | 206.1 (201.7 – 210.5) |
| 100 | 70.0 (62.6 – 77.5) |
| **ICU admissions, rate per 1,000 people (95% CI)** | |
| Overall | 4.9 (4.8 – 5.0) |
| 20 | 1.2 (1.1 – 1.4) |
| 30 | 1.3 (1.1 – 1.4) |
| 40 | 1.8 (1.6 – 2.0) |
| 50 | 3.0 (2.8 – 3.2) |
| 60 | 6.2 (5.9 – 6.5) |
| 70 | 11.1 (10.6 – 11.6) |
| 80 | 17.6 (16.7 – 18.6) |
| 90 | 15.9 (14.6 – 17.3) |
| 100 | 3.3 (1.6 – 5.0) |
| **Receipt of invasive mechanical ventilation, rate per 1,000 people (95% CI)** | |
| Overall | 2.9 (2.8 – 3.0) |
| 20 | 0.7 (0.6 – 0.8) |
| 30 | 0.7 (0.6 – 0.8) |
| 40 | 1.0 (0.9 – 1.1) |
| 50 | 1.8 (1.6 – 1.9) |
| 60 | 4.1 (3.8 – 4.3) |
| 70 | 7.3 (6.9 – 7.7) |
| 80 | 9.5 (8.8 – 10.1) |
| 90 | 7.4 (6.5 – 8.3) |
| 100 | 1.3 (0.3 – 2.4) |
| **Death, rate per 1,000 people (95% CI)** | |
| Overall | 7.6 (7.5 – 7.8) |
| 20 | 0.5 (0.4 – 0.6) |
| 30 | 0.5 (0.4 – 0.6) |
| 40 | 1.0 (0.9 – 1.1) |
| 50 | 2.0 (1.8 – 2.1) |
| 60 | 5.1 (4.8 – 5.3) |
| 70 | 12.3 (11.7 – 12.8) |
| 80 | 35.1 (33.8 – 36.4) |
| 90 | 102.9 (99.6 – 106.2) |
| 100 | 88.6 (80.3 – 96.8) |
