# Supplementary material for: Acute healthcare resource utilization by age: A cohort study
Source: PLoS One. 2021 May 19;16(5):e0251877. doi: 10.1371/journal.pone.0251877 (PMC8133481; doi:10.1371/journal.pone.0251877)
Supplement: S5 Table — Estimated annual percent change; a) Rates of acute healthcare and critical care encounters stratified by age; b) Absolute number of acute healthcare and critical care encounters stratified by age. (DOCX) [file pone.0251877.s009.docx]

**S5 Table.** Estimated annual percent change

1. Rates of acute healthcare and critical care encounters stratified by age

| **Age group** | **Estimated annual percent change, 95% CI** | | | |
| --- | --- | --- | --- | --- |
|  | **ED visits** | **Hospital admissions** | **ICU admissions** | **Invasive mechanical ventilation** |
| 20 | 2.30 (2.00 – 2.60) | -2.12 (-2.39 – -1.85) | 2.97 (2.13 – 3.82) | 3.38 (2.60 – 4.16) |
| 30 | 1.53 (1.42 – 1.64) | -0.32 (-0.53 – -0.11) | 2.73 (2.00 – 3.46) | 2.97 (2.15 – 3.79) |
| 40 | 1.00 (0.77 – 1.23) | -1.54 (-1.86 – -1.22) | 0.43 (-0.28 – 1.15) | 2.01 (1.55 – 2.47) |
| 50 | 0.55 (0.31 – 0.80) | -2.61 (-2.79 – -2.44) | -1.44 (-1.84 – -1.03) | 0.83 (0.53 – 1.12) |
| 60 | 0.61 (0.44 – 0.78) | -2.07 (-2.22 – -1.91) | -1.52 (-1.93 – -1.10) | 0.12 (-0.16 – 0.41) |
| 70 | 0.12 (-0.05 – 0.29) | -2.06 (-2.21 – -1.90) | -2.40 (-2.78 – -2.02) | -0.67 (-0.95 – -0.38) |
| 80 | 0.16 (-0.01 – 0.32) | -1.83 (-1.92 – -1.74) | -2.29 (-2.58 – -2.00) | 0.36 (0.04 – 0.68) |
| 90 | 0.80 (0.57 – 1.02) | -1.08 (-1.20 – -0.96) | -1.09 (-1.75 – -0.42) | 1.73 (1.07 – 2.40) |
| 100 | -1.47 (-2.52 – -0.42) | -3.26 (-3.90 – -2.62) | -3.20 (-6.60 – -0.32) | 1.74 (-2.73 – 6.41) |

1. Absolute number of acute healthcare and critical care encounters stratified by age

| **Age group** | **Estimated annual percent change, 95% CI** | | | |
| --- | --- | --- | --- | --- |
|  | **ED visits** | **Hospital admissions** | **ICU admissions** | **Invasive mechanical ventilation** |
| 20 | 0.92 (0.52 – 1.31) | -2.97 (-3.14 – -2.81) | 2.08 (1.38 – 2.78) | 2.12 (0.94 – 3.32) |
| 30 | 2.03 (1.84 – 2.23) | -1.01 (-1.43 – -0.59) | 3.53 (2.78 – 4.28) | 1.97 (0.79 – 3.15) |
| 40 | -0.35 (-0.74 – 0.04) | -2.01 (-2.34 – -1.68) | -0.60 (-1.47 – 0.27) | 1.80 (1.29 – 2.32) |
| 50 | 2.00 (1.18 – 2.83) | -0.51 (-0.79 – -0.23) | 0.26 (-0.34 – 0.87) | 3.19 (2.60 – 3.79) |
| 60 | 4.20 (3.74 – 4.66) | 1.43 (1.59 – 1.70) | 2.21 (1.81 – 2.60) | 4.09 (3.69 – 4.50) |
| 70 | 4.02 (3.52 – 4.53) | 0.22 (-0.38 – 0.82) | 1.47 (0.66 – 2.29) | 2.11 (1.46 – 2.76) |
| 80 | 2.10 (1.89 – 2.31) | 0.83 (0.54 – 1.12) | -0.37 (-0.71 – -0.03) | 3.47 (3.03 – 3.92) |
| 90 | 6.77 (6.00 – 7.54) | 4.63 (4.21 – 5.05) | 4.72 (3.61 – 5.84) | 7.69 (6.82 – 8.57) |
| 100 | 8.84 (7.87 – 9.82) | 7.57 (6.87 – 8.28) | 6.32 (2.74 – 10.02) | 12.91 (8.23 – 17.79) |
