# Supplementary material for: Acute healthcare resource utilization by age: A cohort study
Source: PLoS One. 2021 May 19;16(5):e0251877. doi: 10.1371/journal.pone.0251877 (PMC8133481; doi:10.1371/journal.pone.0251877)
Supplement: S6 Table — (DOCX) [file pone.0251877.s010.docx]

**S6 Table.** Annual number of events and total duration of events among patients who experienced at least one major healthcare encounter

|  | **1995** | **1996** | **1997** | **1998** | **1999** | **2000** | **2001** | **2002** |
| --- | --- | --- | --- | --- | --- | --- | --- | --- |
| **Number of ED visits, median (IQR)** | | | | | | | | |
| Overall | NA | NA | NA | NA | NA | NA | NA | NA |
| 20 | NA | NA | NA | NA | NA | NA | NA | NA |
| 30 | NA | NA | NA | NA | NA | NA | NA | NA |
| 40 | NA | NA | NA | NA | NA | NA | NA | NA |
| 50 | NA | NA | NA | NA | NA | NA | NA | NA |
| 60 | NA | NA | NA | NA | NA | NA | NA | NA |
| 70 | NA | NA | NA | NA | NA | NA | NA | NA |
| 80 | NA | NA | NA | NA | NA | NA | NA | NA |
| 90 | NA | NA | NA | NA | NA | NA | NA | NA |
| 100 | NA | NA | NA | NA | NA | NA | NA | NA |
| **Number of hospital admissions, median (IQR)** | | | | | | | | |
| Overall | 1 (1 – 1) | 1 (1 – 1) | 1 (1 – 1) | 1 (1 – 1) | 1 (1 – 1) | 1 (1 – 1) | 1 (1 – 1) | 1 (1 – 1) |
| 20 | 1 (1 – 1) | 1 (1 – 1) | 1 (1 – 1) | 1 (1 – 1) | 1 (1 – 1) | 1 (1 – 1) | 1 (1 – 1) | 1 (1 – 1) |
| 30 | 1 (1 – 1) | 1 (1 – 1) | 1 (1 – 1) | 1 (1 – 1) | 1 (1 – 1) | 1 (1 – 1) | 1 (1 – 1) | 1 (1 – 1) |
| 40 | 1 (1 – 1) | 1 (1 – 1) | 1 (1 – 1) | 1 (1 – 1) | 1 (1 – 1) | 1 (1 – 1) | 1 (1 – 1) | 1 (1 – 1) |
| 50 | 1 (1 – 1) | 1 (1 – 1) | 1 (1 – 1) | 1 (1 – 1) | 1 (1 – 1) | 1 (1 – 1) | 1 (1 – 1) | 1 (1 – 1) |
| 60 | 1 (1 – 1) | 1 (1 – 1) | 1 (1 – 2) | 1 (1 – 1) | 1 (1 – 1) | 1 (1 – 1) | 1 (1 – 1) | 1 (1 – 1) |
| 70 | 1 (1 – 2) | 1 (1 – 2) | 1 (1 – 2) | 1 (1 – 2) | 1 (1 – 2) | 1 (1 – 2) | 1 (1 – 2) | 1 (1 – 2) |
| 80 | 1 (1 – 2) | 1 (1 – 2) | 1 (1 – 2) | 1 (1 – 2) | 1 (1 – 2) | 1 (1 – 2) | 1 (1 – 2) | 1 (1 – 2) |
| 90 | 1 (1 – 2) | 1 (1 – 2) | 1 (1 – 2) | 1 (1 – 2) | 1 (1 – 2) | 1 (1 – 2) | 1 (1 – 2) | 1 (1 – 2) |
| 100 | 1 (1 – 1) | 1 (1 – 1) | 1 (1 – 1) | 1 (1 – 1) | 1 (1 – 1) | 1 (1 – 1) | 1 (1 – 1) | 1 (1 – 1) |
| **Annual days in hospital, median (IQR)** | | | | | | | | |
| Overall | 4 (2 – 9) | 4 (2 – 9) | 4 (2 – 9) | 4 (2 – 9) | 4 (2 – 9) | 4 (2 – 9) | 4 (2 – 9) | 4 (2 – 9) |
| 20 | 2 (1 – 4) | 2 (1 – 4) | 2 (1 – 4) | 2 (1 – 4) | 2 (1 – 4) | 2 (1 – 4) | 2 (1 – 4) | 2 (1 – 4) |
| 30 | 2 (2 – 4) | 2 (2 – 4) | 2 (1 – 4) | 2 (1 – 4) | 2 (1 – 4) | 2 (2 – 4) | 2 (2 – 4) | 2 (2 – 4) |
| 40 | 3 (2 – 6) | 3 (2 – 6) | 3 (2 – 6) | 3 (2 – 6) | 3 (2 – 6) | 3 (2 – 6) | 3 (2 – 6) | 3 (2 – 6) |
| 50 | 4 (2 – 8) | 4 (2 – 8) | 4 (2 – 8) | 4 (2 – 8) | 4 (2 – 8) | 4 (2 – 8) | 4 (2 – 7) | 4 (2 – 8) |
| 60 | 5 (2 – 11) | 5 (2 – 11) | 5 (2 – 10) | 5 (2 – 10) | 5 (2 – 10) | 5 (2 – 10) | 5 (2 – 10) | 5 (2 – 10) |
| 70 | 7 (3 – 15) | 7 (3 – 14) | 6 (3 – 14) | 6 (3 – 13) | 6 (3 – 14) | 6 (3 – 14) | 6 (3 – 13) | 6 (3 – 13) |
| 80 | 9 (4 – 19) | 8 (4 – 19) | 8 (4 – 18) | 8 (4 – 18) | 8 (4 – 18) | 8 (4 – 18) | 8 (4 – 18) | 8 (4 – 18) |
| 90 | 11 (5 – 23) | 10 (4 – 22) | 9 (5 – 20) | 10 (4 – 22) | 10 (4 – 21) | 10 (4 – 21) | 10 (5 – 21) | 10 (4 – 22) |
| 100 | 11 (4 – 18) | 9 (4 – 17) | 7 (2 – 19) | 9 (3 – 16) | 11 (5 – 22) | 9 (3 – 18) | 8 (4 – 19) | 11 (5 – 25) |
| **Number of ICU admissions, median (IQR)** | | | | | | | | |
| Overall | NA | NA | NA | NA | NA | NA | NA | NA |
| 20 | NA | NA | NA | NA | NA | NA | NA | NA |
| 30 | NA | NA | NA | NA | NA | NA | NA | NA |
| 40 | NA | NA | NA | NA | NA | NA | NA | NA |
| 50 | NA | NA | NA | NA | NA | NA | NA | NA |
| 60 | NA | NA | NA | NA | NA | NA | NA | NA |
| 70 | NA | NA | NA | NA | NA | NA | NA | NA |
| 80 | NA | NA | NA | NA | NA | NA | NA | NA |
| 90 | NA | NA | NA | NA | NA | NA | NA | NA |
| 100 | NA | NA | NA | NA | NA | NA | NA | NA |
| **Annual days in ICU, median (IQR)** | | | | | | | | |
| Overall | NA | NA | NA | NA | NA | NA | NA | NA |
| 20 | NA | NA | NA | NA | NA | NA | NA | NA |
| 30 | NA | NA | NA | NA | NA | NA | NA | NA |
| 40 | NA | NA | NA | NA | NA | NA | NA | NA |
| 50 | NA | NA | NA | NA | NA | NA | NA | NA |
| 60 | NA | NA | NA | NA | NA | NA | NA | NA |
| 70 | NA | NA | NA | NA | NA | NA | NA | NA |
| 80 | NA | NA | NA | NA | NA | NA | NA | NA |
| 90 | NA | NA | NA | NA | NA | NA | NA | NA |
| 100 | NA | NA | NA | NA | NA | NA | NA | NA |
| **Number of admissions associated with invasive mechanical ventilation, median (IQR)** | | | | | | | | |
| Overall | 1 (1 – 1) | 1 (1 – 1) | 1 (1 – 1) | 1 (1 – 1) | 1 (1 – 1) | 1 (1 – 1) | 1 (1 – 1) | 1 (1 – 1) |
| 20 | 1 (1 – 1) | 1 (1 – 1) | 1 (1 – 1) | 1 (1 – 1) | 1 (1 – 1) | 1 (1 – 1) | 1 (1 – 1) | 1 (1 – 1) |
| 30 | 1 (1 – 1) | 1 (1 – 1) | 1 (1 – 1) | 1 (1 – 1) | 1 (1 – 1) | 1 (1 – 1) | 1 (1 – 1) | 1 (1 – 1) |
| 40 | 1 (1 – 1) | 1 (1 – 1) | 1 (1 – 1) | 1 (1 – 1) | 1 (1 – 1) | 1 (1 – 1) | 1 (1 – 1) | 1 (1 – 1) |
| 50 | 1 (1 – 1) | 1 (1 – 1) | 1 (1 – 1) | 1 (1 – 1) | 1 (1 – 1) | 1 (1 – 1) | 1 (1 – 1) | 1 (1 – 1) |
| 60 | 1 (1 – 1) | 1 (1 – 1) | 1 (1 – 1) | 1 (1 – 1) | 1 (1 – 1) | 1 (1 – 1) | 1 (1 – 1) | 1 (1 – 1) |
| 70 | 1 (1 – 1) | 1 (1 – 1) | 1 (1 – 1) | 1 (1 – 1) | 1 (1 – 1) | 1 (1 – 1) | 1 (1 – 1) | 1 (1 – 1) |
| 80 | 1 (1 – 1) | 1 (1 – 1) | 1 (1 – 1) | 1 (1 – 1) | 1 (1 – 1) | 1 (1 – 1) | 1 (1 – 1) | 1 (1 – 1) |
| 90 | 1 (1 – 1) | 1 (1 – 1) | 1 (1 – 1) | 1 (1 – 1) | 1 (1 – 1) | 1 (1 – 1) | 1 (1 – 1) | 1 (1 – 1) |
| 100 | 1 (1 – 1) | 1 (1 – 1) | 1 (1 – 1) | 1 (1 – 1) | 1 (1 – 1) | 1 (1 – 1) | 2 (2 – 2) | 1 (1 – 1) |

|  | **2003** | **2004** | **2005** | **2006** | **2007** | **2008** | **2009** | **2010** |
| --- | --- | --- | --- | --- | --- | --- | --- | --- |
| **Number of ED visits, median (IQR)** | | | | | | | | |
| Overall | 1 (1 – 2) | 1 (1 – 2) | 1 (1 – 2) | 1 (1 – 2) | 1 (1 – 2) | 1 (1 – 2) | 1 (1 – 2) | 1 (1 – 2) |
| 20 | 1 (1 – 2) | 1 (1 – 2) | 1 (1 – 2) | 1 (1 – 2) | 1 (1 – 2) | 1 (1 – 2) | 1 (1 – 2) | 1 (1 – 2) |
| 30 | 1 (1 – 2) | 1 (1 – 2) | 1 (1 – 2) | 1 (1 – 2) | 1 (1 – 2) | 1 (1 – 2) | 1 (1 – 2) | 1 (1 – 2) |
| 40 | 1 (1 – 2) | 1 (1 – 2) | 1 (1 – 2) | 1 (1 – 2) | 1 (1 – 2) | 1 (1 – 2) | 1 (1 – 2) | 1 (1 – 2) |
| 50 | 1 (1 – 2) | 1 (1 – 2) | 1 (1 – 2) | 1 (1 – 2) | 1 (1 – 2) | 1 (1 – 2) | 1 (1 – 2) | 1 (1 – 2) |
| 60 | 1 (1 – 2) | 1 (1 – 2) | 1 (1 – 2) | 1 (1 – 2) | 1 (1 – 2) | 1 (1 – 2) | 1 (1 – 2) | 1 (1 – 2) |
| 70 | 1 (1 – 2) | 1 (1 – 2) | 1 (1 – 2) | 1 (1 – 2) | 1 (1 – 2) | 1 (1 – 2) | 1 (1 – 2) | 1 (1 – 2) |
| 80 | 1 (1 – 2) | 1 (1 – 2) | 1 (1 – 2) | 1 (1 – 2) | 1 (1 – 2) | 1 (1 – 2) | 1 (1 – 2) | 1 (1 – 2) |
| 90 | 1 (1 – 2) | 1 (1 – 2) | 1 (1 – 2) | 1 (1 – 2) | 1 (1 – 2) | 1 (1 – 2) | 1 (1 – 2) | 1 (1 – 2) |
| 100 | 1 (1 – 2) | 1 (1 – 2) | 1 (1 – 2) | 1 (1 – 2) | 1 (1 – 2) | 1 (1 – 2) | 1 (1 – 2) | 1 (1 – 2) |
| **Number of hospital admissions, median (IQR)** | | | | | | | | |
| Overall | 1 (1 – 1) | 1 (1 – 1) | 1 (1 – 1) | 1 (1 – 1) | 1 (1 – 1) | 1 (1 – 1) | 1 (1 – 1) | 1 (1 – 1) |
| 20 | 1 (1 – 1) | 1 (1 – 1) | 1 (1 – 1) | 1 (1 – 1) | 1 (1 – 1) | 1 (1 – 1) | 1 (1 – 1) | 1 (1 – 1) |
| 30 | 1 (1 – 1) | 1 (1 – 1) | 1 (1 – 1) | 1 (1 – 1) | 1 (1 – 1) | 1 (1 – 1) | 1 (1 – 1) | 1 (1 – 1) |
| 40 | 1 (1 – 1) | 1 (1 – 1) | 1 (1 – 1) | 1 (1 – 1) | 1 (1 – 1) | 1 (1 – 1) | 1 (1 – 1) | 1 (1 – 1) |
| 50 | 1 (1 – 1) | 1 (1 – 1) | 1 (1 – 1) | 1 (1 – 1) | 1 (1 – 1) | 1 (1 – 1) | 1 (1 – 1) | 1 (1 – 1) |
| 60 | 1 (1 – 1) | 1 (1 – 1) | 1 (1 – 1) | 1 (1 – 1) | 1 (1 – 1) | 1 (1 – 1) | 1 (1 – 1) | 1 (1 – 1) |
| 70 | 1 (1 – 2) | 1 (1 – 2) | 1 (1 – 2) | 1 (1 – 2) | 1 (1 – 1) | 1 (1 – 1) | 1 (1 – 1) | 1 (1 – 1) |
| 80 | 1 (1 – 2) | 1 (1 – 2) | 1 (1 – 2) | 1 (1 – 2) | 1 (1 – 2) | 1 (1 – 2) | 1 (1 – 2) | 1 (1 – 2) |
| 90 | 1 (1 – 2) | 1 (1 – 2) | 1 (1 – 2) | 1 (1 – 2) | 1 (1 – 2) | 1 (1 – 2) | 1 (1 – 2) | 1 (1 – 2) |
| 100 | 1 (1 – 1) | 1 (1 – 2) | 1 (1 – 1) | 1 (1 – 1) | 1 (1 – 1) | 1 (1 – 1) | 1 (1 – 1) | 1 (1 – 1) |
| **Annual days in hospital, median (IQR)** | | | | | | | | |
| Overall | 4 (2 – 9) | 4 (2 – 9) | 4 (2 – 9) | 3 (2 – 8) | 3 (2 – 8) | 3 (2 – 8) | 3 (2 – 8) | 3 (2 – 8) |
| 20 | 2 (1 – 4) | 2 (1 – 4) | 2 (1 – 4) | 2 (1 – 3) | 2 (1 – 3) | 2 (1 – 3) | 2 (1 – 3) | 2 (1 – 3) |
| 30 | 2 (1 – 3) | 2 (2 – 3) | 2 (1 – 3) | 2 (1 – 3) | 2 (1 – 3) | 2 (1 – 3) | 2 (1 – 3) | 2 (1 – 3) |
| 40 | 3 (1 – 6) | 3 (2 – 5) | 3 (1 – 5) | 3 (1 – 5) | 3 (1 – 4) | 3 (1 – 4) | 3 (1 – 4) | 2 (1 – 4) |
| 50 | 4 (2 – 8) | 4 (2 – 7) | 4 (2 – 7) | 3 (2 – 7) | 3 (2 – 6) | 3 (2 – 7) | 3 (2 – 7) | 3 (2 – 6) |
| 60 | 5 (2 – 10) | 4 (2 – 10) | 4 (2 – 9) | 4 (2 – 9) | 4 (2 – 8) | 4 (2 – 9) | 4 (2 – 8) | 4 (2 – 8) |
| 70 | 6 (3 – 13) | 6 (3 – 13) | 6 (3 – 12) | 5 (3 – 12) | 5 (3 – 12) | 5 (3 – 12) | 5 (3 – 11) | 5 (3 – 12) |
| 80 | 8 (4 – 18) | 8 (4 – 18) | 8 (4 – 17) | 8 (4 – 17) | 7 (4 – 17) | 7 (4 – 18) | 7 (3 – 17) | 7 (3 – 16) |
| 90 | 10 (4 – 20) | 10 (4 – 20) | 10 (4 – 20) | 9 (4 – 19) | 9 (4 – 20) | 10 (4 – 21) | 9 (4 – 20) | 9 (4 – 19) |
| 100 | 7 (4 – 14) | 9 (4 – 20) | 8 (3 – 17) | 9 (5 – 16) | 8 (4 – 18) | 8 (4 – 19) | 8 (4 – 17) | 8 (3 – 18) |
| **Number of ICU admissions, median (IQR)** | | | | | | | | |
| Overall | 1 (1 – 1) | 1 (1 – 1) | 1 (1 – 1) | 1 (1 – 1) | 1 (1 – 1) | 1 (1 – 1) | 1 (1 – 1) | 1 (1 – 1) |
| 20 | 1 (1 – 1) | 1 (1 – 1) | 1 (1 – 1) | 1 (1 – 1) | 1 (1 – 1) | 1 (1 – 1) | 1 (1 – 1) | 1 (1 – 1) |
| 30 | 1 (1 – 1) | 1 (1 – 1) | 1 (1 – 1) | 1 (1 – 1) | 1 (1 – 1) | 1 (1 – 1) | 1 (1 – 1) | 1 (1 – 1) |
| 40 | 1 (1 – 1) | 1 (1 – 1) | 1 (1 – 1) | 1 (1 – 1) | 1 (1 – 1) | 1 (1 – 1) | 1 (1 – 1) | 1 (1 – 1) |
| 50 | 1 (1 – 1) | 1 (1 – 1) | 1 (1 – 1) | 1 (1 – 1) | 1 (1 – 1) | 1 (1 – 1) | 1 (1 – 1) | 1 (1 – 1) |
| 60 | 1 (1 – 1) | 1 (1 – 1) | 1 (1 – 1) | 1 (1 – 1) | 1 (1 – 1) | 1 (1 – 1) | 1 (1 – 1) | 1 (1 – 1) |
| 70 | 1 (1 – 1) | 1 (1 – 1) | 1 (1 – 1) | 1 (1 – 1) | 1 (1 – 1) | 1 (1 – 1) | 1 (1 – 1) | 1 (1 – 1) |
| 80 | 1 (1 – 1) | 1 (1 – 1) | 1 (1 – 1) | 1 (1 – 1) | 1 (1 – 1) | 1 (1 – 1) | 1 (1 – 1) | 1 (1 – 1) |
| 90 | 1 (1 – 1) | 1 (1 – 1) | 1 (1 – 1) | 1 (1 – 1) | 1 (1 – 1) | 1 (1 – 1) | 1 (1 – 1) | 1 (1 – 1) |
| 100 | 1 (1 – 1) | 1 (1 – 1) | 1 (1 – 1) | 1 (1 – 1) | 1 (1 – 1) | 1 (1 – 1) | 1 (1 – 1) | 1 (1 – 1) |
| **Annual days in ICU, median (IQR)** | | | | | | | | |
| Overall | 3 (2 – 6) | 3 (2 – 6) | 3 (2 – 6) | 4 (2 – 6) | 3 (2 – 6) | 4 (2 – 7) | 4 (2 – 7) | 4 (2 – 7) |
| 20 | 3 (2 – 4) | 3 (2 – 4) | 3 (2 – 5) | 3 (2 – 5) | 3 (2 – 5) | 3 (2 – 4) | 3 (2 – 5) | 3 (2 – 5) |
| 30 | 2 (2 – 4) | 3 (2 – 5) | 3 (2 – 5) | 3 (2 – 5) | 3 (2 – 5) | 3 (2 – 5) | 3 (2 – 5) | 3 (2 – 6) |
| 40 | 3 (2 – 5) | 3 (2 – 5) | 3 (2 – 5) | 3 (2 – 6) | 3 (2 – 5) | 3 (2 – 7) | 3 (2 – 6) | 3 (2 – 6) |
| 50 | 3 (2 – 6) | 3 (2 – 5) | 3 (2 – 6) | 3 (2 – 6) | 3 (2 – 6) | 3 (2 – 7) | 4 (2 – 7) | 4 (2 – 7) |
| 60 | 3 (2 – 6) | 3 (2 – 6) | 3 (2 – 6) | 4 (2 – 6) | 3 (2 – 6) | 4 (2 – 7) | 4 (2 – 6) | 4 (2 – 7) |
| 70 | 4 (2 – 6) | 4 (2 – 6) | 4 (2 – 6) | 4 (2 – 7) | 4 (2 – 7) | 4 (2 – 7) | 4 (2 – 7) | 4 (2 – 7) |
| 80 | 4 (2 – 6) | 4 (2 – 6) | 4 (2 – 6) | 4 (2 – 6) | 4 (2 – 7) | 4 (2 – 7) | 4 (2 – 7) | 4 (3 – 7) |
| 90 | 3 (2 – 5) | 3 (2 – 6) | 3 (2 – 5) | 3 (2 – 5) | 3 (2 – 5) | 4 (2 – 6) | 4 (2 – 6) | 4 (2 – 6) |
| 100 | 2 (2 – 2) | 3 (2 – 8) | 5 (4 – 7) | 2 (1 – 5) | 4 (2 – 4) | 5 (3 – 5) | 3 (2 – 6) | 5 (2 – 8) |
| **Number of admissions associated with invasive mechanical ventilation, median (IQR)** | | | | | | | | |
| Overall | 1 (1 – 1) | 1 (1 – 1) | 1 (1 – 1) | 1 (1 – 1) | 1 (1 – 1) | 1 (1 – 1) | 1 (1 – 1) | 1 (1 – 1) |
| 20 | 1 (1 – 1) | 1 (1 – 1) | 1 (1 – 1) | 1 (1 – 1) | 1 (1 – 1) | 1 (1 – 1) | 1 (1 – 1) | 1 (1 – 1) |
| 30 | 1 (1 – 1) | 1 (1 – 1) | 1 (1 – 1) | 1 (1 – 1) | 1 (1 – 1) | 1 (1 – 1) | 1 (1 – 1) | 1 (1 – 1) |
| 40 | 1 (1 – 1) | 1 (1 – 1) | 1 (1 – 1) | 1 (1 – 1) | 1 (1 – 1) | 1 (1 – 1) | 1 (1 – 1) | 1 (1 – 1) |
| 50 | 1 (1 – 1) | 1 (1 – 1) | 1 (1 – 1) | 1 (1 – 1) | 1 (1 – 1) | 1 (1 – 1) | 1 (1 – 1) | 1 (1 – 1) |
| 60 | 1 (1 – 1) | 1 (1 – 1) | 1 (1 – 1) | 1 (1 – 1) | 1 (1 – 1) | 1 (1 – 1) | 1 (1 – 1) | 1 (1 – 1) |
| 70 | 1 (1 – 1) | 1 (1 – 1) | 1 (1 – 1) | 1 (1 – 1) | 1 (1 – 1) | 1 (1 – 1) | 1 (1 – 1) | 1 (1 – 1) |
| 80 | 1 (1 – 1) | 1 (1 – 1) | 1 (1 – 1) | 1 (1 – 1) | 1 (1 – 1) | 1 (1 – 1) | 1 (1 – 1) | 1 (1 – 1) |
| 90 | 1 (1 – 1) | 1 (1 – 1) | 1 (1 – 1) | 1 (1 – 1) | 1 (1 – 1) | 1 (1 – 1) | 1 (1 – 1) | 1 (1 – 1) |
| 100 | 1 (1 – 1) | 1 (1 – 1) | 1 (1 – 1) | 1 (1 – 1) | 1 (1 – 1) | 1 (1 – 1) | 1 (1 – 1) | 1 (1 – 1) |

|  | **2011** | **2012** | **2013** | **2014** | **2015** | **2016** | **2017** | **2018** |
| --- | --- | --- | --- | --- | --- | --- | --- | --- |
| **Number of ED visits, median (IQR)** | | | | | | | | |
| Overall | 1 (1 – 2) | 1 (1 – 2) | 1 (1 – 2) | 1 (1 – 2) | 1 (1 – 2) | 1 (1 – 2) | 1 (1 – 2) | 1 (1 – 2) |
| 20 | 1 (1 – 2) | 1 (1 – 2) | 1 (1 – 2) | 1 (1 – 2) | 1 (1 – 2) | 1 (1 – 2) | 1 (1 – 2) | 1 (1 – 2) |
| 30 | 1 (1 – 2) | 1 (1 – 2) | 1 (1 – 2) | 1 (1 – 2) | 1 (1 – 2) | 1 (1 – 2) | 1 (1 – 2) | 1 (1 – 2) |
| 40 | 1 (1 – 2) | 1 (1 – 2) | 1 (1 – 2) | 1 (1 – 2) | 1 (1 – 2) | 1 (1 – 2) | 1 (1 – 2) | 1 (1 – 2) |
| 50 | 1 (1 – 2) | 1 (1 – 2) | 1 (1 – 2) | 1 (1 – 2) | 1 (1 – 2) | 1 (1 – 2) | 1 (1 – 2) | 1 (1 – 2) |
| 60 | 1 (1 – 2) | 1 (1 – 2) | 1 (1 – 2) | 1 (1 – 2) | 1 (1 – 2) | 1 (1 – 2) | 1 (1 – 2) | 1 (1 – 2) |
| 70 | 1 (1 – 2) | 1 (1 – 2) | 1 (1 – 2) | 1 (1 – 2) | 1 (1 – 2) | 1 (1 – 2) | 1 (1 – 2) | 1 (1 – 2) |
| 80 | 1 (1 – 2) | 1 (1 – 2) | 1 (1 – 2) | 1 (1 – 2) | 1 (1 – 2) | 1 (1 – 2) | 1 (1 – 2) | 1 (1 – 2) |
| 90 | 1 (1 – 2) | 1 (1 – 2) | 1 (1 – 3) | 1 (1 – 3) | 2 (1 – 3) | 2 (1 – 3) | 1 (1 – 3) | 2 (1 – 3) |
| 100 | 1 (1 – 2) | 1 (1 – 2) | 1 (1 – 2) | 1 (1 – 2) | 1 (1 – 2) | 1 (1 – 2) | 1 (1 – 3) | 1 (1 – 2) |
| **Number of hospital admissions, median (IQR)** | | | | | | | | |
| Overall | 1 (1 – 1) | 1 (1 – 1) | 1 (1 – 1) | 1 (1 – 1) | 1 (1 – 1) | 1 (1 – 1) | 1 (1 – 1) | 1 (1 – 1) |
| 20 | 1 (1 – 1) | 1 (1 – 1) | 1 (1 – 1) | 1 (1 – 1) | 1 (1 – 1) | 1 (1 – 1) | 1 (1 – 1) | 1 (1 – 1) |
| 30 | 1 (1 – 1) | 1 (1 – 1) | 1 (1 – 1) | 1 (1 – 1) | 1 (1 – 1) | 1 (1 – 1) | 1 (1 – 1) | 1 (1 – 1) |
| 40 | 1 (1 – 1) | 1 (1 – 1) | 1 (1 – 1) | 1 (1 – 1) | 1 (1 – 1) | 1 (1 – 1) | 1 (1 – 1) | 1 (1 – 1) |
| 50 | 1 (1 – 1) | 1 (1 – 1) | 1 (1 – 1) | 1 (1 – 1) | 1 (1 – 1) | 1 (1 – 1) | 1 (1 – 1) | 1 (1 – 1) |
| 60 | 1 (1 – 1) | 1 (1 – 1) | 1 (1 – 1) | 1 (1 – 1) | 1 (1 – 1) | 1 (1 – 1) | 1 (1 – 1) | 1 (1 – 1) |
| 70 | 1 (1 – 1) | 1 (1 – 1) | 1 (1 – 1) | 1 (1 – 2) | 1 (1 – 2) | 1 (1 – 1) | 1 (1 – 1) | 1 (1 – 1) |
| 80 | 1 (1 – 2) | 1 (1 – 2) | 1 (1 – 2) | 1 (1 – 2) | 1 (1 – 2) | 1 (1 – 2) | 1 (1 – 2) | 1 (1 – 2) |
| 90 | 1 (1 – 2) | 1 (1 – 2) | 1 (1 – 2) | 1 (1 – 2) | 1 (1 – 2) | 1 (1 – 2) | 1 (1 – 2) | 1 (1 – 2) |
| 100 | 1 (1 – 1) | 1 (1 – 1) | 1 (1 – 1) | 1 (1 – 1) | 1 (1 – 1) | 1 (1 – 2) | 1 (1 – 2) | 1 (1 – 2) |
| **Annual days in hospital, median (IQR)** | | | | | | | | |
| Overall | 3 (2 – 8) | 3 (2 – 8) | 3 (2 – 8) | 3 (2 – 8) | 3 (2 – 8) | 3 (2 – 8) | 3 (2 – 8) | 3 (2 – 8) |
| 20 | 2 (1 – 3) | 2 (1 – 3) | 2 (1 – 3) | 2 (1 – 3) | 2 (1 – 3) | 2 (1 – 3) | 2 (1 – 3) | 2 (1 – 3) |
| 30 | 2 (1 – 3) | 2 (1 – 3) | 2 (1 – 3) | 2 (1 – 3) | 2 (1 – 3) | 2 (1 – 3) | 2 (1 – 3) | 2 (1 – 3) |
| 40 | 2 (1 – 4) | 2 (1 – 4) | 2 (1 – 4) | 2 (1 – 4) | 2 (1 – 4) | 2 (1 – 4) | 2 (1 – 4) | 2 (1 – 4) |
| 50 | 3 (2 – 7) | 3 (2 – 6) | 3 (2 – 6) | 3 (1 – 6) | 3 (2 – 6) | 3 (1 – 6) | 3 (1 – 6) | 3 (1 – 6) |
| 60 | 4 (2 – 8) | 4 (2 – 8) | 4 (2 – 8) | 4 (2 – 8) | 4 (2 – 8) | 3 (2 – 8) | 3 (2 – 8) | 3 (2 – 8) |
| 70 | 5 (3 – 11) | 5 (2 – 11) | 5 (2 – 11) | 5 (2 – 11) | 4 (2 – 11) | 4 (2 – 10) | 4 (2 – 10) | 4 (2 – 10) |
| 80 | 7 (3 – 16) | 7 (3 – 16) | 7 (3 – 16) | 7 (3 – 15) | 6 (3 – 15) | 6 (3 – 14) | 6 (3 – 14) | 6 (3 – 14) |
| 90 | 9 (4 – 19) | 9 (4 – 19) | 9 (4 – 18) | 9 (4 – 19) | 8 (4 – 18) | 8 (4 – 18) | 8 (4 – 18) | 8 (4 – 19) |
| 100 | 9 (4 – 20) | 7 (4 – 16) | 7 (3 – 13) | 7 (4 – 15) | 7 (3 – 18) | 7 (3 – 14) | 9 (4 – 16) | 9 (3 – 18) |
| **Number of ICU admissions, median (IQR)** | | | | | | | | |
| Overall | 1 (1 – 1) | 1 (1 – 1) | 1 (1 – 1) | 1 (1 – 1) | 1 (1 – 1) | 1 (1 – 1) | 1 (1 – 1) | 1 (1 – 1) |
| 20 | 1 (1 – 1) | 1 (1 – 1) | 1 (1 – 1) | 1 (1 – 1) | 1 (1 – 1) | 1 (1 – 1) | 1 (1 – 1) | 1 (1 – 1) |
| 30 | 1 (1 – 1) | 1 (1 – 1) | 1 (1 – 1) | 1 (1 – 1) | 1 (1 – 1) | 1 (1 – 1) | 1 (1 – 1) | 1 (1 – 1) |
| 40 | 1 (1 – 1) | 1 (1 – 1) | 1 (1 – 1) | 1 (1 – 1) | 1 (1 – 1) | 1 (1 – 1) | 1 (1 – 1) | 1 (1 – 1) |
| 50 | 1 (1 – 1) | 1 (1 – 1) | 1 (1 – 1) | 1 (1 – 1) | 1 (1 – 1) | 1 (1 – 1) | 1 (1 – 1) | 1 (1 – 1) |
| 60 | 1 (1 – 1) | 1 (1 – 1) | 1 (1 – 1) | 1 (1 – 1) | 1 (1 – 1) | 1 (1 – 1) | 1 (1 – 1) | 1 (1 – 1) |
| 70 | 1 (1 – 1) | 1 (1 – 1) | 1 (1 – 1) | 1 (1 – 1) | 1 (1 – 1) | 1 (1 – 1) | 1 (1 – 1) | 1 (1 – 1) |
| 80 | 1 (1 – 1) | 1 (1 – 1) | 1 (1 – 1) | 1 (1 – 1) | 1 (1 – 1) | 1 (1 – 1) | 1 (1 – 1) | 1 (1 – 1) |
| 90 | 1 (1 – 1) | 1 (1 – 1) | 1 (1 – 1) | 1 (1 – 1) | 1 (1 – 1) | 1 (1 – 1) | 1 (1 – 1) | 1 (1 – 1) |
| 100 | 1 (1 – 1) | 1 (1 – 1) | 1 (1 – 1) | 1 (1 – 1) | 1 (1 – 1) | 1 (1 – 1) | 1 (1 – 1) | 1 (1 – 1) |
| **Annual days in ICU, median (IQR)** | | | | | | | | |
| Overall | 4 (2 – 7) | 4 (2 – 7) | 4 (2 – 7) | 4 (2 – 7) | 4 (2 – 6) | 4 (2 – 7) | 4 (2 – 7) | 4 (2 – 7) |
| 20 | 3 (2 – 5) | 3 (2 – 6) | 3 (2 – 5) | 3 (2 – 6) | 3 (2 – 5) | 3 (2 – 5) | 3 (2 – 6) | 3 (2 – 5) |
| 30 | 3 (2 – 5) | 3 (2 – 5) | 3 (2 – 6) | 3 (2 – 5) | 3 (2 – 6) | 3 (2 – 6) | 3 (2 – 6) | 3 (2 – 6) |
| 40 | 3 (2 – 6) | 4 (2 – 6) | 3 (2 – 5) | 3 (2 – 5) | 4 (2 – 6) | 4 (2 – 7) | 4 (2 – 6) | 4 (2 – 6) |
| 50 | 3 (2 – 7) | 4 (2 – 6) | 4 (2 – 7) | 4 (2 – 7) | 3 (2 – 6) | 4 (2 – 7) | 3 (2 – 7) | 3 (2 – 6) |
| 60 | 4 (2 – 7) | 4 (2 – 8) | 4 (2 – 7) | 4 (2 – 7) | 4 (2 – 7) | 4 (2 – 7) | 4 (2 – 7) | 4 (2 – 7) |
| 70 | 4 (2 – 7) | 4 (2 – 7) | 4 (2 – 7) | 4 (2 – 7) | 4 (2 – 6) | 4 (2 – 7) | 4 (2 – 7) | 4 (2 – 8) |
| 80 | 4 (2 – 7) | 4 (2 – 7) | 4 (2 – 7) | 4 (2 – 7) | 4 (2 – 7) | 4 (2 – 7) | 4 (2 – 7) | 4 (2 – 7) |
| 90 | 4 (2 – 6) | 3 (2 – 6) | 4 (2 – 6) | 4 (2 – 6) | 3 (2 – 5) | 4 (2 – 6) | 3 (2 – 6) | 3 (2 – 6) |
| 100 | 4 (2 – 5) | 4 (2 – 5) | 2 (2 – 5) | 4 (2 – 5) | 3 (2 – 4) | 2 (2 – 6) | 3 (2 – 8) | 3 (2 – 4) |
| **Number of admissions associated with invasive mechanical ventilation, median (IQR)** | | | | | | | | |
| Overall | 1 (1 – 1) | 1 (1 – 1) | 1 (1 – 1) | 1 (1 – 1) | 1 (1 – 1) | 1 (1 – 1) | 1 (1 – 1) | 1 (1 – 1) |
| 20 | 1 (1 – 1) | 1 (1 – 1) | 1 (1 – 1) | 1 (1 – 1) | 1 (1 – 1) | 1 (1 – 1) | 1 (1 – 1) | 1 (1 – 1) |
| 30 | 1 (1 – 1) | 1 (1 – 1) | 1 (1 – 1) | 1 (1 – 1) | 1 (1 – 1) | 1 (1 – 1) | 1 (1 – 1) | 1 (1 – 1) |
| 40 | 1 (1 – 1) | 1 (1 – 1) | 1 (1 – 1) | 1 (1 – 1) | 1 (1 – 1) | 1 (1 – 1) | 1 (1 – 1) | 1 (1 – 1) |
| 50 | 1 (1 – 1) | 1 (1 – 1) | 1 (1 – 1) | 1 (1 – 1) | 1 (1 – 1) | 1 (1 – 1) | 1 (1 – 1) | 1 (1 – 1) |
| 60 | 1 (1 – 1) | 1 (1 – 1) | 1 (1 – 1) | 1 (1 – 1) | 1 (1 – 1) | 1 (1 – 1) | 1 (1 – 1) | 1 (1 – 1) |
| 70 | 1 (1 – 1) | 1 (1 – 1) | 1 (1 – 1) | 1 (1 – 1) | 1 (1 – 1) | 1 (1 – 1) | 1 (1 – 1) | 1 (1 – 1) |
| 80 | 1 (1 – 1) | 1 (1 – 1) | 1 (1 – 1) | 1 (1 – 1) | 1 (1 – 1) | 1 (1 – 1) | 1 (1 – 1) | 1 (1 – 1) |
| 90 | 1 (1 – 1) | 1 (1 – 1) | 1 (1 – 1) | 1 (1 – 1) | 1 (1 – 1) | 1 (1 – 1) | 1 (1 – 1) | 1 (1 – 1) |
| 100 | 1 (1 – 1) | 1 (1 – 1) | 2 (2 – 2) | 1 (1 – 1) | 1 (1 – 1) | 1 (1 – 1) | 1 (1 – 1) | 1 (1 – 1) |

|  | **2019** |
| --- | --- |
| **Number of ED visits, median (IQR)** | |
| Overall | 1 (1 – 2) |
| 20 | 1 (1 – 2) |
| 30 | 1 (1 – 2) |
| 40 | 1 (1 – 2) |
| 50 | 1 (1 – 2) |
| 60 | 1 (1 – 2) |
| 70 | 1 (1 – 2) |
| 80 | 1 (1 – 2) |
| 90 | 1 (1 – 3) |
| 100 | 1 (1 – 2) |
| **Number of hospital admissions, median (IQR)** | |
| Overall | 1 (1 – 1) |
| 20 | 1 (1 – 1) |
| 30 | 1 (1 – 1) |
| 40 | 1 (1 – 1) |
| 50 | 1 (1 – 1) |
| 60 | 1 (1 – 1) |
| 70 | 1 (1 – 1) |
| 80 | 1 (1 – 2) |
| 90 | 1 (1 – 2) |
| 100 | 1 (1 – 2) |
| **Annual days in hospital, median (IQR)** | |
| Overall | 3 (2 – 8) |
| 20 | 2 (1 – 3) |
| 30 | 2 (1 – 3) |
| 40 | 2 (1 – 4) |
| 50 | 3 (1 – 6) |
| 60 | 3 (2 – 8) |
| 70 | 4 (2 – 10) |
| 80 | 6 (3 – 14) |
| 90 | 8 (4 – 19) |
| 100 | 8 (4 – 17) |
| **Number of ICU admissions, median (IQR)** | |
| Overall | 1 (1 – 1) |
| 20 | 1 (1 – 1) |
| 30 | 1 (1 – 1) |
| 40 | 1 (1 – 1) |
| 50 | 1 (1 – 1) |
| 60 | 1 (1 – 1) |
| 70 | 1 (1 – 1) |
| 80 | 1 (1 – 1) |
| 90 | 1 (1 – 1) |
| 100 | 1 (1 – 1) |
| **Annual days in ICU, median (IQR)** | |
| Overall | 4 (2 – 7) |
| 20 | 3 (2 – 6) |
| 30 | 3 (2 – 6) |
| 40 | 3 (2 – 7) |
| 50 | 4 (2 – 7) |
| 60 | 4 (2 – 7) |
| 70 | 4 (2 – 7) |
| 80 | 4 (2 – 6) |
| 90 | 3 (2 – 6) |
| 100 | 4 (2 – 6) |
| **Number of admissions associated with invasive mechanical ventilation, median (IQR)** | |
| Overall | 1 (1 – 1) |
| 20 | 1 (1 – 1) |
| 30 | 1 (1 – 1) |
| 40 | 1 (1 – 1) |
| 50 | 1 (1 – 1) |
| 60 | 1 (1 – 1) |
| 70 | 1 (1 – 1) |
| 80 | 1 (1 – 1) |
| 90 | 1 (1 – 1) |
| 100 | 1 (1 – 1) |
