# Supplementary material for: Acute healthcare resource utilization by age: A cohort study
Source: PLoS One. 2021 May 19;16(5):e0251877. doi: 10.1371/journal.pone.0251877 (PMC8133481; doi:10.1371/journal.pone.0251877)
Supplement: S1 Appendix — (DOCX) [file pone.0251877.s011.docx]

| ICES DataThis Section must be Completed Prior to Project Dataset(s) Creation | |
| --- | --- |
| *The ICES Employee or agent who is responsible for creating the Project Dataset(s) must ensure that this list includes only data listed in the ICES Project PIA*  *Changes to this list after initial ICES Project PIA approval require an ICES Project PIA Amendment* | *Mandatory for all datasets that are available by individual year* |
| ***General Use Datasets – Health Services*** | ***Years (where applicable)*** |
| CIHI DAD | 1990-2019 (inclusive) |
| CIHI SDS | 1990-2019 (inclusive) |
| CCRS | 1990-2019 (inclusive) |
| CPRO | 1990-2019 (inclusive) |
| NACRS | 1990-2019 (inclusive) |
| ODB | 1990-2019 (inclusive) |
| OHIP | 1990-2019 (inclusive) |
| NRS | 1990-2019 (inclusive) |
| HCD | 1990-2019 (inclusive) |
| See list |  |
| See list |  |
| See list |  |
| ***General Use Datasets – Care Providers*** |  |
| See list |  |
| See list |  |
| ***General Use Datasets – Population*** |  |
| RPDB | 1990-2019 (inclusive) |
| POP | 1990-2019 (inclusive) |
| See list |  |
| ***General Use Datasets – Coding/Geography*** |  |
| PCCF | 1990-2015 (inclusive) |
| See list |  |
| ***General Use Datasets – Facilities*** |  |
| See list |  |
| ***General Use Datasets – Other*** |  |
| See list |  |
| See list |  |
| ***Controlled Use Datasets*** |  |
| See list |  |
| See list |  |
| ***Other Datasets*** |  |

| Project Cohort | | |
| --- | --- | --- |
| **Study Design** | ☒ Cohort study ☐ Matched cohort study ☐ Case-control study  ☐ Cross-sectional study ☐ Other (specify): | |
| **Index Event / Inclusion Criteria** | All persons in the RPDB age 20, 30, 40, 50, 60, 70, 80, 90, and 100 on Jan 1 of every year from 1995 to 2019 | |
| **Estimated Size of Cohort**  **(if known)** | 30 million | |
| **Exclusions (in order)** | *Step* | Description |
|  | 1 | No valid IKN recorded |
|  | 2 | Ontario non-resident |
|  | 3 | Invalid health card number |
|  | 4 | Missing sex |
|  |  |  |

| Project Time Frame Definitions | | |
| --- | --- | --- |
| Look-back Window  Observation Window  (in which to look for outcomes)  **Index Event Date**  Accrual Window  Max Follow-up Date | |  |
| **Accrual Start/End Dates** | Jan 1 1995 to Dec 31 2019 |  |
| **Max Follow-up Date** | Dec 31, 2019 |  |
| **When does observation window terminate?** | Observation windows will be as follows:   - All events that occur during the year the patient was included |  |
| **Lookback Window(s)** | 5 year look-back to identify comorbidities including chronic dialysis (defined below) |  |

| Variable Definitions (add additional rows as needed) | | |
| --- | --- | --- |
| **Main Exposure or Risk Factor** | Year and age (age as of Jan 1 of the index year) |  |
| **Primary Outcome Definition** | Admission to hospital   - Based on DAD (source is inpatient and acute is true) - count as binary outcome for the entire year (yes/no) - group acute admissions by hospital episodes |  |
| **Secondary Outcome Definition(s)** | All will be counted as binary (yes/no) as occurring one or more times during the year, then will count number of discrete events during the year.  Number of acute hospital episodes during the year (DAD)   - If a patient is hospitalized multiple times in the same year count each hospital episode   Duration of hospital admission (DAD)   - Total days in hospital for the year including data from all hospital episodes (includes both acute and ALC days)   Receipt of invasive mechanical ventilation   - The presence of one or more CCP code for mechanical ventilation for cases prior to 2002   **OR**   - The presence of one or more CCI code for mechanical ventilation for all cases from 2002 onward - See Appendix A for list of codes - A patient can only contribute one mechanical ventilation event per hospitalization (per hospital episode) - Keep as a binary outcome for the entire year (yes/no); and as the - Number of episodes of mechanical ventilation during year - count **one** instance of receipt of mechanical ventilation per unique hospital episode if CCP/CCI code presence during hospital episode   Duration of IMV (total duration for the entire year, including data from all hospitalizations)   - DAD   Admission to ICU   - Only available from 2002 onwards - Defined as the presence of a SCU code (Appendix B) - As above will not count readmissions to ICU during the same admission - Will count as binary outcome for the entire year (yes/no) and also if a patient is hospitalized multiple times in the same year and is admitted to the ICU on any of those hospitalizations we will count each episode   Total days in ICU admission (total count for the entire year, including data from all hospitalizations)   - DAD   Presentation to the emergency department   - NACRS (again count as binary outcome if patient present to ED or not during the year and also number of episodes that year) - Count both scheduled and unscheduled   Admission to rehab (NRS)   - Binary outcome, if ever admitted to rehab (yes/no) - Total number of times in a year that a patient is admitted to rehab - If NRS dataset does not go back far enough can we use discharge disposition and institution type?   Duration of rehab admission (total duration for the entire year, including all data from all rehab admissions)   - NRS   New admission to LTC (CPRO/DCRS)   - Exclude patients whose type of resident is long term care at start of year - Identify applications and placements/incident admissions   New receipt of home care services (HCD)   - Binary outcome, did they have any instance of receipt of new home care services in the year - Total number of nursing visits during the year - Total number of personal support worker visits during the year   Receipt of trach   - Based on OHIP billing code for trach (Z741) - If multiple trachs performed on the same patient during the same hospital episode will only count the first one   Receipt of new dialysis   - Based on the presence of a procedure code for acute dialysis (Appendix C) - Will exclude those identified as chronic dialysis   Underwent a MRI (yes/no)   - Based on OHIP claims codes   Total number of MRIs   - Total number of MRIs for the entire year   Underwent a CT (yes/no)   - Based on OHIP claims codes   Total number of CT scans   - Total number of CT scans for the entire year   Death   - RPDB   Location at time of death   - ICU - Acute care hospital (not in ICU) - Long term care facility - Home - Other |  |
| **Baseline Characteristics** | Chronic dialysis   - Defined as two or more dialysis codes in the past 3 years at least 90 days or more apart but no more than 150 days separating the most recent dialysis codes (dialysis codes in Appendix D) - Based off of Wald 2012 and Siddiqui 2012   Age (years)   - Based on RPDB   Sex (M/F)   - Based on RPDB   Type of primary resident   - Community, assisted living, nursing home   Pre-existing diagnosis of dementia   - Use pre-existing ICES code based on Jaakkimainen et al 2016 - A person 40 to 110 years of age who meets one of the following - Three OHIP claims claims with a dementia diagnosis recorded which were each **at least 30 days apart in a 2-year period (dementia codes in Appendix E)**   OR   - at least **one**hospitalization or same day surgery with a dementia diagnosis recorded   OR   - at least **one** ODB claim with a dementia medication  (SUBCLNAM= CHOLINESTERASE INHIBITORS) dispensed   Charlson score   - Based on 5-year look back and the Charlson-Deyo comorbidity index found in the DAD for the index admission   Previous or current receipt of home care services (HCD)   - Binary outcome, did they have any instance of receipt of home care during a 2-year look back period   Social economic status   - Based on neighbourhood income quintile, will use RPDB postal code and link to Stats Can (Stephenson 2011)   Rurality   - Urban vs rual, using RPDB |  |
| **Other Variables** |  |  |

| Analysis Plan and Dummy Tables (expand/modify as needed) |
| --- |
| **Descriptive Tables (insert or append dummy tables): Tables and figures on subsequent pages.**  **Statistical analysis as follows:**  **To quantify shifts in the risk of experiencing an acute care encounter over time, we calculated absolute and relative differences in rates of hospital admission and receipt of invasive mechanical ventilation, with 1995 as the baseline comparator. Standardized data related to ICU admissions were not routinely collected until 2002, therefore absolute and relative differences for rates of ICU admission were calculated using 2002 as the baseline comparator.**  **Primary model:** relative risk  **Primary independent value:** Year  **Dependent value:** Resource utilization (hospital admission, ICU admission, receipt of mechanical ventilation, death), separate formulates for each outcome  **No covariates**  **Secondary model, estimated percent annual change**  **Type of model:** negative binomial  **Primary independent value:** Year  **Dependent value:** Annual number of patients who experience a hospital admission (or ICU admission, or mechanical ventilation, or death), separate models for each outcome  **Offset:** the natural log of the population for the year of interest  **SAS formula to calculate estimated annual percent change:**  proc genmod data=*dataset*;  model Nmbr_Hosp_admns = year/dist=negbin link=log offset=log_population;  ods output parameterestimates = temp(where=(parameter='year'));  run;  data eapc (keep = eapc lower95 upper95 pvalue);  set temp;  eapc = (exp(estimate)-1)*100;  lower95 = (exp(lowerwaldcl)-1)*100;  upper95 = (exp(upperwaldcl)-1)*100;  pvalue = probchisq;  run;  proc print data = eapc;  var eapc lower95 upper95 pvalue;  run;  The above formula is the estimated annual percent change for the entire cohort, will repeat it stratified by age |
| **Table 1. Baseline population characteristics**   \|  \| **Total for all periods**  **(n = )** \| **1995**  **(n = )** \| **1996**  **(n = )** \| **1997**  **(n = )** \| **...** \| **2019**  **(n = )** \| \| --- \| --- \| --- \| --- \| --- \| --- \| --- \| \| **Age, n (%)** \|  \|  \|  \|  \|  \|  \| \| **20** \|  \|  \|  \|  \|  \|  \| \| **30** \|  \|  \|  \|  \|  \|  \| \| **40** \|  \|  \|  \|  \|  \|  \| \| **50** \|  \|  \|  \|  \|  \|  \| \| **60** \|  \|  \|  \|  \|  \|  \| \| **70** \|  \|  \|  \|  \|  \|  \| \| **80** \|  \|  \|  \|  \|  \|  \| \| **90** \|  \|  \|  \|  \|  \|  \| \| **100** \|  \|  \|  \|  \|  \|  \| \| **Female, N (%)** \|  \|  \|  \|  \|  \|  \| \| **Charlson comorbidity score, n (%)** \|  \|  \|  \|  \|  \|  \| \| **0** \|  \|  \|  \|  \|  \|  \| \| **1 – 2** \|  \|  \|  \|  \|  \|  \| \| **≥ 3** \|  \|  \|  \|  \|  \|  \| \| **No hospitalization within previous 5 years** \|  \|  \|  \|  \|  \|  \| \| **Chronic dialysis, N (%)** \|  \|  \|  \|  \|  \|  \| \| **History of dementia, N (%)** \|  \|  \|  \|  \|  \|  \| \| **Resident of a long-term care facility, n (%)** \|  \|  \|  \|  \|  \|  \| \| **Rural resident, n (%)** \|  \|  \|  \|  \|  \|  \| |

**Supplementary Table 1. Proportion of patients admitted experiencing a major healthcare per year stratified by age and sex**

|  | 1995 | | 1996 | | ... | |
| --- | --- | --- | --- | --- | --- | --- |
|  | Women | Men | Women | Men | Women | Men |
| Hospital admission, n (%) |  |  |  |  |  |  |
| Overall |  |  |  |  |  |  |
| 20 |  |  |  |  |  |  |
| 30 |  |  |  |  |  |  |
| 40 |  |  |  |  |  |  |
| 50 |  |  |  |  |  |  |
| 60 |  |  |  |  |  |  |
| 70 |  |  |  |  |  |  |
| 80 |  |  |  |  |  |  |
| 90 |  |  |  |  |  |  |
| 100 |  |  |  |  |  |  |
| ICU admissions, n (%) |  |  |  |  |  |  |
| Overall |  |  |  |  |  |  |
| 20 |  |  |  |  |  |  |
| 30 |  |  |  |  |  |  |
| 40 |  |  |  |  |  |  |
| 50 |  |  |  |  |  |  |
| 60 |  |  |  |  |  |  |
| 70 |  |  |  |  |  |  |
| 80 |  |  |  |  |  |  |
| 90 |  |  |  |  |  |  |
| 100 |  |  |  |  |  |  |
| Receipt of IMV, n (%) |  |  |  |  |  |  |
| Overall |  |  |  |  |  |  |
| 20 |  |  |  |  |  |  |
| 30 |  |  |  |  |  |  |
| 40 |  |  |  |  |  |  |
| 50 |  |  |  |  |  |  |
| 60 |  |  |  |  |  |  |
| 70 |  |  |  |  |  |  |
| 80 |  |  |  |  |  |  |
| 90 |  |  |  |  |  |  |
| 100 |  |  |  |  |  |  |
| Deaths, n (%) |  |  |  |  |  |  |
| Overall |  |  |  |  |  |  |
| 20 |  |  |  |  |  |  |
| 30 |  |  |  |  |  |  |
| 40 |  |  |  |  |  |  |
| 50 |  |  |  |  |  |  |
| 60 |  |  |  |  |  |  |
| 70 |  |  |  |  |  |  |
| 80 |  |  |  |  |  |  |
| 90 |  |  |  |  |  |  |
| 100 |  |  |  |  |  |  |

**Supplementary Table 2. Average volume of people experiencing a major healthcare encounters per year**

|  | Overall | 1995 | 1996 | ... | 2019 |
| --- | --- | --- | --- | --- | --- |
| Hospital admission, n (%) |  |  |  |  |  |
| ICU admission, n (%) ^†^ |  | N/A | N/A |  |  |
| Receipt of IMV, n (%) |  |  |  |  |  |
| Death, n (%) |  |  |  |  |  |

^†^ ICU admissions only captured from 2002 onwards

**Supplementary Table 3. Annual rates of each outcome of interest**

|  | **1995**  **(n = )** | **1996**  **(n = )** | **1997**  **(n = )** | **1998**  **(n = )** | **...** | **2019**  **(n = )** |
| --- | --- | --- | --- | --- | --- | --- |
| **Patients admitted to hospital, per 1,000 (95% CI)** |  |  |  |  |  |  |
| **Overall** |  |  |  |  |  |  |
| **People age 20 as of Jan. 1** |  |  |  |  |  |  |
| **People age 30 as of Jan. 1** |  |  |  |  |  |  |
| **People age 40 as of Jan. 1** |  |  |  |  |  |  |
| **People age 50 as of Jan. 1** |  |  |  |  |  |  |
| **People age 60 as of Jan. 1** |  |  |  |  |  |  |
| **People age 70 as of Jan. 1** |  |  |  |  |  |  |
| **People age 80 as of Jan. 1** |  |  |  |  |  |  |
| **People age 90 as of Jan. 1** |  |  |  |  |  |  |
| **People age 100 as of Jan. 1** |  |  |  |  |  |  |
| **Patients admitted to ICU, per 1,000 (95% CI)** |  |  |  |  |  |  |
| **Overall** |  |  |  |  |  |  |
| **People age 20 as of Jan. 1** |  |  |  |  |  |  |
| **People age 30 as of Jan. 1** |  |  |  |  |  |  |
| **People age 40 as of Jan. 1** |  |  |  |  |  |  |
| **People age 50 as of Jan. 1** |  |  |  |  |  |  |
| **People age 60 as of Jan. 1** |  |  |  |  |  |  |
| **People age 70 as of Jan. 1** |  |  |  |  |  |  |
| **People age 80 as of Jan. 1** |  |  |  |  |  |  |
| **People age 90 as of Jan. 1** |  |  |  |  |  |  |
| **People age 100 as of Jan. 1** |  |  |  |  |  |  |
| **Patients receiving mechanical ventilation, per 1,000 (95% CI)** |  |  |  |  |  |  |
| **Overall** |  |  |  |  |  |  |
| **People age 20 as of Jan. 1** |  |  |  |  |  |  |
| **People age 30 as of Jan. 1** |  |  |  |  |  |  |
| **People age 40 as of Jan. 1** |  |  |  |  |  |  |
| **People age 50 as of Jan. 1** |  |  |  |  |  |  |
| **People age 60 as of Jan. 1** |  |  |  |  |  |  |
| **People age 70 as of Jan. 1** |  |  |  |  |  |  |
| **People age 80 as of Jan. 1** |  |  |  |  |  |  |
| **People age 90 as of Jan. 1** |  |  |  |  |  |  |
| **People age 100 as of Jan. 1** |  |  |  |  |  |  |
| **Deaths, per 1,000 (95% CI)** |  |  |  |  |  |  |
| **Overall** |  |  |  |  |  |  |
| **People age 20 as of Jan. 1** |  |  |  |  |  |  |
| **People age 30 as of Jan. 1** |  |  |  |  |  |  |
| **People age 40 as of Jan. 1** |  |  |  |  |  |  |
| **People age 50 as of Jan. 1** |  |  |  |  |  |  |
| **People age 60 as of Jan. 1** |  |  |  |  |  |  |
| **People age 70 as of Jan. 1** |  |  |  |  |  |  |
| **People age 80 as of Jan. 1** |  |  |  |  |  |  |
| **People age 90 as of Jan. 1** |  |  |  |  |  |  |
| **People age 100 as of Jan. 1** |  |  |  |  |  |  |

**Supplementary Table 4. Frequency and duration of each outcome of interest in those who experienced the outcome of interest**

|  | **1995**  **(n = )** | **1996**  **(n = )** | **1997**  **(n = )** | **1998**  **(n = )** | **...** | **2019**  **(n = )** |
| --- | --- | --- | --- | --- | --- | --- |
| **Number of hospitals admissions per year, median (IQR)** |  |  |  |  |  |  |
| **Overall** |  |  |  |  |  |  |
| **People age 20 as of Jan. 1** |  |  |  |  |  |  |
| **People age 30 as of Jan. 1** |  |  |  |  |  |  |
| **People age 40 as of Jan. 1** |  |  |  |  |  |  |
| **People age 50 as of Jan. 1** |  |  |  |  |  |  |
| **People age 60 as of Jan. 1** |  |  |  |  |  |  |
| **People age 70 as of Jan. 1** |  |  |  |  |  |  |
| **People age 80 as of Jan. 1** |  |  |  |  |  |  |
| **People age 90 as of Jan. 1** |  |  |  |  |  |  |
| **People age 100 as of Jan. 1** |  |  |  |  |  |  |
| **Total duration of hospital admission per year, median days (IQR)** |  |  |  |  |  |  |
| **Overall** |  |  |  |  |  |  |
| **People age 20 as of Jan. 1** |  |  |  |  |  |  |
| **People age 30 as of Jan. 1** |  |  |  |  |  |  |
| **People age 40 as of Jan. 1** |  |  |  |  |  |  |
| **People age 50 as of Jan. 1** |  |  |  |  |  |  |
| **People age 60 as of Jan. 1** |  |  |  |  |  |  |
| **People age 70 as of Jan. 1** |  |  |  |  |  |  |
| **People age 80 as of Jan. 1** |  |  |  |  |  |  |
| **People age 90 as of Jan. 1** |  |  |  |  |  |  |
| **People age 100 as of Jan. 1** |  |  |  |  |  |  |
| **Number of ICU admissions per year, median (IQR)** |  |  |  |  |  |  |
| **Overall** | N/A | N/A | N/A | N/A |  |  |
| **People age 20 as of Jan. 1** | N/A | N/A | N/A | N/A |  |  |
| **People age 30 as of Jan. 1** | N/A | N/A | N/A | N/A |  |  |
| **People age 40 as of Jan. 1** | N/A | N/A | N/A | N/A |  |  |
| **People age 50 as of Jan. 1** | N/A | N/A | N/A | N/A |  |  |
| **People age 60 as of Jan. 1** | N/A | N/A | N/A | N/A |  |  |
| **People age 70 as of Jan. 1** | N/A | N/A | N/A | N/A |  |  |
| **People age 80 as of Jan. 1** | N/A | N/A | N/A | N/A |  |  |
| **People age 90 as of Jan. 1** | N/A | N/A | N/A | N/A |  |  |
| **People age 100 as of Jan. 1** | N/A | N/A | N/A | N/A |  |  |
| **Total duration of ICU admission per year, median days (IQR)** |  |  |  |  |  |  |
| **Overall** | N/A | N/A | N/A | N/A |  |  |
| **People age 20 as of Jan. 1** | N/A | N/A | N/A | N/A |  |  |
| **People age 30 as of Jan. 1** | N/A | N/A | N/A | N/A |  |  |
| **People age 40 as of Jan. 1** | N/A | N/A | N/A | N/A |  |  |
| **People age 50 as of Jan. 1** | N/A | N/A | N/A | N/A |  |  |
| **People age 60 as of Jan. 1** | N/A | N/A | N/A | N/A |  |  |
| **People age 70 as of Jan. 1** | N/A | N/A | N/A | N/A |  |  |
| **People age 80 as of Jan. 1** | N/A | N/A | N/A | N/A |  |  |
| **People age 90 as of Jan. 1** | N/A | N/A | N/A | N/A |  |  |
| **People age 100 as of Jan. 1** | N/A | N/A | N/A | N/A |  |  |

**Figure 1a-c. Overall rates of resource utilization**

Ex.

**Figures 2a-c. Trends in overall rates of resource utilization**

**Ex. *note the final version of the figure will include every year as opposed to every 5 years as shown in the example**

**Figures 3**

**a. Trends in overall rates of hospital admissions stratified by age**

**Ex. As above will utilize all years**

**a. Trends in overall number of hospital admissions stratified by age**

**Ex. As above will utilize all years**

**Figures 4 and 5 will repeat figure 3 for ICU admission and mechanical ventilation respectively**

**Supplemetary figures 1: Overall mortality rate stratified by age**

**Ex:**

**Supplemetary figures 2: Annual trends in mortality rate**

Ex. Final version will use every year, not every 5 years as shown in example

**Supplemetary figures 3: Trends in rates of hospital admissions stratified by age and sex**

**a. Women**

**ex. As above final version will use every year**

**b. Men**

**ex. As above final version will use every year**

**Supplemetary figures 4a and b: Trends in mortality rates stratified by age and trends in crude number of deaths stratified by age**

| Quality Assurance Activities | | | |
| --- | --- | --- | --- |
| **RAE Directory of SAS Programs** |  | | |
| **RAE Directory of Final Dataset(s)** | *The* *final analytic dataset for each cohort includes all the data required to create the baseline tables and run all the models. It should include all covariates for all models such as patient risk factors, hospital characteristics, physician characteristics, exposure measures (continuous, categorical) and outcomes. It should include covariates that were considered but didn’t make the final cut. This would permit an analyst to easily re-run the models in the future.* | | |
|  |  | | |
| **RAE README file available:** ☐Yes ☐No | | | |
| **Date results of quality assurance tools for final dataset shared with project team (where applicable):** | | |  |
|  | | **%assign** | yyyy-mon-dd |
|  | | **%evolution** | yyyy-mon-dd |
|  | | **%dinexplore** | yyyy-mon-dd |
|  | | **%track / %exclude** | yyyy-mon-dd |
|  | | **%codebook** | yyyy-mon-dd |
| **Additional comments:** | |  | |

Appendix A:

Mechanical ventilation

- CCP codes
  - 13.61 intermittent positive pressure breathing (IPPB)
  - 13.62 other mechanical assistance to respiration
- CCI codes (used with ICD-10)
  - 1.GZ.31.CA-ND – ventilation, respiratory system NEC positive pressure – invasive per orifice approach by endotracheal intubation
  - 1.GZ.31.CR-ND – same, but through tracheostomy
  - 1.GZ.31.GP-ND – same, but through jet ventilation

Based on Scales et al 2006, algorithm with the highest sensitivity

Appendix B

MSICU code

- SCU code 10 - medical intensive care nursing unit
- SCU code 20 – surgical intensive care nursing unit
- SCU code 30 – combined medical/surgical intensive care nursing unit

Based on Scales et al 2006, algorithm with the highest sensitivity

Appendix C

Acute dialysis procedure codes

- R849 – hemodialysis
- G323 – hemodialysis
- G325 – hemodialysis
- G866 – hemodialysis
- 1PZ21HQBR – hemodialysis
- 51.95 – hemodialysis
- G330 – Peritoneal dialysis
- G331 – Peritoneal dialysis
- 66.98 – Peritoneal dialysis
- 1PZ21HPD4 – Peritoneal dialysis
- G082 – Continuous renal replacement therapy
- G083 – Continuous renal replacement therapy
- G085 – Continuous renal replacement therapy
- G090 – Continuous renal replacement therapy
- G091 – Continuous renal replacement therapy
- G092 – Continuous renal replacement therapy
- G093 – Continuous renal replacement therapy
- G095 – Continuous renal replacement therapy
- G294 – Continuous renal replacement therapy
- G295 – Continuous renal replacement therapy
- 1PZ21HQBS – Continuous renal replacement therapy

Based on Siqqiqui et al 2012

Appendix D

Dialysis codes used to determine chronic dialysis

ICD 9 Dialysis diagnostic codes: V45.1, V56.0, V56.8, 36104

ICD 9 Dialysis procedural codes: 51.27, 51.42, 51.43, 51.95, 66.98

ICD 10 Dialysis diagnostic codes: T824, Y602, Y612, Y622, Y841, Z490, Z491, Z492, Z992, E10220, E10221, E10222, E10223, E10224, E10229, N180, E11220, E11221, E11222, E11223, E11224, E11229, E13220, E13221, E13222, E13223, E13224, E13229, E14220, E14221, E14222, E14223, E14224, E14229

ICD 10 Dialysis procedural codes: 1OT53DATS, 1OT53HATS, 1OT53LATS, 1PZ21HPD4, 1PZ21HQBR, 1SY55LAFT, 7SC59QD, 1KY76LA, 1KY76LASJ, 1KY76LAXXA, 1KY76LAXXL, 1KY76LAXXN, 1KY80LA, 1KY80LAXXA, 1KY80LAXXK, 1KY80LAXXN, 1KG76MZXXA, 1KG76MZXXN

OHIP Diagnostic codes + All Procedure Codes (OHIP, CCI, CCP) Hemodialysis: R849, R850, G323, G324, G336, G325, G326, G327, G860, G862, G863, G865, G866, G099, R825, R826, R827, R833, R840, R841, R843, R848, R851, Z450, Z451, Z452



OHIP Diagnostic codes + All Procedure Codes (OHIP, CCI, CCP) Peritoneal dialysis: G330, G331, G332, G861, G864, R852, R853, R854, R885

OHIP Diagnostic codes + All Procedure Codes (OHIP, CCI, CCP) Continuous renal replacement: G082, G083, G085, G090, G091, G092, G093, G094, G095, G096, G294, G295

OHIP Diagnostic codes + All Procedure Codes (OHIP, CCI, CCP) Misc dialysis codes: G333, H540, H740

Based on Siqqiqui et al 2012

OHIP ICU Treatment codes

- G557 – day 1 comprehensive care
- G558 – day 2-30 comprehensive care
- G559 – day 31 and onwad comprehensive care
- G400 – day 1 intensive care management excluding mechanical ventilation
- G401 – day 2-30 intensive care management excluding mechanical ventilation
- G402 – day 31 and onward intensive care management excluding mechanical ventilation
- G405 – day 1 intensive care management of mechanical ventilation
- G406 – day 2-30 intensive care management of mechanical ventilation
- G407 – day 31 and onward intensive care management of mechanical ventilation
- C101 – ICU premium for patient seen on a visit in ICU

Based on Scales et al 2006, algorithm with the highest sensitivity

**Appendix E**

Dementia Codes

**Diagnosis codes used for DAD/SDS**

ICD-9                    Description

46.1x                    Creutzfeldt-Jakob disease

290.0x                  Senile dementia, uncomplicated

290.1x                  Presenile dementia

290.2x                  Senile dementia with delusional or depressive features

290.3x                  Senile dementia with delirium

290.4x                  Vascular dementia

294.x                    Persistent mental disorders due to conditions classified elsewhere

331.0x                  Alzheimer's disease

331.1x                  Frontotemporal dementia

331.5x                  Idiopathic normal pressure hydrocephalus

ICD-10                  Description

F00.x                    Dementia in Alzheimer's disease

F01.x                    Vascular dementia

F02.x                    Dementia in other diseases classified elsewhere

F03.x                    Unspecified dementia

G30.x                    Alzheimer's disease

**Diagnosis codes used for OHIP**

ICD-9                    Description

290                       Dementias

331                       Other cerebral degenerations

**ODB**

Drug subclass:  Cholinesterase inhibitors
